# Comparative transcriptome analysis reveals gene expression differences between two peach cultivars under saline-alkaline stress

**DOI:** 10.1186/s41065-020-00122-4

**Published:** 2020-03-31

**Authors:** Shuxia Sun, Haiyan Song, Jing Li, Dong Chen, Meiyan Tu, Guoliang Jiang, Guoqing Yu, Zhiqin Zhou

**Affiliations:** 1 College of Horticulture and Landscape Architecture, Southwest University, Chongqing, 400716 China; 2 Horticulture Research Institute, Sichuan Academy of Agricultural Sciences, Chengdu, 610066 Sichuan Province China; 3 Fruit Technology Promotion Station of Longquanyi District, Chengdu, 610100 Sichuan Province China

**Keywords:** Peach, Saline-alkaline stress, Transmission electron microscopy observation, Comparative transcriptome analysis, Transcription factor

## Abstract

**Background:**

Saline-alkaline stress is a major abiotic stress that is harmful to plant growth worldwide. Two peach cultivars (GF677 and Maotao) display distinct phenotypes under saline-alkaline stress. The molecular mechanism explaining the differences between the two cultivars is still unclear.

**Results:**

In the present study, we systematically analysed the changes in GF677 and Maotao leaves upon saline-alkaline stress by using cytological and biochemical technologies as well as comparative transcriptome analysis. Transmission electron microscopy (TEM) observations showed that the structure of granum was dispersive in Maotao chloroplasts. The biochemical analysis revealed that POD activity and the contents of chlorophyll a and chlorophyll b, as well as iron, were notably decreased in Maotao. Comparative transcriptome analysis detected 881 genes with differential expression (including 294 upregulated and 587 downregulated) under the criteria of |log2 Ratio| ≥ 1 and FDR ≤0.01. Gene ontology (GO) analysis showed that all differentially expressed genes (DEGs) were grouped into 30 groups. MapMan annotation of DEGs showed that photosynthesis, antioxidation, ion metabolism, and WRKY TF were activated in GF677, while cell wall degradation, secondary metabolism, starch degradation, MYB TF, and bHLH TF were activated in Maotao. Several iron and stress-related TFs (*ppa024966m*, *ppa010295m*, *ppa0271826m*, *ppa002645m*, *ppa010846m*, *ppa009439m*, *ppa008846m*, and *ppa007708m*) were further discussed from a functional perspective based on the phylogenetic tree integration of other species homologues.

**Conclusions:**

According to the cytological and molecular differences between the two cultivars, we suggest that the integrity of chloroplast structure and the activation of photosynthesis as well as stress-related genes are crucial for saline-alkaline resistance in GF677. The results presented in this report provide a theoretical basis for cloning saline-alkaline tolerance genes and molecular breeding to improve saline-alkaline tolerance in peach.

## Background

Saline-alkaline stress adversely affects plant growth and development. Based on the Food and Agriculture Organization of the United Nations (FAO) research, at least 242 million hectares of saline-alkaline soils are distributed within the Eurasian Region [1].

High salt concentrations and high pH levels of saline-alkaline soil generate reactive oxygen species (ROS), repress photosynthesis, energy production, and lipid metabolism, and damage the plant cell membrane and intracellular components [2]. Upon saline-alkaline stress, a large cluster of gene expression is reprogrammed. Revealing the molecular mechanisms is helpful for saline-alkaline resistant plant breeding. In recent decades, several key genes such as *OsLOL5*, *Gshdz4*, and *SsMT2*, have been cloned and showed notable tolerance to saline-alkaline stress in transgenic plants [3–5]. Recently, with the rapid development of bioinformatics, transcriptome technology has become a powerful tool for elucidating the gene regulation of networks in many species [6, 7]. Using RNA-Seq technology, the effects of gene expression on saline-alkaline stress have been widely studied in alfalfa (*Medicago sativa* L.), flax (*Linum usitatissinum* L.), jujube (*Ziziphus jujuba* Mill.), black locust (*Robinia pseudoacacia* L.), and Chinese plum (*Prunus salicina* Lindl.), and thousands of differentially expressed genes have been detected [8–11].

Peach *(Prunus persica* L.) is an important deciduous fruit tree around the world. In China, the peach planting area is more than 700 thousand hectares, and the yield reaches more than one million tons per year [12]. The North China Plain and Sichuan Basin, which partly suffer from saline-alkaline stress, are the main productive areas of peach planting [13, 14]. Saline-alkaline stress inhabits peach plant growth and affects fruit development, finally causing a reduction of in yield. GF677 (*P. amygdalus* x *P. persica*), bred by the French Institut National de la Recherche Agronomique (INRA) in the 1960s, has higher salt and alkali tolerance than Maotao (*P. persica*) [15]. In 2017, Chen et al. identify more than one thousand genes differentially expressed between roots of GF677 and Maotao in response to saline-alkaline stresses [16]. Considering leaf chlorosis usually acts as an early symptom of this stress [17], we carried out cytological observations and comparative transcriptome analysis of GF677 and Maotao leaves under salt-alkali stress in the present study, to elucidate the molecular mechanism governing the salt-alkali tolerance of GF677. These findings will provide a theoretical basis for genetic improvement and breeding of peach under salt-alkali stress.

## Materials and methods

### Plant materials

The field experiment was performed in the Late White Peach Planting Base, Jianyang City, Sichuan Province (N30°30′50.97″, E104°26′35.21″). The pH of the test soil was 8.44 and the total salt content was 0.46% [15]. Two-years-old GF677 and Maotao plantlets were planted in the field with an inter-row spacing of 1 m × 4 m. As a control, two varieties were planted in a greenhouse with normal soil (pH = 7.05). Nine GF677 and nine Maotao plantlets were selected for further experiments. The leaves from each plantlet were grouped into two parts: one for RNA isolation and another for cytological and biochemical analysis.

### Ultrastructural observation

Each cultivar leaf sample was sliced into several sections, and soaked in 2% glutaraldehyde overnight at 4 °C. After rinsing with ddH_2_O, the samples were stained with 1% OsO_4_ for 2 h. The stained samples were further washed with ddH_2_O and dehydrated with acetone. The dehydrated samples were embedded in an epoxy resin and then sliced into ultrathin sections for ultrastructural observation as described by Jiang et al. [7].

### Physiological parameters measurement

#### Chlorophyll a and b

Leaves (0.2 g) of GF677 and Maotao were homogenized with 80% acetone. After centrifugation at 12,000 rpm for 10 min, the supernatant was transferred separately into a clean microcentrifuge tube. The chlorophyll a and chlorophyll b contents were measured with a spectrophotometer (TU-1810, Beijing, China) at 663 nm and 645 nm, respectively [7].

#### Iron content

Leaves (0.5 g) of GF677 and Maotao were washed with ddH_2_O. The total Fe concentrations were determined by inductively coupled plasma atomic emission spectroscopy (ICP-AES; Fisons ARL Accuris, Ecublens, Switzerland) [18].

#### Peroxidase activity

Leaves (0.5 g) of GF677 and Maotao were homogenized with liquid nitrogen, and peroxidase (POD) activity was determined according to the protocol of the Peroxidase Assay Kit (A084–3-1, Nanjing Jiancheng Bioengineering Institute, China).

#### RNA extraction and transcriptome sequencing

For each cultivar, nine plantlets were randomly selected for RNA extraction. Total RNA of each plantlet was extracted according to the protocol of the RNAprep Pure Plant Plus Kit (Tiangen Biotech Co., Ltd., Beijing, China). RNA from three plantlets was equally pooled for cDNA library construction and transcriptome sequencing. For library preparation, mRNA was first extracted using oligo (dT) and then broken into 200 bp segments by adding fragmentation buffer. The first-stranded cDNA was synthesized by random hexamers, and the second-stranded cDNA was obtained by adding dNTPs and DNA polymerase I. The purified second strand cDNA was finally amplified using PCR. The libraries were sequenced on an Illumina HiSeq™ 4000 platform (Majorbio, Shanghai, China), and all raw reads were submitted to NCBI with the accession number GSE100180.

#### Sequence data assembly

Clean reads were obtained by filtering adapters, poly-N and low-quality reads from the raw data. The reference genome of peach (version 2.1) was downloaded from the website https://phytozome.jgi.doe.gov/pz/portal.html#!info?alias=Org_Ppersica. An index of the reference genome was built using Bowtie v2.0.6, and all clean reads were then mapped to the reference genome using TopHat2 (v. 2.0.9). The transcript abundance of mapped genes was normalized by the fragments per kilobase of exon per million fragments mapped using Cufflinks (v. 2.1.1).

#### Differentially expressed gene (DEG) identification and functional annotation

Bioconductor package edgeR (v. 3.0.8) was used to identify DEGs. FDR ≤ 0.01 and |log2 ratio| ≥ 1 were the criteria for recognizing the significance of the gene expression difference. For visual display the DEGs by MapMan (version 3.6.0RC1) [19], all gene IDs were transformed to version 1.0 format. Gene ontology (GO) analysis of the DEGs was implemented using the GOseq package.

#### qRT-PCR validation

Total RNA from the leaves of the two cultivars was extracted as previously described. Total RNA (1 μg) was reverse transcribed to cDNA according to the RT reagent kit (Takara Bio Inc., Japan). The reaction mixture was used as follows: 10 μl SYBR Green 127 Premix Ex Taq, 10 μl cDNA, 0.5 μM forward primer, 0.5 μM reverse primer. The amplification was performed on a Bio-Rad CFX96 real-time system. The *UBQ10* gene was used for the normalization of the tested gene [20]. The primers used in the present research are shown in Table S1.

#### Phylogenetic analysis

The AtR2R3-MYB and AtbHLH protein sequences were downloaded from previous research [7]. The AtWRKY protein sequences were obtained from online databases (http://www.arabidopsis.org/). Other species related sequences were obtained from NCBI (Table S2). The construction of the phylogenetic tree was performed by the online software Multiple Sequence Alignment and iTOL [7].

### Statistical analysis

All analyses in the present research were carried out at least in triplicate. Based on the t-test, GraphPad Prism 5 was used for statistical analysis. Double stars showed a 0.01 significant difference.

## Results

### Cytological and physiological features of two peach cultivars in tolerating saline-alkaline stress

**Fig. 1:**
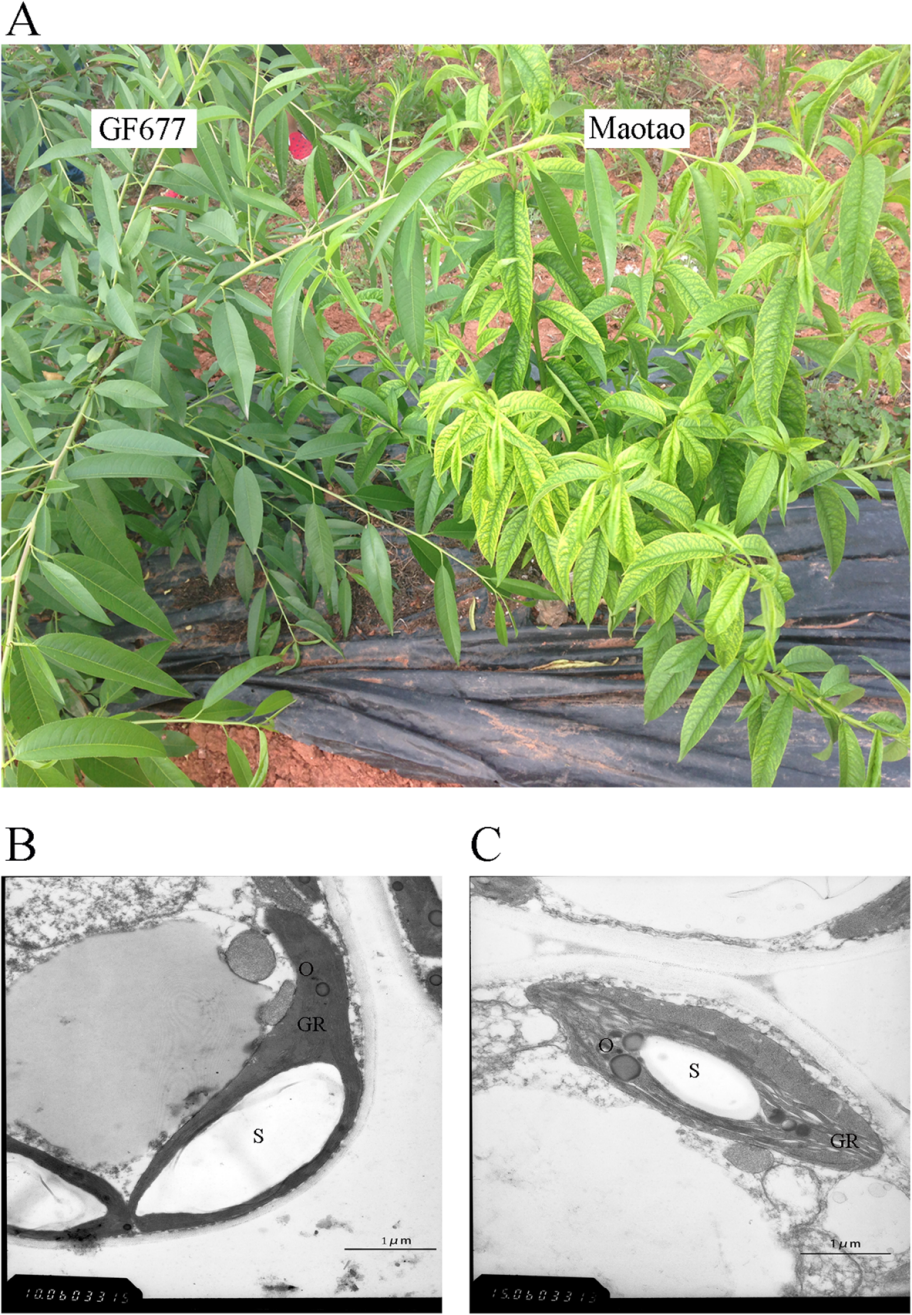
Phenotypes (**a**) and chloroplast ultrastructures (**b**-**c**) of GF677 and Maotao under salt-alkali stress. **b** Chloroplast ultrastructures of GF677. **c** Chloroplast ultrastructures of Maotao. The scale bar was shown 1 μm. GR: granum; S: starch grain; O: osmiophile globule

There was no difference between GF677 and Maotao in normal soil (Fig. S1). However, the phenotypes of the two varieties differed upon saline alkaline field planting (Fig. 1a). In contrast to GF677, the leaves of Maotao were clear yellowish. The TEM analysis of leaves revealed a large structural change between the two cultivars. The chloroplast of GF677 had a compact granum structure, but the structure of the granum was dispersive in the Maotao chloroplast (Fig. 1b-c). Further chlorophyll determination showed that the contents of chlorophyll a or b in Maotao were only 22.3% or 15.9% in comparison with GF677. The POD enzyme activity and Fe content decreased by 50% in Maotao (Table 1).

**Table 1:** Physiological parameters of GF677 and Maotao under saline-alkaline stress

	GF677	Maotao
**POD(U/(g.min))**	723.76^a^	368.60
**Chl a(mg/g)**	1.38^a^	0.29
**Chl b(mg/g)**	0.82^a^	0.13
**Fe(%)**	0.49^a^	0.25

Note: ^a^represent 0.01 significant difference

### Illumina sequence and DEG identification

**Fig. 2:**
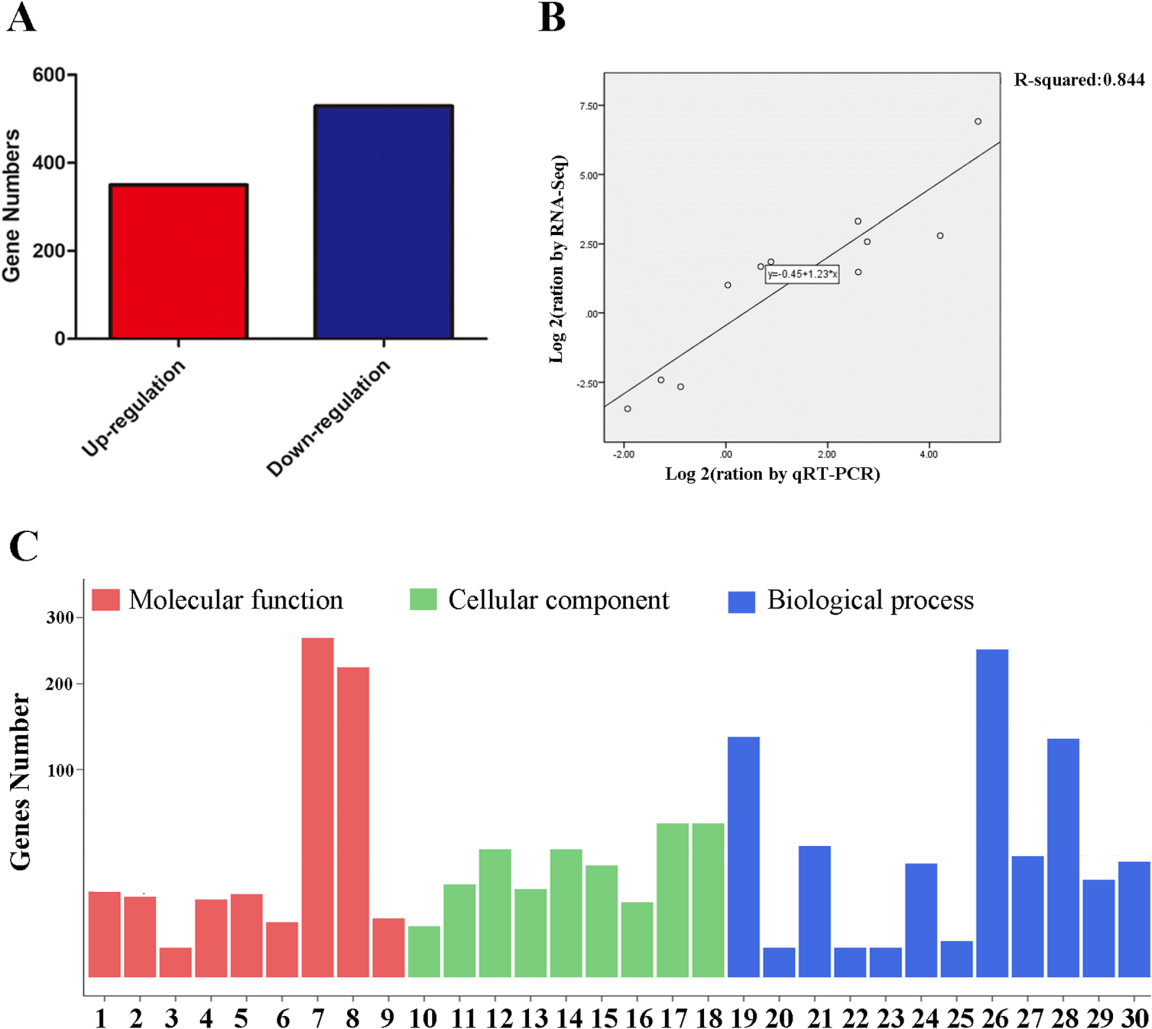
Identification (**a**), verification (**b**) and classification (**c**) of DEGs. **a** DEGs identification. The red or blue column represented up-regulation or down-regulation, respectively. **b** DEGs validation. 11 DEGs were randomly selected for validation by qRT-PCR. **c** DEGs classification. All DESs were classified by Gene Ontology (GO). 1: transporter activity; 2: structural molecule activity; 3: signal transducer activity; 4: nucleic acid binding transcription factor activity; 5: molecular function regulator; 6: electron carrier activity; 7: catalytic activity; 8: binding; 9:antioxidant activity; 10: supra molecular fiber; 11: organelle part; 12: organelle; 13:membrane part; 14: membrane; 15: macromolecular complex; 16: extracellular region;17: cell part; 18: cell; 19: single-organism process; 20: signaling; 21: response to stimulus; 22: reproductive process; 23: reproduction; 24: regulation of biological process; 25: multi-organism process; 26: metabolic process; 27: localization; 28:cellular process; 29: cellular component organization or biogenesis; 30: biological regulation

A total of 29,006,496 and 31,874,666 clean reads were obtained in Maotao and GF677, respectively. The Q20 was more than 96.00% and the Q30 was more than 90.00%, suggesting the high quality of the clean reads. Of all clean reads, 83.68% ~ 90.43% were mapped to the reference genome (Table 2). To identify the gene response to saline-alkaline stress, the criteria of FDR ≤ 0.01 and |log2 ratio| ≥ 1 were used as thresholds. Among the DEGs, 294 and 587 were significantly up- and downregulated GF677 versus Maotao (Fig. 2a). In this study, the upregulation represented genes highly expressed in GF677 and downregulated showed genes highly expressed in Maotao. To validate the expression of DEGs, 11 genes involved in photosynthesis (*ppa007547*, *ppa012123*), carbohydrate metabolism (*ppa007458*), antioxidation (*ppa027053*, *ppa011202*), metal transporter (*ppa003097*), stress (*ppa008441*), polyamine biosynthesis (*ppa007732*), and transcription factor (*ppa007708*, *ppa010846*, *ppa016095*) were selected for the validation of DEGs, and the results showed that their expression pattern was highly related to the DEG data (Fig. 2b). To functionally characterize the DEGs, GO analysis was performed. A total of 30 GO terms were grouped, including several stress-related terms, such as “transporter activity”, “antioxidant activity”, “response to stimulus” (Fig. 2c). In the “molecular function” part, nine groups were obtained, and the “catalytic activity” was the most abundant group. In the “cell component” part, nine groups were obtained, and the “cell parts” was the most abundant group. In the “biological process” part, 12 groups were obtained, and the “metabolic process” was the most abundant group (Fig. 2c).

**Table 2:** Summary of transcriptome sequencing data from leaves of GF677 (GY) and Maotao (MY) under saline-alkaline stress

Sample	Raw data (GB)	Clean reads	Clean data ratio (%)	Clean data Q20 (%)	Clean data Q30 (%)	Mapped genome (%)	Expressed gene
**GY_rep1**	4.24	30,175,284	99.33	96.55	91.59	84.02%	21,234
**GY_rep2**	4.19	29,817,828	99.44	96.47	91.40	89.43%	22,238
**GY_rep3**	4.15	29,488,462	99.27	96.66	91.91	83.68%	21,653
**MY_rep1**	4.48	31,874,666	99.48	96.12	90.62	89.36%	22,365
**MY_rep2**	4.08	29,006,494	99.31	96.62	91.71	89.66%	22,025
**MY_rep3**	4.28	30,471,936	99.37	96.45	91.53	90.43%	20,950

### Overview of metabolism processes by MapMan

**Fig. 3:**
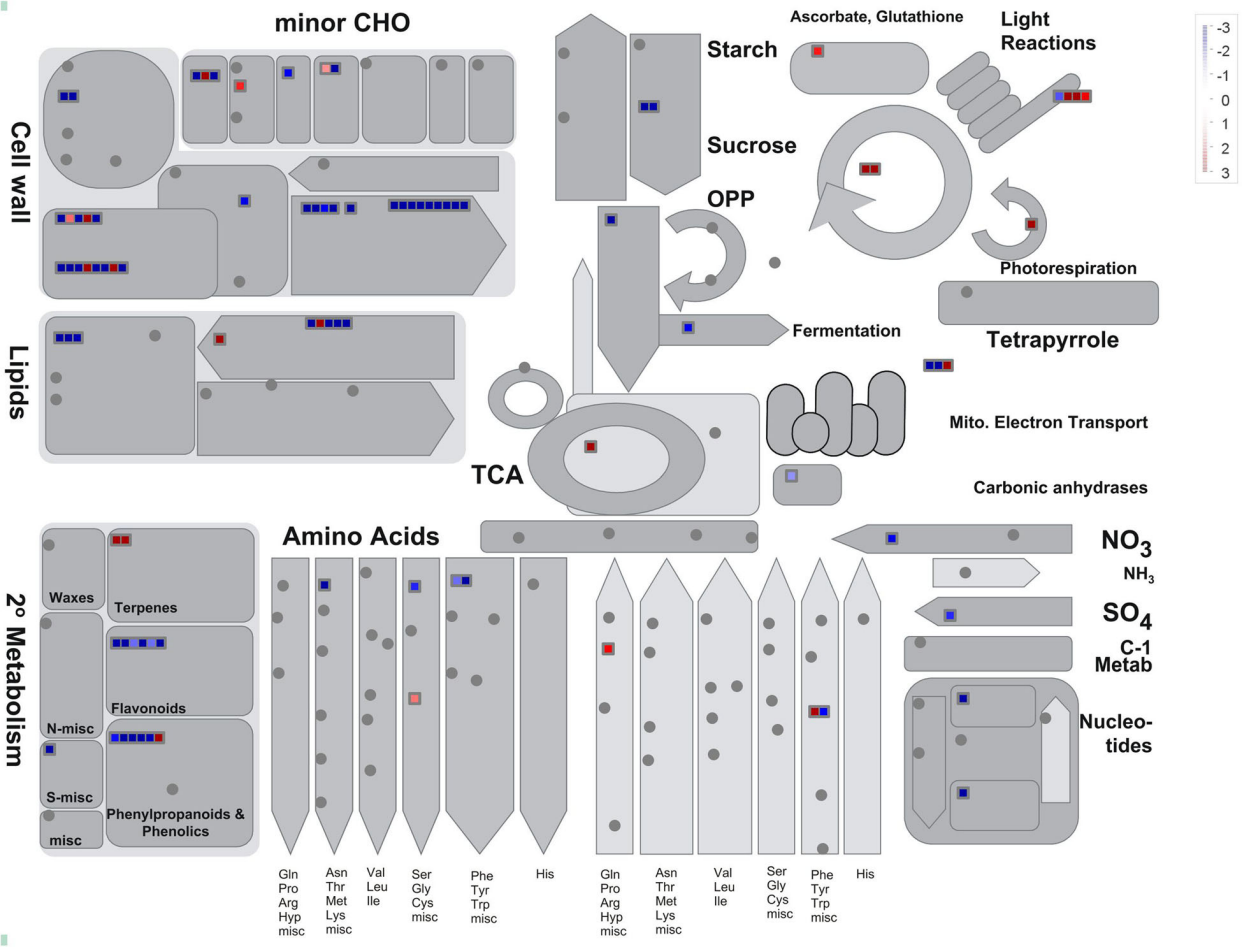
MAPMAN visualization of DEGs related to metabolic pathways. **a** The log2 value of significant DEGs was imported and visualized in MapMan software. Red or blue color indicated up-regulation or down-regulation, respectively. The scale bar was shown from − 3 to 3

To provide an overview of the DEGs at the metabolic process level, the MapMan tool was used. It was clearly shown that photosynthesis was upregulated in GF677, while cell wall degradation, secondary metabolism, and starch degradation were activated in Maotao (Fig. 3; Table S3).

### Category and verification of genes related to antioxidation, iron metabolism, transcription factor

#### Antioxidation

**Fig. 4:**
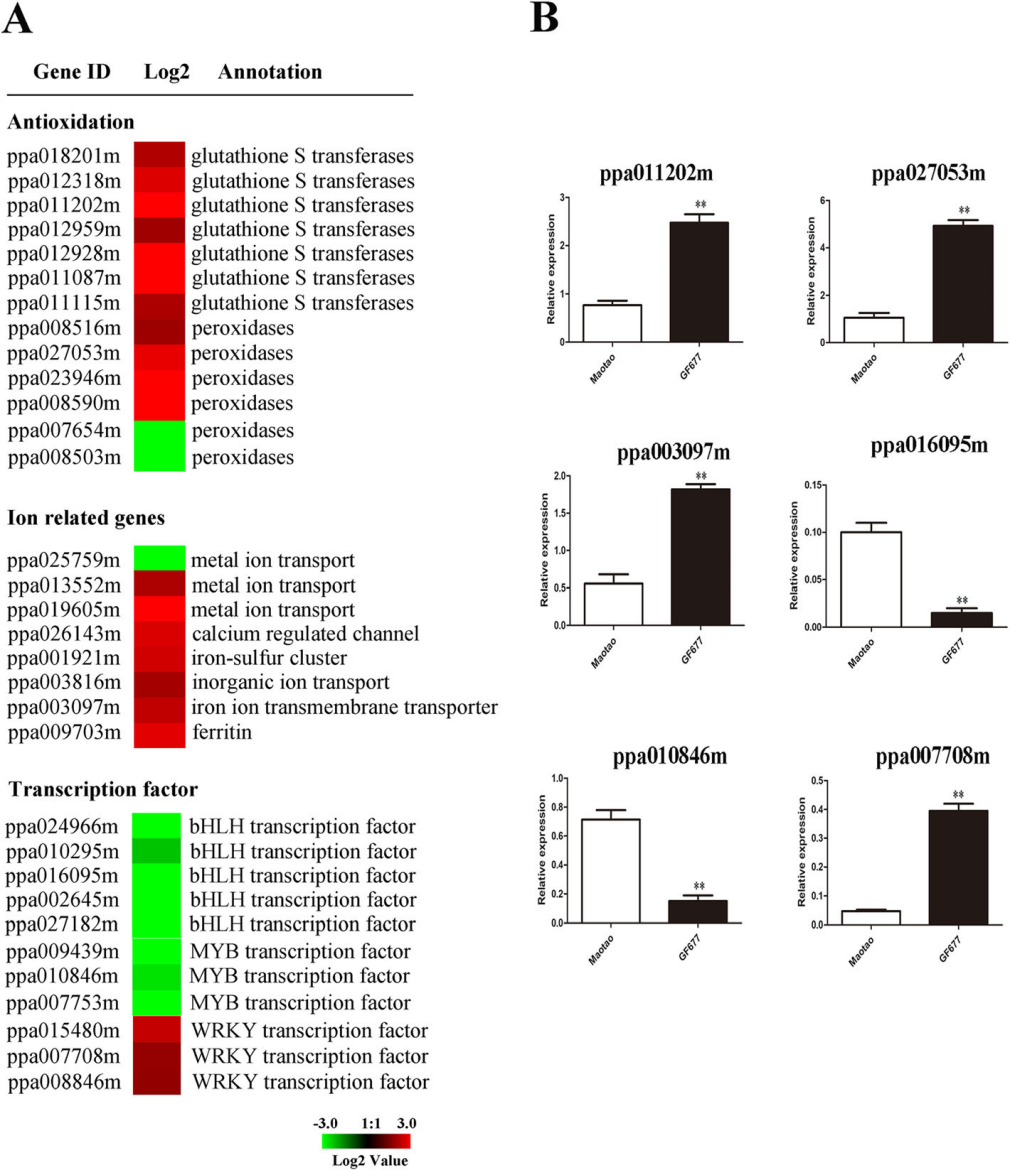
DEGs related to antioxidation, ion, transcription factor. The left showed heat map of DEGs (**a**) and the right showed qRT-PCR verification (**b**). DEG values displayed as heat map. Colors bar represented expression levels of each gene which were either up-regulated (red) or down-regulated (green). Error bars for qRT-PCR showed the standard deviation of three replicates. ** represented 0.01significant differences. Correlation analysis of Log 2 change values obtained from RNA-Seq and qRT-PCR

Seven genes encoding glutathione S-transferase (GST) and six genes encoding POD were detected in DEGs (Fig. 4a). Of these genes, all seven *GST* genes and four *POD* genes were highly upregulated in GF677. *ppa011202m* encoding a GST and *ppa027053m* encoding a POD were further confirmed by qRT-PCR (Fig. 4b).

#### Iron metabolism

Eight iron-related genes were detected in the present research. Seven of them were upregulated, including two genes (*ppa013552m*, *ppa019605m*) encoding a metal ion transporter, one gene (*ppa026143m*) encoding calcium-regulated channel protein, one gene (*ppa001921m*) encoding an iron-sulfur cluster, one gene (*ppa003816m*) encoding an inorganic ion transport, one gene (*ppa003097m*) encoding an iron ion transmembrane transporter, and one gene (*ppa009703m*) encoding ferritin (Fig. 4a). Of these genes, *ppa003097m* was further confirmed by qRT-PCR (Fig. 4b).

#### Transcription factor

Five genes encoding bHLH, three genes encoding MYB, and three genes encoding WRKY were detected in this study. Interestingly, the expression of bHLH and MYB downregulated, in contrast to the upregulation of WRKY (Fig. 4a). Furthermore, *ppa016095m* encoding a bHLH, *ppa010846m* encoding an MYB, and *ppa007708m* encoding a WRKY were confirmed by qRT-PCR (Fig. 4b).

**Fig. 5:**
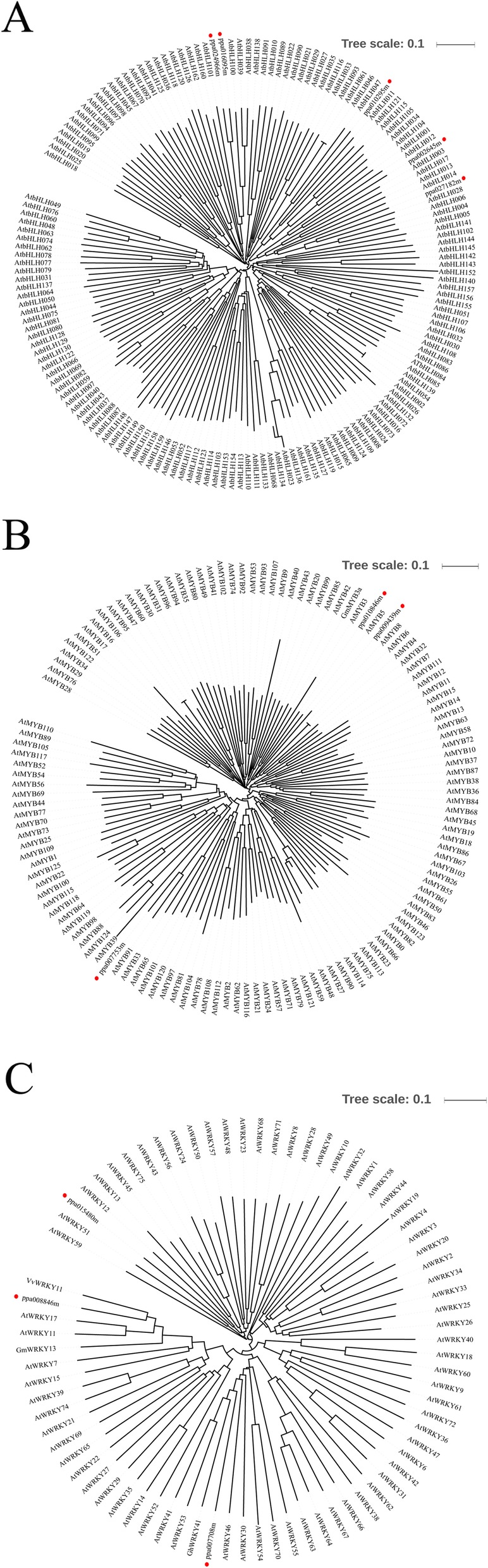
Phylogenetic relationships of bHLH (**a**), R2R3-MYB (**b**), WRKY (**c**) related DEGs with Arabidopsis and other species homolog. The trees were constructed by neighboring-joining phylogeny test, and 1000 bootstrap replicates. The accession numbers for the genes are provided in Table S2. The blue circle represented ion or salt stress-related TFs in other species. The red circle represented DEGs identified in this study.

We further constructed phylogenetic trees by integrating Arabidopsis homologues and other species functional verification genes (Fig. 5). The results showed that for each phylogenetic tree, the peach genes were scattered into different clades (Fig. 5a-c).

## Discussion

As a serious abiotic stress, saline-alkaline stress affects plant growth and reduces yield. In peach, comparative transcriptome analysis of roots of GF677 and Maotao suggests osmotic pressure increase and redox balance are crucial for GF677 response to saline-alkaline stresses [16]. Leaf chlorosis usually acts as an early symptom of this stress [10, 11]. Our previous research showed that the photosynthetic rate was significantly higher in the leaves of GF677 than in Maotao [21]. In the present research, TEM analysis revealed that the chloroplast granum structure was more intact and the content of chlorophyll a/b was higher, in GF677, suggesting that GF677 maintains higher photosynthesis efficiency under saline-alkaline stress (Fig. 1b-c, Table 1). Iron is an important ion that plays a key role in sustaining the activity of photosynthesis-related enzymes [22, 23]. In apple, Wang et al. [24] reported that Fe deficiency induces significant downregulation of genes involved in photosynthesis. In the present study, we show that iron content is higher in GF677, which is consistent with the upregulation of several iron and photosynthesis-related genes.

Saline-alkaline stress causes the accumulation of ROS, which severely damages such cell components as DNA, lipids, proteins, and sugars [25]. Detoxification of ROS is the most important way in plants to weaken oxidative harmful stress [26]. Of the ROS scavenging enzyme family, POD and GST are the key members [27, 28]. Previous studies have shown that these proteins usually play a synergetic role in scavenging ROS. In cotton, Li et al. identified that the POD and GST proteins are upregulated upon salt treatment, and both these upregulation effects are further verified by qRT-PCR [29]. In the present research, notably, we found that POD activity was higher in GF677 (Table 1). This result is in accordance with the upregulation of most POD-related genes. Additionally, we also found that all seven GST genes were upregulated in GF677 (Fig. 4). Considering that the transcript level of a gene can largely reflect its translation state generally, we speculate that GST and POD have a synergistic role in scavenging ROS in GF677, which is consistent with the previous study [16].

TFs are key regulators of gene expression and have a variety of important functions in the plant response to abiotic stress [30, 31]. The identification of TFs involved in saline-alkaline stress is crucial to reveal the innate molecular mechanisms. In the present research, 11 genes related to bHLH, MYB, and WRKY TFs were detected (Fig. 4). As the most extensive TF class in eukaryotes, bHLH is not only universally involved in plant growth and metabolism but also plays an important role in plant response to stress [32, 33]. Fan et al. [34] revealed that Ib subgroup bHLH genes (*AtbHLH38*, *AtbHLH39*, *AtbHLH100*, and *AtbHLH101*) can bind the promoter of *SKB1* and negatively regulate the accumulation of iron. In the present study, two-bHLH encoding genes (*ppa024966m* and *ppa010295m*), together with AtbHLH38, AtbHLH39, AtbHLH100, and AtbHLH101, were grouped into a subgroup by phylogenetic analysis (Fig. 5a). This result suggests ppa024966m and ppa010295m may have a similar role as Ib subgroup bHLHs, which is further confirmed by the higher content of Fe in GF677. Babitha et al. [35] showed that overexpression of AtbHLH17 enhances tolerance to NaCl, mannitol and oxidative stress in transgenic lines. Notably, ppa0271826m and ppa002645m show high homology to AtbHLH17, suggesting their putative function in the regulation of saline alkaline stress. MYB is one of the largest TF families in plants [36]. The R2R3-MYB subfamily plays a key role in diverse biological processes, especially in response to various stresses [37, 38]. In soybean, overexpression of *GmMYB3a* negatively regulates saline-alkaline stress-related genes [39]. In Arabidopsis, Cui et al. [40] showed that overexpression of *AtMYB20* can enhance salt tolerance by negatively regulating type 2C serine/threonine protein phosphatases. In the present study, the phylogenetic tree shows that ppa010846m and ppa009439m are homologous to AtMYB20 and GmMYB3a, indicating a putative role in the regulation of saline-alkaline stress (Fig. 5b). WRKY is a class of TFs unique to plants that are mainly involved in development and stress responses [41, 42]. In Arabidopsis, overexpression of *AtWRKY46*, *GmWRKY13* or *VvWRKY11* can both positively regulate salt and drought stress tolerance [43–45]. In *Nicotiana benthamiana*, overexpression of *GhWRKY41* confers transgenic plant salt and drought stress tolerance [46]. In the present study, phylogenetic analysis shows that ppa008846m is highly similar to GmWRKY13 and VvWRKY11, while ppa007708m together with GhWRKY41 and AtWRKY46 are grouped into same subgroup (Fig. 5c), suggesting that it exhibits a similar function in the regulation of saline-alkaline stress.

## Conclusions

In the present study, the biochemical, cytological and transcriptome differences between GF677 and Maotao were systematically analysed under saline-alkaline stress. The structure of chloroplast granum was intact in GF677, but dispersed in Maotao. Functional analysis of 881 DEGs showed that photosynthesis was activated, whereas cell wall degradation, secondary metabolism, and starch degradation were repressed in GF677. Based on the phylogenetic tree integration of other species homologues, several stress-related TFs were further functionally discussed. We speculate that the integrity of chloroplast structure and the activation of photosynthesis as well as stress-related genes, are crucial for saline-alkaline resistance in GF677. The results described in this report provide a theoretical basis for cloning saline-alkaline tolerance genes and molecular breeding for improving saline-alkaline tolerance in peach.

## Supplementary information

**Additional file 1: Table S1.** Primers used in this paper.

**Additional file 2: Table S2.** Gene accession numbers and amino acid sequences used in this paper.

**Additional file 3: Table S3.** Gene expression pattern for primary and secondary metabolisms.

**Additional file 4: Figure S1.** Phenotypes (A) and chloroplast ultrastructures (B-C) of GF677 and Maotao in normal soil. **(**B) Chloroplast ultrastructures of GF677. (C) Chloroplast ultrastructures of Maotao. The scale bar was shown 1 μm. GR: granum; S: starch grain; O: osmiophile globule

## Data Availability

We have provided detailed information about the materials and methods in our manuscript.

## Abbreviations

DEG: Differentially expressed gene
FAO: Food and Agriculture Organization of the United Nations
GO: Gene ontology
GST: Glutathione S-transferase
INRA: Institut Nationaldela Recherche Agronomique
POD: Peroxidase
qRT-PCR: Quantitative real-time PCR
ROS: Reactive oxygen species
TEM: Transmission electron microscopy
