# Supplementary material for: Comparative transcriptome analysis reveals gene expression differences between two peach cultivars under saline-alkaline stress
Source: Hereditas. 2020 Mar 31;157:9. doi: 10.1186/s41065-020-00122-4 (PMC7110815; doi:10.1186/s41065-020-00122-4)
Supplement: Supplementary file 1 — Additional file 1: Table S1. Primers used in this paper. [file 41065_2020_122_MOESM1_ESM.docx]

Table S1. Primers used in this paper.

| Gene Name | Primer sequence | Annotation |
| --- | --- | --- |
| *ppa007547m* | Forward: GAAAAGAAATGCTTATGC  Reverse: CAGTGAGCTACTTGTGGG | NADPH dehydrogenase |
| *ppa012123m* | Forward: CTTGGAATTTGAGTTGGA  Reverse: GAAAGAGTTGGGGTTAGC | Ribulose-bisphosphate carboxylase small chain |
| *ppa007732m* | Forward: ACTCCAGCAACTATCTTC  Reverse: CTTCCTCTTCTTCATCTC | S-adenosylmethionine decarboxylase proenzyme |
| *ppa008441m* | Forward: GAACTACGCTTCTGGTCA  Reverse: TCCTCCTCGTCTTCCTCT | Heat stress |
| *ppa007708m* | Forward: CGAGAAGCAGAAAAAC  Reverse: TAGCCATCCATCAAAG | WRKY transcription factor |
| *ppa010846m* | Forward: CAAATAACCACCGACC  Reverse: TTCATCCTCCAAGCAA | MYB transcription factor |
| *ppa016095m* | Forward: AGAAGCGAATAAAAAG  Reverse: GATGACTGGAAGGAAG | bHLH transcription factor |
| *ppa003097m* | Forward: TACCATCATCCAAAGC  Reverse: CAACGACAGAACATCC | Iron ion transmembrane transporter |
| *ppa027053m* | Forward: CGACCACAACTACTTC  Reverse: ATCTCTCCATCACTGC | Peroxidases |
| *ppa011202m* | Forward: TGCTCTCATCACATTC  Reverse: CTATTCCACACCAAAC | Glutathione S transferases |
| *ppa007458m* | Forward: TGACTGTCCCCCTCACAC  Reverse: GGCTTCTTCAATCTCCTT | Sugar alcohols |
| *UBQ10* | Forward: AGGCTAAGATCCAAGACAAAGAG  Reverse: CCACGAAGACGAAGCACTAAG | Ubiquitin 10 |
