# Supplementary material for: Comparative transcriptome analysis reveals gene expression differences between two peach cultivars under saline-alkaline stress
Source: Hereditas. 2020 Mar 31;157:9. doi: 10.1186/s41065-020-00122-4 (PMC7110815; doi:10.1186/s41065-020-00122-4)
Supplement: Supplementary file 2 — Additional file 2: Table S2. Gene accession numbers and amino acid sequences used in this paper. [file 41065_2020_122_MOESM2_ESM.docx]

Tabel S2 Gene accession numbers and amino acid sequences used in this paper.

**bHLH**

>AtbHLH003

MGQKFWENQEDRAMVESTIGSEACDFFISTASASNTALSKLVSPPSDSNLQQGLRHVVEGSDWDYALFWLASNVNSSDGCVLIWGDGHCRVKKGASGEDYSQQDEIKRRVLRKLHLSFVGSDEDHRLVKSGALTDLDMFYLASLYFSFRCDTNKYGPAGTYVSGKPLWAADLPSCLSYYRVRSFLARSAGFQTVLSVPVNSGVVELGSLRHIPEDKSVIEMVKSVFGGSDFVQAKEAPKIFGRQLSLGGAKPRSMSINFSPKTEDDTGFSLESYEVQAIGGSNQVYGYEQGKDETLYLTDEQKPRKRGRKPANGREEALNHVEAERQRREKLNQRFYALRAVVPNISKMDKASLLADAITYITDMQKKIRVYETEKQIMKRRESNQITPAEVDYQQRHDDAVVRLSCPLETHPVSKVIQTLRENEVMPHDSNVAITEEGVVHTFTLRPQGGCTAEQLKDKLLASLSQ

>AtbHLH004

MSPTNVQVTDYHLNQSKTDTTNLWSTDDDASVMEAFIGGGSDHSSLFPPLPPPPLPQVNEDNLQQRLQALIEGANENWTYAVFWQSSHGFAGEDNNNNNTVLLGWGDGYYKGEEEKSRKKKSNPASAAEQEHRKRVIRELNSLISGGVGGGDEAGDEEVTDTEWFFLVSMTQSFVKGTGLPGQAFSNSDTIWLSGSNALAGSSCERARQGQIYGLQTMVCVATENGVVELGSSEIIHQSSDLVDKVDTFFNFNNGGGEFGSWAFNLNPDQGENDPGLWISEPNGVDSGLVAAPVMNNGGNDSTSNSDSQPISKLCNGSSVENPNPKVLKSCEMVNFKNGIENGQEEDSSNKKRSPVSNNEEGMLSFTSVLPCDSNHSDLEASVAKEAESNRVVVEPEKKPRKRGRKPANGREEPLNHVEAERQRREKLNQRFYSLRAVVPNVSKMDKASLLGDAISYISELKSKLQKAESDKEELQKQIDVMNKEAGNAKSSVKDRKCLNQESSVLIEMEVDVKIIGWDAMIRIQCSKRNHPGAKFMEALKELDLEVNHASLSVVNDLMIQQATVKMGNQFFTQDQLKVALTEKVGECP

>AtbHLH005

MNGTTSSINFLTSDDDASAAAMEAFIGTNHHSSLFPPPPQQPPQPQFNEDTLQQRLQALIESAGENWTYAIFWQISHDFDSSTGDNTVILGWGDGYYKGEEDKEKKKNNTNTAEQEHRKRVIRELNSLISGGIGVSDESNDEEVTDTEWFFLVSMTQSFVNGVGLPGESFLNSRVIWLSGSGALTGSGCERAGQGQIYGLKTMVCIATQNGVVELGSSEVISQSSDLMHKVNNLFNFNNGGGNNGVEASSWGFNLNPDQGENDPALWISEPTNTGIESPARVNNGNNSNSNSKSDSHQISKLEKNDISSVENQNRQSSCLVEKDLTFQGGLLKSNETLSFCGNESSKKRTSVSKGSNNDEGMLSFSTVVRSAANDSDHSDLEASVVKEAIVVEPPEKKPRKRGRKPANGREEPLNHVEAERQRREKLNQRFYSLRAVVPNVSKMDKASLLGDAISYINELKSKLQQAESDKEEIQKKLDGMSKEGNNGKGCGSRAKERKSSNQDSTASSIEMEIDVKIIGWDVMIRVQCGKKDHPGARFMEALKELDLEVNHASLSVVNDLMIQQATVKMGSQFFNHDQLKVALMTKVGENY

>AtbHLH007

MANNNNIPHDSISDPSPTDDFFEQILGLSNFSGSSGSGLSGIGGVGPPPMMLQLGSGNEGNHNHMGAIGGGGPVGFHNQMFPLGLSLDQGKGHGFLKPDETGKRFQDDVLDNRCSSMKPIFHGQPMSQPAPPMPHQQSTIRPRVRARRGQATDPHSIAERLRRERIAERIRSLQELVPTVNKTDRAAMIDEIVDYVKFLRLQVKVLSMSRLGGAGAVAPLVTEMPLSSSVEDETQAVWEKWSNDGTERQVAKLMEENVGAAMQLLQSKALCIMPISLAMAIYHSQPPDTSSSIVKPEMNPPPUIFVHPTVPSUULTFCSVSHYUTFVTHERUVKRHWRWNLSRICAVKKUNGICQKKEKALACLASIYHFDESNSFLFKDFSVIFRTKGFEGSA

>AtbHLH009

MLLLRSDMEHQGWSFEENYSLSTNRRSIRPQDELVELLWRDGQVVLQSQTHREQTQTQKQDHHEEALRSSTFLEDQETVSWIQYPPDEDPFEPDDFSSHFFSTMDPLQRPTSETVKPKSSPEPPQVMVKPKACPDPPPQVMPPPKFRLTNSSSGIRETEMEQYSVTTVGPSHCGSNPSQNDLDVSMSHDRSKNIEEKLNPNASSSSGGSSGCSFGKDIKEMASGRCITTDRKRKRINHTDESVSLSDAIGNKSNQRSGSNRRSRAAEVHNLSERRRRDRINERMKALQELIPHCSKTDKASILDEAIDYLKSLQLQLQVMWMGSGMAAAAASAPMMFPGVQPQQFIRQIQSPVQLPRFPVMDQSAIQNNPGLVCQNPVQNQIISDRFARYIGGFPHMQAATQMQPMEMLRFSSPAGQQSQQPSSVPTKTTDGSRLDH

>AtbHLH010

MEEERESLYEEMGCFDPNTPAEVTVESSFSQAEPPPPPPQVLVAGSTSNSNCSVEVEELSEFHLSPQDCPQASSTPLQFHINPPPPPPPPCDQLHNNLIHQMASHQQQHSNWDNGYQDFVNLGPNSATTPDLLSLLHLPRCSLPPNHHPSSMLPTSFSDIMSSSSAAAVMYDPLFHLNFPMQPRDQNQLRNGSCLLGVEDQIQMDANGGMNVLYFEGANNNNGGFENEILEFNNGVTRKGRGSRKSRTSPTERERRVHFNDRFFDLKNLIPNPTKIDRASIVGEAIDYIKELLRTIEEFKMLVEKKRCGRFRSKKRARVGEGGGGEDQEEEEDTVNYKPQSEVDQSCFNKNNNNSLRCSWLKRKSKVTEVDVRIIDDEVTIKLVQKKKINCLLFTTKVLDQLQLDLHHVAGGQIGEHYSFLFNTKICEGSCVYASGIADTLMEVVEKQYMEAVPSNGY

>AtbHLH011

MAVSCLFIVSSNYRGAEMVVEVKKEAVCSQKAEREKLRRDKLKEQFLELGNALDPNRPKSDKASVLTDTIQMLKDVMNQVDRLKAEYETLSQESRELIQEKSELREEKATLKSDIEILNAQYQHRIKTMVPWVPHYSYHIPFVAITQGQSSFIPYSASVNPLTEQQASVQQHSSSSADASMKQDSKIKPLDLDLMMNSNHSGQGNDQKDDVRLKLELKIHASSLAQQDVSGKEKKVSLTTTASSSNSYSLSQAVQDSSPGTVNDMLKP

>AtbHLH012

MSLTMADGVEAAAGRSKRQNSLLRKQLALAVRSVQWSYAIFWSSSLTQPGVLEWGEGCYNGDMKKRKKSYESHYKYGLQKSKELRKLYLSMLEGDSGTTVSTTHDNLNDDDDNCHSTSMMLSPDDLSDEEWYYLVSMSYVFSPSQCLPGRASATGETIWLCNAQYAENKLFSRSLLARSASIQTVVCFPYLGGVIELGVTELISEDHNLLRNIKSCLMEISAHQDNDDEKKMEIKISEEKHQLPLGISDEDLHYKRTISTVLNYSADRSGKNDKNIRHRQPNIVTSEPGSSFLRWKQCEQQVSGFVQKKKSQNVLRKILHDVPLMHTKRMFPSQNSGLNQDDPSDRRKENEKFSVLRTMVPTVNEVDKESILNNTIKYLQELEARVEELESCMGSVNFVE

RQRKTTENLNDSVLIEETSGNYDDSTKIDDNSGETEQVTVFRDKTHLRVKLKETEVVIEVRCSYRDYIVADIMETLSNLHMDAFSVRSHTLNKFLTLNLKAKFRGAAVASVGMIKRELRRVIDFREPICDVPLSLHQVFRVFVCKVCQSLVGIFDNVVSSSSTKPRSILIHNSWAICIFH

>AtbHLH014

MYNLTFSPSLSSSLLSFTQQTPAAIVSSSPPDLVLQQKLRFVVETSPDRWAYVIFWQKMFDDQSDRSYLVWVDGHFCGNKNNNSQENYTTNSIECELMMDGGDDLELFYAASFYGEDRSPRKEVSDESLVWLTGPDELRFSNYERAKEAGFHGVHTLVSIPINNGIIELGSSESIIQNRNFINRVKSIFGSGKTTKHTNQTGSYPKPAVSDHSKSGNQQFGSERKRRRKLETTRVAAATKEKHHPAVLSHVEAEKQRREKLNHRFYALRAIVPKVSRMDKASLLSDAVSYIESLKSKIDDLETEIKKMKMTETDKLDNSSSNTSPSSVEYQVNQKPSKSNRGSDLEVQVKIVGEEAIIRVQTENVNHPTSALMSALMEMDCRVQHANASRLSQVMVQDVVVLVPEGLRSEDRLRTTLVRTLSL

>AtbHLH018

MATAMNVFSTKWSSELDIEEYSIIHQFHMNSLVGDVPQSLSSLDDTTTCYNLDASCNKSLVEERPSKILKTTHISPNLHPFSSSNPPPPKHQPSSRILSFEKTGLHVMNHNSPNLIFSPKDEEIGLPEHKKAELIIRGTKRAQSLTRSQSNAQDHILAERKRREKLTQRFVALSALIPGLKKMDKASVLGDAIKHIKYLQESVKEYEEQKKEKTMESVVLVKKSSLVLDENHQPSSSSSSDGNRNSSSSNLPEIEVRVSGKDVLIKILCEKQKGNVIKIMGEIEKLGLSITNSNVLPFGPTFDISIIAQKNNNFDMKIEDVVKNLSFGLSKLT

>AtbHLH020

MDDSSFMDLMIDTDEYLIDDWESDFPICGETNTNPGSESGSGTGFELLAERPTKQMKTNNNMNSTSSSPSSSSSSGSRTSQVISFGSPDTKTNPVETSLNFSNQVSMDQKVGSKRKDCVNNGGRREPHLLKEHVLAERKRRQKLNERLIALSALLPGLKKTDKATVLEDAIKHLKQLQERVKKLEEERVVTKKMDQSIILVKRSQVYLDDDSSSYSSTCSAASPLSSSSDEVSIFKQTMPMIEARVSDRDLLIRVHCEKNKGCMIKILSSLEKFRLEVVNSFTLPFGNSTLVITILTKMDNKFSRPVEEVVKNIRVALAE

>AtbHLH023

MTWKPKMLILSHDLISPEKYIMGEDDIVELLGKSSQVVTSSQTQTPSCDPPLILRGSGSGDGEGNGPLPQPPPPLYHQQSLFIQEDEMASWLHQPNRQDYLYSQLLYSGVASTHPQSLASLEPPPPPRAQYILAADRPTGHILAERRAENFMNISRQRGNIFLGGVEAVPSNSTLLSSATESIPATHGTESRATVTGGVSRTFAVPGLGPRGKAVAIETAGTQSWGLCKAETEPVQRQPATETDITDERKRKTREETNVENQGTEEARDSTSSKRSRAAIMHKLSERVLMLPLEFQDSEFE

>AtbHLH025

MSILSTRWFSEQEIEENSIIQQFHMNSIVGEVQEAQYIFPHSFTTNNDPSYDDLIEMKPPKILETTYISPSSHLPPNSKPHHIHRHSSSRILSFEDYGSNDMEHEYSPTYLNSIFSPKLEAQVQPHQKSDEFNRKGTKRAQPFSRNQSNAQDHIIAERKRREKLTQRFVALSALVPGLKKMDKASVLGDALKHIKYLQERVGELEEQKKERRLESMVLVKKSKLILDDNNQSFSSSCEDGFSDLDLPEIEVRFSDEDVLIKILCEKQKGHLAKIMAEIEKLHILITNSSVLNFGPTLDITIIAKKESDFDMTLMDVVKSLRSALSNFI

>AtbHLH026

MSNNQAFMELGWRNDVGSLAVKDQGMMSERARSDEDRLINGLKWGYGYFDHDQTDNYLQIVPEIHKEVENAKEDLLVVVPDEHSETDDHHHIKDFSERSDHRFYLRNKHENPKKRRIQVLSSDDESEEFTREVPSVTRKGSKRRRRDEKMSNKMRKLQQLVPNCHKTDKVSVLDKTIEYMKNLQLQLQMMSTVGVNPYFLPATLGFGMHNHMLTAMASAHGLNPANHMMPSPLIPALNWPLPPFTNISFPHSSSQSLFLTTSSPASSPQSLHGLVPYFPSFLDFSSHAMRRL

>AtbHLH027

MEDLDHEYKNYWETTMFFQNQELEFDSWPMEEAFSGSGESSSPDGAATSPASSKNVVSERNRRQKLNQRLFALRSVVPNISKLDKASVIKDSIDYMQELIDQEKTLEAEIRELESRSTLLENPVRDYDCNFAETHLQDFSDNNDMRSKKFKQMDYSTRVQHYPIEVLEMKVTWMGEKTVVVCITCSKKRETMVQLCKVLESLNLNILTTNFSSFTSRLSTTLFLQVTLSLSPSLISLFGNVITSTNYKILNASREYCTCLVLV

>AtbHLH028

MINTDDNLLMIEALLTSDPSPPLLPANLSLETTLPKRLHAVLNGTHEPWSYAIFWKPSYDDFSGEAVLKWGDGVYTGGNEEKTRGRLRRKKTILSSPEEKERRSNVIRELNLMISGEAFPVVEDDVSDDDDVEVTDMEWFFLVSMTWSFGNGSGLAGKAFASYNPVLVTGSDLIYGSGCDRAKQGGDVGLQTILCIPSHNGVLELASTEEIRPNSDLFNRIRFLFGGSKYFSGAPNSNSELFPFQLESSCSSTVTGNPNPSPVYLQNRYNLNFSTSSSTLARAPCGDVLSFGENVKQSFENRNPNTYSDQIQNVVPHATVMLEKKKGKKRGRKPAHGRDKPLNHVEAERMRREKLNHRFYALRAVVPNVSKMDKTSLLEDAVCYINELKSKAENVELEKHAIEIQFNELKEIAGQRNAIPSVCKYEEKASEMMKIEVKIMESDDAMVRVESRKDHHPGARLMNALMDLELEVNHASISVMNDLMIQQANVKMGLRIYKQEELRDLLMSKIS

>AtbHLH031

MDPSGMMNEGGPFNLAEIWQFPLNGVSTAGDSSRRSFVGPNQFGDADLTTAANGDPARMSHALSQAVIEGISGAWKRREDESKSAKIVSTIGASEGENKRQKIDEVCDGKAEAESLGTETEQKKQQMEPTKDYIHVRARRGQATDSHSLAERARREKISERMKILQDLVPGCNKVIGKALVLDEIINYIQSLQRQVEFLSMKLEAVNSRMNPGIEVFPPKEFGQQAFENPEIQFGSQSTREYSRGASPEWLHMQIGSGGFERTSU

>AtbHLH032

MYAMKEEDCLQTFHNLQDYQDQFHLHHHPQILPWSSTSLPSFDPLHFPSNPTRYSDPVHYFNRRASSSSSSFDYNDGFVSPPPSMDHPQNHLRILSEALGPIMRRGSSFGFDGEIMGKLSAQEVMDAKALAASKSHSEAERRRRERINTHLAKLRSILPNTTKTDKASLLAEVIQHMKELKRQTSQITDTYQVPTECDDLTVDSSYNDEEGNLVIRASFCCQDRTDLMHDVINALKSLRLRTLKAEIATVGGRVKNILFLSREYDDEEDHDSYRRNFDGDDVEDYDEERMMNNRVSSIEEALKAVIEKCVHNNDESNDNNNLEKSSSGGIKRQRTSKMVNRCYN

>AtbHLH034

MYPSIEDDDDLLAALCFDQSNGVEDPYGYMQTNEDNIFQDFGSCGVNLMQPQQEQFDSFNGNLEQVCSSFRGGNNGVVYSSSIGSAQLDLAASFSGVLQQETHQVCGFRGQNDDSAVPHLQQQQGQVFSGVVEINSSSSVGAVKEEFEEECSGKRRRTGSCSKPGTKACREKLRREKLNDKFMDLSSVLEPGRTPKTDKSAILDDAIRVVNQLRGEAHELQETNQKLLEEIKSLKADKNELREEKLVLKAEKEKMEQQLKSMVVPSPGFMPSQHPAAFHSHKMAVAYPYGYYPPNMPMWSPLPPADRDTSRDLKNLPPVA

>AtbHLH036

MDDCRDKRRRRCTKLTCGTDNNDMEKMMHRETERQRRQEMASLYASLRSLLPLHFIKGKRSTSDQVNEAVNYIKYLQRKIKELSVRRDDLMVLSRGSLLGSSNGDFKEDVEMISGKNHVVVRQCLVGVEIMLSSRCCGGQPRFSSVLQVLSEYGLCLLNSISSIVDDRLVYTIQAEVNDMALMIDLAELEKRLIRMK

>AtbHLH039

MCALVPPLFPNFGWPSTGEYDSYYLAGDILNNGGFLDFPVPEETYGAVTAVTQHQNSFGVSVSSEGNEIDNNPVVVKKLNHNASERDRRRKINSLFSSLRSCLPASGQSKKLSIPATVSRSLKYIPELQEQVKKLIKKKEELLVQISGQRNTECYVKQPPKAVANYISTVSATRLGDNEVMVQISSSKIHNFSISNVLSGLEEDRFVLVDMSSSRSQGERLFYTLHLQVEKIENYKLNCEELSQRMLYLYEECGNSYI

>AtbHLH043

MNNYNMNPSLFQNYTWNNIINSSNNNNKNDDHHHQHNNDPIGMAMDQYTQLHIFNPFSSSHFPPLSSSLTTTTLLSGDQEDDEDEEEPLEELGAMKEMMYKIAAMQSVDIDPATVKKPKRRNVRISDDPQSVAARHRRERISERIRILQRLVPGGTKMDTASMLDEAIRYVKFLKRQIRLLNNNTGYTPPPPQDQASQAVTTSWVSPPPPPSFGRGGRGVGELI

>AtbHLH044

MANFENLSSDFQTIAMDIYSSITQAADLNNNNSNLHFQTFHPSSTSLESLFLHHHQQQLLHFPGNSPDSSNNFSSTSSFLHSDHNIVDETKKRKALLPTLSSSETSGVSDNTNVIATETGSLRRGKRLKKKKEEEDEKEREVVHVRARRGQATDSHSLAERVRRGKINERLRCLQDMVPGCYKAMGMATMLDEIINYVQSLQNQVEFLSMKLTAASSFYDFNSETDAVDSMQVLEHSNNVFQFLR

>AtbHLH047

MVSKTPSTSSDEANATADERCRKGKVPKRINKAVRERLKREHLNELFIELADTLELNQQNSGKASILCEATRFLKDVFGQIESLRKEHASLLSESSYVTTEKNELKEETSVLETEISKLQNEIEARANQSKPDLNTSPAPEYHHHHYQQQHPERVSQFPGLPIFQGPGFQQSATTLHPPATVLVLPIQPDPQTQDISEMTQAQQPLMFNSSNVSKPCPRYASAADSWSSRLLGERLKASE

>AtbHLH048

MDLTQGFRARSGVVGPVAGLESLNFSDEFRHLVTTMPPETTGGSFTALLEMPVTQAMELLHFPDSSSSQARTVTSGDISPTTLHPFGALTFPSNSLLLDRAARFSVIATEQNGNFSGETANSLPSNPGANLDRVKAEPAETDSMVENQNQSYSSGKRKEREKKVKSSTKKNKSSVESDKLPYVHVRARRGQATDNHSLAERARREKINARMKLLQELVPGCDKIQGTALVLDEIINHVQTLQRQVEMLSMRLAAVNPRIDFNLDSILASENGSLMDGSFNAESYHQLQQWPFDGYHQPEWGREEDHHQANFSMGSATLHPNQVKMEL

>AtbHLH050

MANLSSDFQTFTMDDPIRQLAELSNTLHHFQTFPPPFSSSLDSLFFHNQFPDHFPGKSLENNFHQGIFFPSNIQNNEESSSQFDTKKRKSLMEAVSTSENSVSDQTLSTSSAQVSINGNISTKNNSSRRGKRSKNREEEKEREVVHVRARRGQATDSHSIAERVRRGKINERLKCLQDIVPGCYKTMGMATMLDEIINYVQSLQNQVEFLSMKLTAASSYYDFNSETDAVESMQKAKAREAVEMGQGRDGSSVFHSSSWTL

>AtbHLH052

MIIPETDSFFFQEQPQHQPLYPDEALSPSLFGFDHYDHFYESFLPSQEIFPPGPKTRVFNESQELDSFHTPKHQKLIDSSFHFNSHDPFSPSPESNYLLDSYITEASNISKFQAPDFSSTFKVGWTEQGDTKKRELSAQSIAARKRRRRITEKTQELGKLIPGSQKHNTAEMFNAAAKYVKFLQAQIEILQLKQTKMQTLDSSKVGREMQFLLGSQEIQEKLSTEEVCVVPREMVQVLKAEECILTNPKISRDINKLLSTNLMN

>AtbHLH060

MDLTGGFGARSGGVGPCREPIGLESLHLGDEFRQLVTTLPPENPGGSFTALLELPPTQAVELLHFTDSSSSQQAAVTGIGGEIPPPLHSFGGTLAFPSNSVLMERAARFSVIATEQQNGNISGETPTSSVPSNSSANLDRVKTEPAETDSSQRLISDSAIENQIPCPNQNNRNGKRKDFEKKGKSSTKKNKSSEENEKLPYVHVRARRGQATDSHSLAERARREKINARMKLLQELVPGCDKIQGTALVLDEIINHVQSLQRQVEMLSMRLAAVNPRIDFNLDTILASENGSLMDGSFNAAPMQLAWPQQAIETEQSFHHRQLQQPPTQQWPFDGLNQPVWGREEDQAHGNDNSNLMAVSENVMVASANLHPNQVKMEL

>AtbHLH061

METELTQLRKQESNNLNGVNGGFMAIDQFVPNDWNFDYLCFNNLLQEDDNIDHPSSSSLMNLISQPPPLLHQPPQPSSPLYDSPPLSSAFDYPFLEDIIHSSYSPPPLILPASQENTNNYSPLMEESKSFISIGETNKKRSNKKLEGQPSKNLMAERRRRKRLNDRLSLLRSIVPKITKMDRTSILGDAIDYMKELLDKINKLQEDEQELGSNSHLSTLITNESMVRNSLKFEVDQREVNTHIDICCPTKPGLVVSTVSTLETLGLEIEQCVISCFSDFSLQASCFEVGEQRYMVTSEATKQALIRNAGYGGRCL

>AtbHLH064

MLEGLVSQESLSLNSMDMSVLERLKWVQQQQQQLQQVVSHSSNNSPELLQILQFHGSNNDELLESSFSQFQMLGSGFGPNYNMGFGPPHESISRTSSCHMEPVDTMEVLLKTGEETRAVALKNKRKPEVKTREEQKTEKKIKVEAETESSMKGKSNMGNTEASSDTSKETSKGASENQKLDYIHVRARRGQATDRHSLAERARREKISKKMKYLQDIVPGCNKVTGKAGMLDEIINYVQCLQRQVEFLSMKLAVLNPELELAVEDVSVKQFQAYFTNVVASKQSIMVDVPLFPLDQQGSLDLSAINPNQTTSIEAPSGSWETQSQSLYNTSSLENSCGNYNKISKILLSTKCTHQYVPIRRVWV

>AtbHLH067

MERFQGHINPCFFDRKPDVRSLEVQGFAEAQSFAFKEKEEESLQDTVPFLQMLQSEDPSSFFSIKEPNFLTLLSLQTLKEPWELERYLSLEDSQFHSPVQSETNRFMEGANQAVSSQEIPFSQANMTLPSSTSSPLSAHSRRKRKINHLLPQEMTREKRKRRKTKPSKNNEEIENQRINHIAVERNRRRQMNEHINSLRALLPPSYIQRVNNQKHT

>AtbHLH068

MNRGVLESSPVQQLMAAGNPNWWNVSGGMRPPPPLMGHQQAPLPPHMTPNNNYLRPRMMPTPFPHFLPSPATSSSSSSSSPSLPNNPNLSSWLESNDLPPESWSLSQLLLGGLMMGEEERLEMMNHHNHHDEQQHHGFQGKIRLENWEEQVLSHQQASMVAVDIKQEGNINNNNGYVISSPNSPPNKSCVTTTTTTSLNSNDDNINNNNNMLDFSSNHNGLHLSEGRHTPPDRSSECNSLEIGGSTNKKPRLQPSPSSQSTLKVRKEKLGGRIAALHQLVSPFGKTDTASVLSEAIGYIRFLQSQIEALSHPYFGTTASGNMRHQQHLQGDRSCIFPEDPGQVRPFFLGHYTFNI

>AtbHLH069

MNSSSLLTPSSSPSPHLQSPATFDHDDFLHHIFSSTPWPSSVLDDTPPPTSDCAPVTGFHHHDADSRNQITMIPLSHNHPNDALFNGFSTGSLPFHLPQGSGGQTQTQSQATASATTGGATAQPQTKPKVRARRGQATDPHSIAERLRRERIAERMKSLQELVPNGNKTDKASMLDEIIDYVKFLQLQVKVLSMSRLGGAASASSQISEDAGGSHENTSSSGEAKMTEHQVAKLMEEDMGSAMQYLQGKGLCLMPISLATTISTATCPSRSPFVKDTGVPLSPNLSTTIVANGNGSSLVTVKDAPSVSKP

>AtbHLH070

MFVLRVSNQSFKLHQQVQCKDEIFCLDQKVNVRRSLQVQETVEDHQSFALEEEEQQLSTPSLLQDTTIPFLQMLQQSEDPSPFLSFKDPSFLALLSLQTLEKPWELENYLPHEVPEFHSPIHSETNHYYHNPSLEGVNEAISNQELPFNPLENARSRRKRKNNNLASLMTREKRKRRRTKPTKNIEEIESQRMTHIAVERNRRRQMNVHLNSLRSIIPSSYIQRGDQASIVGGAIDFVKILEQQLQSLEAQKRSQQSDDNKEQIPEDNSLRNISSNKLRASNKEEQSSKLKIEATVIESHVNLKIQCTRKQGQLLRSIILLEKLRFTVLHLNITSPTNTSVSYSFNLKMEDECNLGSADEITAAIRQIFDS

>AtbHLH071

MTLEALSSNGLLNFLLSETLSPTPFKSLVDLEPLPENDVIISKNTISEISNQEPPPQRQPPATNRGKKRRRRKPRVCKNEEEAENQRMTHIAVERNRRRQMNQHLSVLRSLMPQPFAHKGDQASIVGGAIDFIKELEHKLLSLEAQKHHNAKLNQSVTSSTSQDSNGEQENPHQPSSLSLSQFFLHSYDPSQENRNGSTSSVKTPMEDLEVTLIETHANIRILSRRRGFRWSTLATTKPPQLSKLVASLQSLSLSILHLSVTTLDNYAIYSISAKVEESCQLSSVDDIAGAVHHMLSIIEEEPFCCSSMSELPFDFSLNHSNVTHSL

>AtbHLH072

MSNYGVKELTWENGQLTVHGLGDEVEPTTSNNPIWTQSLNGCETLESVVHQAALQQPSKFQLQSPNGPNHNYESKDGSCSRKRGYPQEMDRWFAVQEESHRVGHSVTASASGTNMSWASFESGRSLKTARTGDRDYFRSGSETQDTEGDEQETRGEAGRSNGRRGRAAAIHNESERRRRDRINQRMRTLQKLLPTASKADKVSILDDVIEHLKQLQAQVQFMSLRANLPQQMMIPQLPPPQSVLSIQHQQQQQQQQQQQQQQQQQFQMSLLATMARMGMGGGGNGYGGLVPPPPPPPMMVPPMGNRDCTNGSSATLSDPYSAFFAQTMNMDLYNKMAAAIYRQQSDQTTKVNIGMPSSSSNHEKRD

>AtbHLH073

MGDSDVGDRLPPPSSSDELSSFLRQILSRTPTAQPSSPPKSTNVSSAETFFPSVSGGAVSSVGYGVSETGQDKYAFEHKRSGAKQRNSLKRNIDAQFHNLSEKKRRSKINEKMKALQKLIPNSNKTDKASMLDEAIEYLKQLQLQVQTLAVMNGLGLNPMRLPQVPPPTHTRINETLEQDLNLETLLAAPHSLEPAKTSQGMCFSTATLL

>AtbHLH075

MARFEPYNYNNGHDPFFAHINQNPELINLDLPASTPSSFMLFSNGALVDANHNNSHFFPNLLHGNTRRKGNKEESGSKRRRKRSEEEEAMNGDETQKPKDVVHVRAKRGQATDSHSLAERVRREKINERLKCLQDLVPGCYKAMGMAVMLDVIIDYVRSLQNQIEFLSMKLSAASACYDLNSLDIEPTDIFQGGNIHSAAEMERILRESVGTQPPNFSSTLPF

>AtbHLH076

MSDKDEFAAKKKDLVNTPVDLYPPENPMLGPSPMMDSFRETLWHDGGFNVHTDADTSFRGNNNIDIPLEMGWNMAQFPADSGFIERAAKFSFFGCGEMMMNQQQSSLGVPDSTGLFLQDTQIPSGSKLDNGPLTDASKLVKERSINNVSEDSQSSGGNGHDDAKCGQTSSKGFSSKKRKRIGKDCEEEEDKKQKDEQSPTSNANKTNSEKQPSDSLKDGYIHMRARRGQATNSHSLAERVRREKISERMKFLQDLVPGCDKVTGKAVMLDEIINYVQSLQCQIEFLSMKLSAVNPVLDFNLESLLAKDALQSSAPTFPHNMSMLYPPVSYLSQTGFMQPNISSMSLLSGGLKRQETHGYESDHHNLVHMNHETGTAPDHEDTTADMKVEP

>AtbHLH077

MNMDKETEQTLNYLPLGQSDPFGNGNEGTIGDFLGRYCNNPQEISPLTLQSFSLNSQISENFPISGGIRFPPYPGQFGSDREFGSQPTTQESNKSSLLDPDSVSDRVHTTKSNSRKRKSIPSGNGKESPASSSLTASNSKVSGENGGSKGGKRSKQDVAGSSKNGVEKCDSKGDNKDDAKPPEAPKDYIHVRARRGQATDSHSLAERARREKISERMTLLQDLVPGCNRITGKAVMLDEIINYVQSLQRQVEFLSMKLATVNPRMEFNANASLSTEMIQPGESLTQSLYAMACSEQRLPSAYYSLGKNMPRFSDTQFPSNDGFVHTETPGFWENNDLQSIVQMGFGDILQQQSNNNNNNCSEPTLQMKLEP

>AtbHLH078

MDNELFMNTEFPPPPEMATHFEHQQSSSSAMMLNWALMDPNPHQDSSFLWEKSTEQQQQQSIFDSALSSLVSSPTPSNSNFSGGGGDGFLIRELIGKLGNIGNNNNNSGEIYGTPMSRSASCYATPMSSPPPPTNSNSQMMMNRTTPLTEFSADPGFAERAARFSCFGSRSFNGRTNTNLPINNGNNMVNNSGKLTRVSSTPALKALVSPEVTPGGEFSRKRKSVPKGKSKENPISTASPSPSFSKTAEKNGGKGGSKSSEEKGGKRRREEEDDEEEEGEGEGNKSNNTKPPEPPKDYIHVRARRGQATDSHSLAERVRREKIGERMKLLQDLVPGCNKVTGKALMLDEIINYVQSLQRQVEFLSMKLSSVNDTRLDFNVDALVSKDVMIPSSNNRLHEEGLQSKSSSHHHQQQLNIYNNNSQLLPNISSNNMMLQSPMNSLETSTLARSFTHLPTLTQFTDSISQV

>AtbHLH079

MDPPLVNDSSFSAANPSSYTLSEIWPFPVNDAVRSGLRLAVNSGRVFTRSEHSGNKDVSAAEESTVTDLTAGWGSRKTRDLNSEDDSSKMVSSSSSGNELKESGDKKRKLCGSESGNGDGSMRPEGETSSGGGGSKATEQKNKPEPPKDYIHVRARRGQATDRHSLAERARREKISEKMTALQDIIPGCNKIIGKALVLDEIINYIQSLQRQVEFLSMKLEVVNSGASTGPTIGVFPSGDLGTLPIDVHRTIYEQQEANETRVSQPEWLHMQVDGNFNRTT

>AtbHLH080

MQSTHISGGSSGGGGGGGGEVSRSGLSRIRSAPATWIETLLEEDEEEGLKPNLCLTELLTGNNNSGGVITSRDDSFEFLSSVEQGLYNHHQGGGFHRQNSSPADFLSGSGSGTDGYFSNFGIPANYDYLSTNVDISPTKRSRDMETQFSSQLKEEQMSGGISGMMDMNMDKIFEDSVPCRVRAKRGCATHPRSIAERVRRTRISDRIRRLQELVPNMDKVNSFYKADWF

>AtbHLH081

MQPTSVGSSGGGDDGGGRGGGGGLSRSGLSRIRSAPATWLEALLEEDEEESLKPNLGLTDLLTGNSNDLPTSRGSFEFPIPVEQGLYQQGGFHRQNSTPADFLSGSDGFIQSFGIQANYDYLSGNIDVSPGSKRSREMEALFSSPEFTSQMKGEQSSGQVPTGVSSMSDMNMENLMEDSVAFRVRAKRGCATHPRSIAERVRRTRISDRIRKLQELVPNMDKQTNTADMLEEAVEYVKVLQRQIQELTEEQKRCTCIPKEEQ

> ATbHLH084

MEAMGEWSTGLGGIYTEEADFMNQLLASYEQPCGGSSSETTATLTAYHHQGSQWNGGFCFSQESSSYSGYCAAMPRQEEDNNGMEDATINTNLYLVGEETSECDATEYSGKSLLPLETVAENHDHSMLQPENSLTTTTDEKMFNQCESSKKRTRATTTDKNKRANKARRSQKCVEMSGENENSGEEEYTEKAAGKRKTKPLKPQKTCCSDDESNGGDTFLSKEDGEDSKALNLNGKTRASRGAATDPQSLYARLKQLNKVHCMMVQKRRERINERLRILQHLVPNGTKVDISTMLEEAVQYVKFLQLQIKLLSSDDLWMYAPIAYNGMDIGLDLKLNALTR

>AtbHLH088

MEKLPEFCNPNSSFFSPDHNNTYPFLFNSTHYQSDHSMTNEPGFRYGSGLLTNPSSISPNTAYSSVFLDKRNNSNNNNNGTNMAAMREMIFRIAVMQPIHIDPEAVKPPKRRNVRISKDPQSVAARHRRERISERIRILQRLVPGGTKMDTASMLDEAIHYVKFLKKQVQSLEEQAVVTGGGGGGGGRVLIGGGGMTAASGGGGGGGVVMKGCGTVGTHQMVGNAQILR

>AtbHLH090

MMMMRGGERVKEFLRPFVDSRTWDLCVIWKLGDDPSRFIEWVGCCCSGCYIDKNIKLENSEEGGTGRKKKASFCRDDHNKHRIRTLACEALSRFPLFMPLYPGIHGEVVMSKSPKWLVNSGSKMEMFSTRVLVPVSDGLVELFAFDMRPFDESMVHLIMSRCTTFFEPFPEQRLQFRIIPRAEESMSSGVNLSVEGGGSSSVSNPSSETQNLFGNYPNASCVEILREEQTPCLIMNKEKDVVVQNANDSKANKKLLPTENFKSKNLHSERKRRERINQAMYGLRAVVPKITKLNKIGIFSDAVDYINELLVEKQKLEDELKGINEMECKEIAAEEQSAIADPEAERVSSKSNKRVKKNEVKIEVHETGERDFLIRVVQEHKQDGFKRLIEAVDLCELEIIDVNFTRLDLTVMTVLNVKANKDGIACGILRDLLLKMMITSI

>AtbHLH093

MELSTQMNVFEELLVPTKQETTDNNINNLSFNGGFDHHHHQFFPNGYNIDYLCFNNEEEDENTLLYPSSFMDLISQPPPLLLHQPPPLQPLSPPLSSSATAGATFDYPFLEALQEIIDSSSSSPPLILQNGQEENFNNPMSYPSPLMESDQSKSFSVGYCGGETNKKKSKKLEGQPSKNLMAERRRRKRLNDRLSMLRSIVPKISKMDRTSILGDAIDYMKELLDKINKLQDEEQELGNSNNSHHSKLFGDLKDLNANEPLVRNSPKFEIDRRDEDTRVDICCSPKPGLLLSTVNTLETLGLEIEQCVISCFSDFSLQASCSEGAEQRDFITSEDIKQALFRNAGYGGSCL

>AtbHLH094

MPLEAVVYPQDPFGYLSNCKDFMFHDLYSQEEFVAQDTKNNIDKLGHEQSFVEQGKEDDHQWRDYHQYPLLIPSLGEELGLTAIDVESHPPPQHRRKRRRTRNCKNKEEIENQRMTHIAVERNRRKQMNEYLAVLRSLMPSSYAQRGDQASIVGGAINYVKELEHILQSMEPKRTRTHDPKGDKTSTSSLVGPFTDFFSFPQYSTKSSSDVPESSSSPAEIEVTVAESHANIKIMTKKKPRQLLKLITSLQSLRLTLLHLNVTTLHNSILYSISVRVEEGSQLNTVDDIATALNQTIRRIQEET

>AtbHLH097

MDKDYSAPNFLGESSGGNDDNSSGMIDYMFNRNLQQQQKQSMPQQQQHQLSPSGFGATPFDKMNFSDVMQFADFGSKLALNQTRNQDDQETGIDPVYFLKFPVLNDKIEDHNQTQHLMPSHQTSQEGGECGGNIGNVFLEEKEDQDDDNDNNSVQLRFIGGEEEDRENKNVTKKEVKSKRKRARTSKTSEEVESQRMTHIAVERNRRKQMNEHLRVLRSLMPGSYVQRGDQASIIGGAIEFVRELEQLLQCLESQKRRRILGETGRDMTTTTTSSSSPITTVANQAQPLIITGNVTELEGGGGLREETAENKSCLADVEVKLLGFDAMIKILSRRRPGQLIKTIAALEDLHLSILHTNITTMEQTVLYSFNVKITSETRFTAEDIASSIQQIFSFIHANTNISGSSNLGNIVFT

>AtbHLH099

MMFQQDYPHGFSLVETSLSYEMLDYFQNIVVSNSEDVASQQNSISSSSYSSATLSCSITEQKSHLTEKLSPLRERYGCGDFLSRKRRRRSEKTIVDKENQRMNHIAVERNRRKQMNHFLSILKSMMPLSYSQPNDQASIIEGTISYLKKLEQRLQSLEAQLKATKLNQSPNIFSDFFMFPQYSTATATATATASSSSSSHHHHKRLEVVADVEVTMVERHANIKVLTKTQPRLLFKIINEFNSLGLSTLHLNLTTSKDMSLFTFSVKVEADCQLTPSGNEVANTVHEVVRRVHKER

>AtbHLH100

MCALVPPLYPNFGWPCGDHSFYETDDVSNTFLDFPLPDLTVTHENVSSENNRTLLDNPVVMKKLNHNASERERRKKINTMFSSLRSCLPPTNQTKKLSVSATVSQALKYIPELQEQVKKLMKKKEELSFQISGQRDLVYTDQNSKSEEGVTSYASTVSSTRLSETEVMVQISSLQTEKCSFGNVLSGVEEDGLVLVGASSSRSHGERLFYSMHLQIKNGQVNSEELGDRLLYLYEKCGHSFT

>AtbHLH101

MCTLTPMFPSKQQEWYSASTMEYPWLQSQVHSFSPTLHFPSFLHPLDDSKSHNINLHHMSLSHSNNTNSNNNNYQEEDRGAVVLEKKLNHNASERDRRRKLNALYSSLRALLPLSDQKRKLSIPMTVARVVKYIPEQKQELQRLSRRKEELLKRISRKTHQEQLRNKAMMDSIDSSSSQRIAANWLTDTEIAVQIATSKWTSVSDMLLRLEENGLNVISVSSSVSSTARIFYTLHLQMRGDCKVRLEELINGMLLGLRQS

>AtbHLH103

MTEEFDTTGVCTGTWWSSSNGMFSGCSLPRSAEIVVDFGEIEWQNIDTLDAKTYNENYLSTSTFLGNANLDTTSQIYVSSPSNIHEEERYNQINSFLEGLFDSSEQLLVPNCPKPELFESFHFFDDVFPNESRMISVFDHQKPKEDMQACKSLTTCKRASEKSGELEDIESSQPLKRPRLETPSHFPSFKVRKEKLGDRITALQQLVSPFGKTDTASVLHDAIDYIKFLQEQITEKVSTSPHLNSIGSGEQKQWSDKSSNNTHNQNCSPRQDLRSRGLCLMPISSTFSTPPQHLDTSSLWN

>AtbHLH104

MYPSLDDDFVSDLFCFDQSNGAELDDYTQFGVNLQTDQEDTFPDFVSYGVNLQQEPDEVFSIGASQLDLSSYNGVLSLEPEQVGQQDCEVVQEEEVEINSGSSGGAVKEEQEHLDDDCSRKRARTGSCSRGGGTKACRERLRREKLNERFMDLSSVLEPGRTPKTDKPAILDDAIRILNQLRDEALKLEETNQKLLEEIKSLKAEKNELREEKLVLKADKEKTEQQLKSMTAPSSGFIPHIPAAFNHNKMAVYPSYGYMPMWHYMPQSVRDTSRDQELRPPAA

>AtbHLH107

MQPEVSDQIFYAFLTGGLCASSTSTTVTSSSDPFATVYEDKALASLRNHKEAERKRRARINSHLNKLRKLLSCNSKTDKSTLLAKVVQRVKELKQQTLEITDETIPSETDEISVLNIEDCSRGDDRRIIFKVSFCCEDRPELLKDLMETLKSLQMETLFADMTTVGGRTRNVLVVAADKEHHGVQSVNFLQNALKSLLERSSKSVMVGHGGGGGEERLKRRRALDHIIMV

>AtbHLH108

MNKDEVFLRQWFEILYSLTNPEANSDLRRINNEKGVEKVGQKRSAESRREGKKKRVKTQCVIKSSDKSDHDTLLKKKRRERIRRQLETLKEITPNCPQSDINAILDCVIEYTNNLRLAHYKGSQGICDDWRLFTEAGAVLYYIDT

>AtbHLH109

MERNNRNEGTHEEEQCSLSDIIYSFCSENHSELNPLQEIFGVTKNNDHEKHDEEPDEESYRMAKRQRSMEYRMMMEKKRRKEIKDKVDILQGLMPNHCTKPDLASKLENIIEYIKSLKYQVDVMSMAYTTTPVYTPPFYAAAQAPCMSPWGYYTPGVPMMPQQNMTYIPQYPQVYGTVPPNQTQP

>AtbHLH110

MDSANLHQLQDQLQLVGSSSSSSSLDNNSDPSCYGASSAHQWSPGGISLNSVSLSHNYNNEMLNTRAHNNNNNNNTSECMSLSSIHNHSLIQQQDFPLQWPHDQSSYQHHEGLLKIKEELSSSTISDHQEGISKFTDMLNSPVITNYLKINEHKDYTEKLLLKSMSSGFPINGDYGSSLPSSSSSSSPSSQSHRGNFSQIYPSVNISSLSESRKMSMDDMSNISRPFDINMQVFDGRLFEGNVLVPPFNAQEISSLGMSRGSLPSFGLPFHHHLQQTLPHLSSSPTHQMEMFSNEPQTSEGKRHNFLMATKAGENASKKPRVESRSSCPPFKVRKEKLGDRIAALQQLVSPFGKTDTASVLMEAIGYIKFLQSQIETLSVPYMRASRNRPGKASQLVSQSQEGDEEETRDLRSRGLCLVPLSCMTYVTGDGGDGGGGVGTGFWPTPPGFGGGT

>AtbHLH111

MLREECTPSSSWWEDVQHHHNDHANSISSTSFYHKSSNNNSHANASCEEDNLSVSTVRASNRLDLTAESSNHHSLSASNQPASSSDELLRDHVVSSHNHLWSLAFLPGRSLGDQMMDHHHHIASRNSSTTSELPSFEPACHNGNGNGWIYDPNQVRYDQSSDQRLSKLTDLVGKHWSIAPPNNPDMNHNLHHHFDHDHSQNDDISMYRQALEVKNEEDLCYNNGSSGGGSLFHDPIESSRSFLDIRLSRPLTDINPSFKPCFKALNVSEFNKKEHQTASLAAVRLGTTNAGKKKRCEEISDEVSKKAKCSEGSTLSPEKELPKAKLRDKITTLQQIVSPFGKTDTASVLQEAITYINFYQEQVKLLSTPYMKNSSMKDPWGGWDREDHNKRGPKHLDLRSRGLCLVPISYTPIAYRDNSATDYWNPTYRGSLYR

>AtbHLH112

MAEEFKATASICGGGGGAWWNSPRSVMSPSDHFLSPCFGAAITSNDFSSQENHLKSRMTCTDNNNIVFGQREADSDSGGSTVTMDSTLQMMGLGFSSNCSSDWNQTILQEDLNSSFIRSSQDQDHGQGFLSTTTSPYILNPACSSSPSTSSSSSLIRTFYDPEPSPYNFVSTTSGSINDPQLSWANKTNPHHQVAYGLINSFSNNANSRPFWNSSSTTNLNNTTPSNFVTTPQIISTRLEDKTKNLKTRAQSESLKRAKDNESAAKKPRVTTPSPLPTFKVRKENLRDQITSLQQLVSPFGKTDTASVLQEAIEYIKFLHDQVTVLSTPYMKQGASNQQQQQISGKSKSQDENENHELRGHGLCLVPISSTFPVANETTADFWTPTFGGNNFR

>AtbHLH113

MGDTAEDQDDRAMMEAEGVTSFSELLMFSDGVLSSSSDHQPEGNVGDGGEDSLGFVFSGKTGSRMLCFSGGYQNDDESLFLEPSVPTSGVSDLDPSCIKIDCRNSNDACTVDKSTKSSTKKRTGTGNGQESDQNRKPGKKGKRNQEKSSVGIAKVRKERLGERIAALQQLVSPYGKTDAASVLHEAMGYIKFLQDQIQVLCSPYLINHSLDGGVVTGDVMAAMKAKDLRSRGLCLVPVSSTVHVENSNGADFWSPATMGHTTSPSLPQGF

>AtbHLH114

MSHATSTLNSKVNYTIKNMTEEFEIAGISTGAWWSSPTNTAAVFSGYSLPCSTEISPDVTNFGWQNFDNKINDHNDGCMNMHNSFFEGLLIDPNDQLLPDPWSKSTIPNAKSELLENFPFLDNMFLVDSEAESLLDHEIRNHKSSKEQITQDYKNLTSKRSEELEENSDEYSPRLLKRPRLETLSPLPSFKVRKEKLGDRITALQQLVSPFGKTDTASVLNEAVEYIKFLQEQVTVLSNPEQNTIGSVQQQQCSNKKSINTQGEVEEDECSPRRYVDLSSRGLCLMPISASYPVAAAAASAAEMNVHLVSGIFHSL

>AtbHLH115

MVSPENTNWLSDYPLIEGAFSDQNPTFPWQIDGSATVSVEVDGFLCDADVIKEPSSRKRIKTESCTGSNSKACREKQRRDRLNDKFTELSSVLEPGRTPKTDKVAIINDAIRMVNQARDEAQKLKDLNSSLQEKIKELKDEKNELRDEKQKLKVEKERIDQQLKAIKTQPQPQPCFLPNPQTLSQAQAPGSKLVPFTTYPGFAMWQFMPPAAVDTSQDHVLRPPVA

>AtbHLH117

METPAYDFDSLTDLPPLPPSDFTPSNAFTFPDHNLDFSFLDSTLSLLNRHHLSESTRLEQIFYDSTHTQLFHNDDTTTTTTPFLHLPDLKSIDAVEEPTTMKLFPSLSPPLPAAKRQKLNSTSSSTTSGSPTASNDGGIITKRRKISDKIRSLEKLMPWERKMNLAMTLEESHKYIKFLQSQIASLRWMPLESVYNTAGEVGETDLLKSLTRQQILQVLANSPGSRNVLSSRGVCVFSYEQLLSLKTMSRNL

>AtbHLH118

MNNFQEKKRRRSKTPRVCNNEENMEKLVHKEIEKRRRQEMASLYASLRSLLPLEFIQGKRSTSDQVKGAVNYIDYLQRNIKDINSKRDDLVLLSGRSFRSSNEQEWNEISNHVVIRPCLVGIEIVLSILQTPFSSVLQVLREHGLYVLGYICSSVNDRLIHTLQAEVNDLALIDLADLKDTLTLMK

>AtbHLH119

MGEDDIVELLWNGQVVRTSQPQRPSSGKPSPTPPILRGSGSGSGEENAPLPLPLLQPPRPLHHQNLFIREEEMSSWLHYSYTGVTSTPATHPQSSVSLPPPPPIAPSEDDVVELLWKSGQVVQSIQTQRPIPPPIFRGSGSGGGEETVLPLPPLHPSHQNIFIQEDEMASWLYHPLRQDYFSSGVASTSATRPQSSASLAPTPPPPSVPYGQIPVERRTENFMNFLRLRGNIFSGGRVEAGPVVIESTQIGSSATPSSSAAESCVIPATHGTESRAAAITGVSRTFAVPGLGRRGKEVATETAGTSYSGVNKAETERVQIQPERETKITEDKKREETIAEIQGTEEAHGSTSRKRSRAADMHNLSERRRRERINERMKTLQELLPRCRKTDKVSMLEDVIEYVKSLQLQIQMMSMGHGMMPPMMHEGNTQQFMPHMAMGMKGMNRPPPFVPFPGKTFPRPGHMAGVGPSYPALRYPFPDTQASDLSRVHVPSLHSNPVPNQPRFPAYINPYSQFVGLHQMQQPPLPLQGQPTSQPSFSHASTSK

>AtbHLH120

MKTTPLPRLHYLVSLLCFFLSSKIKEDRPNYVRAVSPINLTSSLEKTREKKKRLLLRSTISKPQPMNPSNNPKKTRHQSHMPQERDETKKEKKLLHRNIERQRRQEMAILFASLRSQLPLKYIKGKRAMSDHVNGAVSFIKDTQTRIKDLSARRDELKREIGDPTSLTGSGSGSGSSRSEPASVMVQPCVSGFEVVVSSLASGLEAWPLSRVLEVLHGQGLEVISSLTARVNERLMYTIQVEVRLHIWSYCRVF

>AtbHLH121

MDVSARKSQKAGREKLRREKLNEHFVELGNVLDPERPKNDKATILTDTVQLLKELTSEVNKLKSEYTALTDESRELTQEKNDLREEKTSLKSDIENLNLQYQQRLRSMSPWGAAMDHTVMMAPPPSFPYPMPIAMPPGSIPMHPSMPSYTYFGNQNPSMIPAPCPTYMPYMPPNTVVEQQSVHIPQNPGNRSREPRAKVSRESRSEKAEDSNEVATQLELKTPGSTSDKDTLQRPEKTKRCKRNNNNNSIEESSHSSKCSSSPSVRDHSSSSSVAGGQKPDDAK

>AtbHLH122

MESEFQQHHFLLHDHQHQRPRNSGLIRYQSAPSSYFSSFGESIEEFLDRPTSPETERILSGFLQTTDTSDNVDSFLHHTFNSDGTEKKPPEVKTEDEDAEIPVTATATAMEVVVSGDGEISVNPEVSIGYVASVSRNKRPREKDDRTPVNNLARHNSSPAGLFSSIDVETAYAAVMKSMGGFGGSNVMSTSNTEASSLTPRSKLLPPTSRAMSPISEVDVKPGFSSRLPPRTLSGGFNRSFGNEGSASSKLTALARTQSGGLDQYKTKDEDSASRRPPLAHHMSLPKSLSDIEQLLSDSIPCKIRAKRGCATHPRSIAERVRRTKISERMRKLQDLVPNMDTQTNTADMLDLAVQYIKDLQEQVKALEESRARCRCSSA

>AtbHLH123

MGDHHDFINSGSWWKVSSSSSPSSSSSMRASSIESGGSAVFHDKLHHHSLATDHHLQMIGLGLSSQSPVDQWNQSLLRGDSKAETSFGVMLQENLNLDATSNANANTTSSTSSYQLQESDSSHHHQALWRDPQSDFKPQILTSGGNRGFFLDHQFSPHGSSSTDSSTVTCQGFAVDNSSNAMYAATTTTPNSSSGMFHHQQAGGFGSSDQQPSRNHQQSSLGYSQFGSSTGNYDQMASALPSTWFLRSSPPPKPHSPLRFSNNATFWNPAAAGNAGAPPPHDASSNFFPALQPPQIHPQSFDEQPKNISEIRDSSSNEVKRGGNDHQPAAKRAKSEAASPSPAFKRKEKMGDRIAALQQLVSPFGKTDAASVLSEAIEYIKFLHQQVSALSNPYMKSGASLQHQQSDHSTELEVSEEPDLRSRGLCLVPVSSTFPVTHDTTVDFWTPTFGGTFR

>AtbHLH124

MEAKPLASSSSEPNMISPSSNIKPKLKDEDYMELVCENGQILAKIRRPKNNGSFQKQRRQSLLDLYETEYSEGFKKNIKILGDTQVVPVSQSKPQQDKETNEQMNNNKKKLKSSKIEFERNVSKSNKCVESSTLIDVSAKGPKNVEVTTAPPDEQSAAVGRSTELYFASSSKFSRGTSRDLSCCSLKRKYGDIEEEESTYLSNNSDDESDDAKTQVHARTRKPVTKRKRSTEVHKLYERKRRDEFNKKMRALQDLLPNCYKDDKASLLDEAIKYMRTLQLQVQMMSMGNGLIRPPTMLPMGHYSPMGLGMHMGAAATPTSIPQFLPMNVQATGFPGMNNAPPQMLSFLNHPSGLIPNTPIFSPLENCSQPFVVPSCVSQTQATSFTQFPKSASASNLEDAMQYRGSNGFSYYRSPN

>AtbHLH125

MDCVPSLFMPDSTYEDGLLFSDSFLLSPFISYQNNDVFHSITNKIGGSNKKRSLCDITYGANEANKNDDDRESKKMKHRDIERQRRQEVSSLFKRLRTLLPFQYIQGKRSTSDHIVQAVNYIKDLQIKIKELNEKRNRVKKVISATTTTHSAIEECTSSLSSSAASTLSSSCSCVGDKHITVVVTPCLVGVEIIISCCLGRNKSCLSSVLQMLAQEQRFSVVSCLSARRQQRFMHTIVSQVEDGKQINILELKDKIMTM

>AtbHLH126

MDPYKNLNPKGYQRQRPFSSAGESGGSGGSGTAHETDDNKKKKKLLHRDIERQRRQEMATLFATLRTHLPLKYIKGKRAVSDHVNGAVNFIKDTEARIKELSARRDELSRETGQGYKSNPDPGKTGSDVGKSEPATVMVQPHVSGLEVVVSSNSSGPEALPLSKVLETIQEKGLEVMSSFTTRVNDRLMHTIQVEVNSFGCIDLLWLQQKLVEDLILSTGY

>AtbHLH127

MSSWPHHPLRQDYLCSELYASTPAPHPQSSVSLAPPPPKPPSSAPYGQIIAPRSAPRIQGTEEARGSTSRKRSRAAEMHNLAERRRREKINERMKTLQQLIPRCNKSTKVSMLEDVIEYVKSLEMQINQFMPHMAMGMNQPPAYIPFPSQAHMAGVGPSYPPPRYPFPNIQTFDPSRVWLQSPQPNPVSNQPQMNPYGQFVGHHQMQQSLPPPLQVILSQYPLCLFLCSNK

>AtbHLH129

MYPPNSSKSTAHDGGGDADTNQYDSAAGATRDFSSLGPQTHHHPPPQRQQQHQQNPNLVGHYLPGEPSSIGFDSNASSSSSLFRHRSSPAGFYDQHLPTDPNGFSLGRPNGGYGGGGEQGPSRLKSELRFSSGSSSHQEHNSLPRISEVEAAAAARNGVASSSMSFGNNRTNNWDNSSSHISFTIDQPGKRSKNSDFFTLETQYSMPQTTLEMATMENLMNIPEDSVPCRARAKRGFATHPRSIAERERRTRISGKLKKLQELVPNMDKQTSYADMLDLAVEHIKGLQHQVESLEKGMERCTCGACKKR

>AtbHLH132

MELVFENGQILAKGQRSNVSLHNQRTKSIMDLYEAEYNEDFMKSIIHGGGGAITNLGDTQVVPQSHVAAAHETNMLESNKHVDDSETLKASSSKRMMVDYHNRKKIKFIPPDEQSVVADRSFKLGFDTSSVGFTEDSEGSMYLSSSLDDESDDARPQVPARTRKALVKRKRNAEAYNSPERDDNESMLDEAINYMTNLQLQVQMMTMGNRFVTPSMMMPLGPNYSQMGLAMGVGMQMGEQQFLPAHVLGAGLPGINDSADMLRFLNHPGLMPMQNSAPFIPTENCSPQSVPPSCAAFPNQIPNPNSLSNLDGATLHKKSRKTNR

>AtbHLH133

MNRGVLESSPVQHLTAAGNPNWWNNVSRGLRPPTPLMSHEPPSTTAFIPSLLPNFFSSPTSSSSSSPSFPPPNSNPNFSSWLEMSDLPLDQPWSLSQLLLGGLMMGEEEKMEMMNHHHHQNQHQSYQAKRIQNWEEQVLRHQASMKQESSNNNSYGIMSSPNSPPNKSCATIINTNEDNNNNIHSGLNLSECNSSEMIGSSFANKKPKLQVPSSQSTLKVRKEKLGGRIASLHQLVSPFGKTDTASVLSEAIGYIRFLHSQIEALSLPYFGTPSRNNMMHQHAQRNMNGIFPEDPGQVRTYIFLLSSFIYFTFYWIN

>AtbHLH134

MSSSRRSRQASSSSRISDDQITDLISKLRQSIPEIRQNRRSNTVSASKVLQETCNYIRNLNKEADDLSDRLTQLLESIDPNSPQAAVIRSLING

>AtbHLH135

MSGRRSRSRQSSGTSRISEDQINDLIIKLQQLLPELRDSRRSDKVSAARVLQDTCNYIRNLHREVDDLSERLSELLANSDTAQAALIRSLLTQ

>AtbHLH136

MSNRRSRQSSSAPRISDNQMIDLVSKLRQILPEIGQRRRSDKASASKVLQETCNYIRNLNREVDNLSERLSQLLESVDEDSPEAAVIRSLLM

>AtbHLH138

MERYTKKNERFKAEEGKGSKKSRTFLTERERRALFNDRFFDLKNLIPNPTKGGEASIVQDGIVYINELQRLVSELKYLVEKKKCGARHNNIEVDNKNTIYGTSKIEHPFSKNKNTFNCLIRTLRFVHHF

>AtbHLH139

MENEAFVDGELESLLGMFNFDQCSSNESSFCNAPNETDVFSSDDFFPFGTILQSNYAAVLDGSNHQTNRNVDSRQDLLKPRKKQKLSSESNLVTEPKTAWRDGQSLSSYNSSDDEKALGLVSNTSKSLKRKAKANRGIASDPQSLYARKRRERINDRLKTLQSLVPNGTKVDISTMLEDAVHYVKFLQLQIKLLSSEDLWMYAPLAHNGLNMGLHHNLLSRLI

>AtbHLH140

MEVQIEESGKNQTSPESIEADKAKQIVVLLIGPPGSGKSTFCDTAMRSSHRPWSRICQDIVNNGKAGTKAQCLKMATDSLREGKSVFIDRCNLDREQRSEFIKLGGPEFEVHAVVLELPAQVCISRSVKRTGHEGNLQGGRAAAVVNKMLQSKELPKVNEGFSRIMFCYSDADVDNAVNMYNKLGPMDTLPSGCFGEKKLDTKSQPGIMKFFKKVSALPASSSNEATNTTRKADEMTANVRVSPVKLGSADIVPTLAFPSISTADFQFDLEKASDIIVEKAEEFLSKLGTARLVLVDLSRGSKILSLVKAKASQKNIDSAKFFTFVGDITKLRSEGGLHCNVIANATNWRLKPGGGGVNAAIFKAAGPDLETATRVRANTLLPGKAVVVPLPSTCPLHNAEGITHVIHVLGPNMNPNRPDNLNNDYTKGCKTLREAYTSLFEGFLSVVQDQSKLPKRSSQTAVSDSGEDIKEDSERNKKYKGSQDKAVTNNLESESLEDTRGSGKKMSKGWNTWALALHSIAMHPERHENVVLEYLDNIVVINDQYPKARKHVLVLARQESLDGLEDVRKENLQLLQEMHNVGLKWVDRFQNEDASLIFRLGYHSVPSMRQLHLHVISQDFNSDSLKNKKHWNSFTTSFFRDSVDVLEEVNSQGKANVASEDLLKGELRCNRCRSAHPNIPKLKSHVRSCHSQFPDHLLQNNRLVARAET

>AtbHLH141

MNSHDIDDQLEADVYSNLPSRNDSSTGRRNRNSCRSKHSETEQRRRSKINERFQSLMDIIPQNQNDQKRDKASFLLEVIEYIHFLQEKVHMYEDSHQMWYQSPTKLIPWRNSHGSVAEENDHPQIVKSFSSNDKVAASSGFLLDTYNSVNPDIDSAVSTKIPEHSPVSAVSSYLRTEPSLQFVQHDFWQPKTSCGTINCFTNELLTSDEKTSASLSTVCSQRVLNTLTEALKSSGVNMSETMISVQLSLRKREDREYSVAAFASEDNGNSIADEEGDSPTETRSFCNDIDHSQKRIRR

>AtbHLH142

MPLDKRQRDLPLGLSPQACFKDIVGRSVLPRIPLPELGKLYAAKLQARCLQPPPFQSLLCSHDKESYGKRFSRSDMRSWCAAATTTTTPLGALESSQKRLLIFDQSGDQTRLLQCPFPLRFPSHAAAEPVKLSELQGIEKAFKEDGEEFHKSDGTESEMHEDTEEINALLYSDDDYDDDCESDDEVMSTGHSPYPNEGVCNKRELEEIDGPCKRQKLLDKVNNISDLSSLVGTESSTQLNGSSFLKDKKLPESKTISTKEDTGSGLSNEQSKKDKIRTALKILESVVPGAKGNEALLLLDEAIDYLKLLKRDLISTEVKNQSSTTHKSPILLLKETTWGTRNLQTDKA

>AtbHLH143

MPLDTKQQKWLPLGLNPQACVQDKATEYFRPGIPFPELGKVYAAEHQFRYLQPPFQALLSRYDQQSCGKQVSCLNGRSSNGAAPEGALKSSRKRFIVFDQSGEQTRLLQCGFPLRFPSSMDAERGNILGALHPEKGFSKDHAIQEKILQHEDHENGEEDSEMHEDTEEINALLYSDDDDNDDWESDDEVMSTGHSPFTVEQQACNITTEELDETESTVDGPLLKRQKLLDHSYRDSSPSLVGTTKVKGLSDENLPESNISSKQETGSGLSDEQSRKDKIHTALRILESVVPGAKGKEALLLLDEAIDYLKLLKQSLNSSKGLNNHW

>AtbHLH144

MQNNQFPHFSDEVGDRNMHNPYASGSSYDALFPPCAKLPYHGVELQPSAVCPKNFVIFDQTYDRSQVMYHPELTHKLMNTPSLNNLASTFQNEYVGGSYGNYGNYEQEVSSSYQEDPNEIDALLSADEDYEENDDNEGEEDGGDSEEVSTARTSSRDYGNTTAESCCSSYGYNNNNNNNSRKQSLSGSASSSNNDGKGRKKMKKMMGVLRRIVPGGEQMNTACVLDEAVQYLKSLKIEAQKLGVGHFSNQS

>AtbHLH145

MGQDRGFGFPTQRLCSLSSLALSHLGKQDLNLVSKTCGDTTDMFSTRGSYQVSTQVSQSYFDGYCGWVHGSSHLQQQFLPPQNQCMKQVPLQVDGVISKAEEQCSQKRFLVFDQSGDQTTLLLASDIRKSFETLKQHACPDMKEELQRSNKDLFVCHGMQGNSEPDLKEDSEELNALLYSEDESGYCSEEDEVTSADHSPSIVVSGREDQKTFLGSYGQPLNAKKRKILETSNESMRDAESSCGSCDNTRISFLKRSKLSSNKIGEEKIFETVSLLRSVVPGEELVDPILVIDRAIDYLKSLKMEAKNREA

>AtbHLH146

MERQIINRKKRVFSLEPNKNPSAVFTRKYTSHLVPALKKLNMNKNSSKQTVKHEVDMALALSAQEFAWSRFLLQKLSSSSNPTTTTSSSSDGIRILERPDKEGGNEEGGIEERLRELKKLLPGGEEMNVEEMLSEIGNYIKCLELQTIALKSIVQDST

>AtbHLH147

MESISPVSNQLLQPTTTSSNSDRSRRKRKKKSSPSSVEKSPSPSISLEKWRSEKQQQIYSTKLVHALRELRISQQPSSSSSSSIPRGGRAVREVADRALAVAARGKTLWSRAILSKAVKLKFRKHKRQRISNPTTTTLTTGSIRSKKQRATVLRLKAKGLPAVQRKVKVLSRLVPGCRKQSLPVVLEETTDYIAAMEMQIRTMTAILSAVSSSPPPPTPGHEGGQTHMLG

>AtbHLH148

MASLISDIEPPTSTTSDLVRRKKRSSASSAASSRSSASSVSGEIHARWRSEKQQRIYSAKLFQALQQVRLNSSASTSSSPTAQKRGKAVREAADRALAVSARGRTLWSRAILANRIKLKFRKQRRPRATMAIPAMTTVVSSSSNRSRKRRVSVLRLNKKSIPDVNRKVRVLGRLVPGCGKQSVPVILEEATDYIQALEMQVRAMNSLVQLLSSYGSAPPPI

>AtbHLH149

MVESLFPSIENTGESSRRKKPRISETAEAEIEARRVNEESLKRWKTNRVQQIYACKLVEALRRVRQRSSTTSNNETDKLVSGAAREIRDTADRVLAASARGTTRWSRAILASRVRAKLKKHRKAKKSTGNCKSRKGLTETNRIKLPAVERKLKILGRLVPGCRKVSVPNLLDEATDYIAALEMQVRAMEALAELLTAAAPRTTLTGT

>AtbHLH150

MSSEQGNGSNPSTSPEVEGTKTIPFRRRLQRGQRVFAPKLMEALRRSRVSSEEAPVRHLSRRWRATTAQKVYSLKLYDALQRSRRSATVRDTADKVLATTARGATRWSRAILVSRFGTSLRRRRNTKPASALAAAIRGSGGSGRRRKLSAVGNRVRVLGGLVPGCRRTALPELLDETADYIAALEMQVRAMTALSKILSELQPSTNLGSAL

>AtbHLH151

MGVTLEGQRKESIWVLMRRQRARRALVKKIMIRPRKSVEASRRPCRAIHRRVKTLKELVPNTKTSEGLDGLFRQTADYILALEMKVKVMQTMVQVLTETN

CV

>AtbHLH152

MGSRFVSNEQKPHVVCVPYPAQGHINPMMKVAKLLHVKGFHVTFVNTVYN

HNRLLRSRGANALDGLPSFQFESIPDGLPETGVDATQDIPALSESTTKNCLVPFKKLLQRIVTREDVPPVSCIVSDGSMSFTLDVAEELGVPEIHFWTTSACGFMAYLHFYLFIEKGLCPVKDASCLTKEYLDTVIDWIPSMNNVKLKDIPSFIRTTNPNDIMLNFVVREACRTKRASAIILNTFDDLEHDIIQSMQSILPPVYPIGPLHLLVNREIEEDSEIGRMGSNLWKEETECLGWLNTKSRNSVVYVNFGSITIMTTAQLLEFAWGLAATGKEFLWVMRPDSVAGEEAVIPKEFLAETADRRMLTSWCPQEKVLSHPAVGGFLTHCGWNSTLESLSCGVPMVCWPFFAEQQTNCKFSCDEWEVGIEIGGDVKRGEVEAVVRELMDGEKGKKMREKAVEWRRLAEKATKLPCGSSVINFETIVNKVLLGKIPNT

>AtbHLH153

MEFSRDAGMMMENKRNVCSLGESSIKRHKSDLSFSSKERKDKVGERISALQQIVSPYGKTDTASVLLDAMHYIEFLHEQVKVCSSIPSMIHSSLSEFPCSFVQVLSAPYLQTVPDATQEELEQYSLRNRGLCLVPMENTVGVAQSNGADIWAPVKTPLSPAFSVTSQSPFR

>AtbHLH154

MEYSRDSAEMMMETKRNVYSLEDNKIKRHKSSDLSFSSKERKDKLAERISALQQLVSPYGKTDTASVLLEGMQYIQFLQEQVKVLSAPYLQATPSTTEEEVEEYSLRSKGLCLVPLEYTSEVAQTNGADIWAPVKTPTSSHAFNLSSSNSPFQ

>AtbHLH155

MGSTSQEILKSFCFNTDWDYAVFWQLNHRGSRMVLTLEDAYYDHHGTNMHGAHDPLGLAVAKMSYHVYSLGEGIVGQVAVSGEHQWVFPENYNNCNSAFEFHNVWESQISAGIKTILVVAVGPCGVVQLGSLCKVNEDVNFVNHIRHLFLALRDPLADHAANLRQCNMNNSLCLPKMPSEGLHAEAFPDCSGEVDKAMDVEESNILTQYKTRRSDSMPYNTPSSCLVMEKAAQVVGGREVVQGSTCGSYSGVTFGFPVDLVGAKHENQVGTNIIRDAPHVGMTSGCKDSRDLDPNLHLYMKNHVLNDTSTSALAIEAERLITSQSYPRLDSTFQATSRTDKESSYHNEVFQLSENQGNKYIKETERMLGRNCESSQFDALISSGYTFAGSELLEALGSAFKQTNTGQEELLKSEHGSTMRPTDDMSHSQLTFDPGPENLLDAVVANVCQRDGNARDDMMSSRSVQSLLTNMELAEPSGQKKHNIVNPINSAMNQPPMAEVDTQQNSSDICGAFSSIGFSSTYPSSSSDQFQTSLDIPKKNKKRAKPGESSRPRPRDRQLIQDRIKELRELVPNGSKCSIDSLLERTIKHMLFLQNVTKHAEKLSKSANEKMQQKETGMQGSSCAVEVGGHLQVSSIIVENLNKQGMVLIEMLCEECGHFLEIANVIRSLDLVILRGFTETQGEKTWICFVTESQNSKVMQRMDILWSLVQIFQPKANEKG

>AtbHLH156

MGVLLREALRSMCVNNQWSYAVFWKIGCQNSSLLIWEECYNETESSSNPRRLCGLGVDTQGNEKVQLLTNRMMLNNRIILVGEGLVGRAAFTGHHQWILANSFNRDVHPPEVINEMLLQFSAGIQTVAVFPVVPHGVVQLGSSLPIMENLGFVNDVKGLILQLGCVPGALLSENYRTYEPAADFIGVPVSRIIPSQGHKILQSSAFVAETSKQHFNSTGSSDHQMVEESPCNLVDEHEGGWQSTTGFLTAGEVAVPSNPDAWLNQNFSCMSNVDAAEQQQIPCEDISSKRSLGSDDLFDMLGLDDKNKGCDNSWGVSQMRTEVLTRELSDFRIIQEMDPEFGSSGYELSGTDHLLDAVVSGACSSTKQISDETSESCKTTLTKVSNSSVTTPSHSSPQGSQLFEKKHGQPLGPSSVYGSQISSWVEQAHSLKREGSPRMVNKNETAKPANNRKRLKPGENPRPRPKDRQMIQDRVKELREIIPNGAKCSIDALLERTIKHMLFLQNVSKHSDKLKQTGESKIMKEDGGGATWAFEVGSKSMVCPIVVEDINPPRIFQVEMLCEQRGFFLEIADWIRSLGLTILKGVIETRVDKIWARFTVEASRDVTRMEIFMQLVNILEQTMKCGGNSKTILDGIKATMPLPVTGGCSM

>AtbHLH157

MGSEYKHILKSLCLSHGWSYAVFWRYDPINSMILRFEEAYNDEQSVALVDDMVLQAPILGQGIVGEVASSGNHQWLFSDTLFQWEHEFQNQFLCGFKTIAIIPLGSSGVVQLGSTQKILESTEILEQTTRALQETCLKPHDSGDLDTLFESLGDCEIFPAESFQGFSFDDIFAEDNPPSLLSPEMISSEAASSNQDLTNGDDYGFDILQSYSLDDLYQLLADPPEQNCSSMVIQGVDKDLFDILGMNSQTPTMALPPKGLFSELISSSLSNNTCSSSLTNVQEYSGVNQSKRRKLDTSSAHSSSLFPQEETVTSRSLWIDDDERSSIGGNWKKPHEEGVKKKRAKAGESRRPRPKDRQMIQDRIKELRGMIPNGAKCSIDTLLDLTIKHMVFMQSLAKYAERLKQPYESKVMNQIYPFSKYTSVUVLM

>AtbHLH158

MASADKLINTDVPEKDVFAFHFLQSLSNLRKQNPFDTPDQKNYRVRKIKKAAYVSMARAAGGSSRLWSRALLRRADKDDNKIVRFSRRKWKISSKRRRSNQRAPVVEEAAERLRNLVPGGGGMETSKLMEETAHYIKCLSMQVKVMQCLVDGLSPK

>AtbHLH159

MQPTSSMNEEFLKKWQMGLQIFRPSIDNTSVHERKKAIKLSADVAMASLRKGTTCWSRALIEKTATEDNFLVRQMLSGIKAETLINKKLPKKTVCHRKIVRRSKKILRRKSKSASEEAAAKAKRLVKRRTQGLRNVVPGGELMSNDVLLLQETLDYIVSLQTQVNVMRSIVDAAEAEIER

>AtbHLH160

MRLHASYLTLGTLLPDHSSSSSKKKWSAPSIIDNVITYIPKLQNEVGELTLRKQKLVELERRGPSIRAISVLELGESGYEAVVQICLKKENEDEFSNLLHVMEVQGLSVLSASTSQVCREQRVVCYNFHVKMDEKPCEGDDYITVLKNNIISSLRDNTKCK

>AtbHLH016

MSQCVPNCHIDDTPAAATTTVRSTTAADIPILDYEVAELTWENGQLGLHGLGPPRVTASSTKYSTGAGGTLESIVDQATRLPNPKPTDELVPWFHHRSSRAAMAMDALVPCSNLVHEQQSKPGGVGSTRVGSCSDGRTMGGGKRARVAPEWSGGGSQRLTMDTYDVGFTSTSMGSHDNTIDDHDSVCHSRPQMEDEEEKKAGGKSSVSTKRSRAAAIHNQSERKRRDKINQRMKTLQKLVPNSSKTDKASMLDEVIEYLKQLQAQVSMMSRMNMPSMMLPMAMQQQQQLQMSLMSNPMGLGMGMGMPGLGLLDLNSMNRAAASAPNIHANMMPNPFLPMNCPSWDASSNDSRFQSPLIPDPMSAFLACSTQPTTMEAYSRMATLYQQMQQQLPPPSNPK

>AtbHLH105

MVSPENANWICDLIDADYGSFTIQGPGFSWPVQQPIGVSSNSSAGVDGSAGNSEASKEPGSKKRGRCESSSATSSKACREKQRRDRLNDKFMELGAILEPGNPPKTDKAAILVDAVRMVTQLRGEAQKLKDSNSSLQDKIKELKTEKNELRDEKQRLKTEKEKLEQQLKAMNAPQPSFFPAPPMMPTAFASAQGQAPGNKMVPIISYPGVAMWQFMPPASVDTSQDHVLRPPVA

>AtbHLH001

MATGQNRTTVPENLKKHLAVSVRNIQWSYGIFWSVSASQSGVLEWGDGYYNGDIKTRKTIQASEIKADQLGLRRSEQLSELYESLSVAESSSSGVAAGSQVTRRASAAALSPEDLADTEWYYLVCMSFVFNIGEGMPGRTFANGEPIWLCNAHTADSKVFSRSLLAKSAAVKTVVCFPFLGGVVEIGTTEHITEDMNVIQCVKTSFLEAPDPYATILPARSDYHIDNVLDPQQILGDEIYAPMFSTEPFPTASPSRTTNGFDQEHEQVADDHDSFMTERITGGASQVQSWQLMDDELSNCVHQSLNSSDCVSQTFVEGAAGRVAYGARKSRVQRLGQIQEQQRNVKTLSFDPRNDDVHYQSVISTIFKTNHQLILGPQFRNCDKQSSFTRWKKSSSSSSGTATVTAPSQGMLKKIIFDVPRVHQKEKLMLDSPEARDETGNHAVLEKKRREKLNERFMTLRKIIPSINKIDKVSILDDTIEYLQELERRVQELESCRESTDTETRGTMTMKRKKPCDAGERTSANCANNETGNGKKVSVNNVGEAEPADTGFTGLTDNLRIGSFGNEVVIELRCAWREGVLLEIMDVISDLHLDSHSVQSSTGDGLLCLTVNCKHKGSKIATPGMIKEALQRVAWIC

>AtbHLH051

MENSYDSSKWSDSTTPYMVSWSLQSESSDSDWNRFNLGFSSSSFGGNFPADDCVGGIEKAESLSRSHRLAEKRRRDRINSHLTALRKLVPNSDKLDKAALLATVIEQVKELKQKAAESPIFQDLPTEADEVTVQPETISDFESNTNTIIFKASFCCEDQPEAISEIIRVLTKLQLETIQAEIISVGGRMRINFILKDSNCNETTNIAASAKALKQSLCSALNRITSSSTTTSSVCRIRSKRQRWFLSSHYSHNE

>AtbHLH008

MPLFELFRLTKAKLESAQDRNPSPPVDEVVELVWENGQISTQSQSSRSRNIPPPQANSSRAREIGNGSKTTMVDEIPMSVPSLMTGLSQDDDFVPWLNHHPSLDGYCSDFLRDVSSPVTVNEQESDMAVNQTAFPLFQRRKDGNESAPAASSSQYNGFQSHSLYGSDRARDLPSQQTNPDRFTQTQEPLITSNKPSLVNFSHFLRPATFAKTTNNNLHDTKEKSPQSPPNVFQTRVLGAKDSEDKVLNESVASATPKDNQKACLISEDSCRKDQESEKAVVCSSVGSGNSLDGPSESPSLSLKRKHSNIQDIDCHSEDVEEESGDGRKEAGPSRTGLGSKRSRSAEVHNLSERRRRDRINEKMRALQELIPNCNKVDKASMLDEAIEYLKSLQLQVQIMSMASGYYLPPAVMFPPGMGHYPAAAAAMAMGMGMPYAMGLPDLSRGGSSVNHGPQFQVSGMQQQPVAMGIPRVSGGGIFAGSSTIGNGSTRDLSGSKDQTTTNNNSNLKPIKRKQGSSDQFCGSS

>AtbHLH054

MDVFVDGELESLLGMFNFDQCSSSKEERPRDELLGLSSLYNGHLHQHQHHNNVLSSDHHAFLLPDMFPFGAMPGGNLPAMLDSWDQSHHLQETSSLKRKLLDVENLCKTNSNCDVTRQELAKSKKKQRVSSESNTVDESNTNWVDGQSLSNSSDDEKASVTSVKGKTRATKGTATDPQSLYARKRREKINERLKTLQNLVPNGTKVDISTMLEEAVHYVKFLQLQIKLLSSDDLWMYAPLAYNGLDMGFHHNLLSRLM

>AtbHLH002

MDVFVDGELESLLGMFNFDQCSSSKEERPRDELLGLSSLYNGHLHQHQHHNNVLSSDHHAFLLPDMFPFGAMPGGNLPAMLDSWDQSHHLQETSSLKRKLLDVENLCKTNSNCDVTRQELAKSKKKQRVSSESNTVDESNTNWVDGQSLSNSSDDEKASVTSVKGKTRATKGTATDPQSLYARKRREKINERLKTLQNLVPNGTKVDISTMLEEAVHYVKFLQLQIKLLSSDDLWMYAPLAYNGLDMGFHHNLLSRLM

>AtbHLH062

MENELFMNAGVSHPPVMTSPSSSSAMLKWVSMETQPVDPSLSRNLFWEKSTEQSIFDSALSSLVSSPTPSNSNFSVGGVGGENVIMRELIGKLGNIGDIYGITASNGNSCYATPMSSPPPGSMMETKTTTPMAELSGDPGFAERAARFSCFGSRSFNSRTNSPFPINNEPPITTNEKMPRVSSSPVFKPLASHVPAGESSGELSRKRKTKSKQNSPSAVSSSKEIEEKEDSDPKRCKKSEENGDKTKSIDPYKDYIHVRARRGQATDSHSLAERVRREKISERMKLLQDLVPGCNKVTGKALMLDEIINYVQSLQRQVEFLSMKLSSVNTRLDFNMDALLSKDIFPSSNNLMHHQQVLQLDSSAETLLGDHHNKNLQLNPDISSNNVINPLETSETRSFISHLPTLAHFTDSISQYSTFSEDDLHSIIHMGFAQNRLQELNQGSSNQVPSHMKAEL

>AtbHLH065

MEQVFADWNFEDNFHMSTNKRSIRPEDELVELLWRDGQVVLQSQARREPSVQVQTHKQETLRKPNNIFLDNQETVQKPNYAALDDQETVSWIQYPPDDVIDPFESEFSSHFFSSIDHLGGPEKPRTIEETVKHEAQAMAPPKFRSSVITVGPSHCGSNQSTNIHQATTLPVSMSDRSKNVEERLDTSSGGSSGCSYGRNNKETVSGTSVTIDRKRKHVMDADQESVSQSDIGLTSTDDQTMGNKSSQRSGSTRRSRAAEVHNLSERRRRDRINERMKALQELIPHCSRTDKASILDEAIDYLKSLQMQLQVMWMGSGMAAAAAAAASPMMFPGVQSSPYINQMAMQSQMQLSQFPVMNRSAPQNHPGLVCQNPVQLQLQAQNQILSEQLARYMGGIPQMPPAGNQMQTVQQQPADMLGFGSPAGPQSQLSAPATTDSLHMGKIG

>AtbHLH029

MEGRVNALSNINDLELHNFLVDPNFDQFINLIRGDHQTIDENPVLDFDLGPLQNSPCFIDENQFIPTPVDDLFDELPDLDSNVAESFRSFDGDSVRAGGEEDEEDYNDGDDSSATTTNNDGTRKTKTDRSRTLISERRRRGRMKDKLYALRSLVPNITKMDKASIVGDAVLYVQELQSQAKKLKSDIAGLEASLNSTGGYQEHAPDAQKTQPFRGINPPASKKIIQMDVIQVEEKGFYVRLVCNKGEGVAPSLYKSLESLTSFQVQNSNLSSPSPDTYLLTYTLDGTCFEQSLNLPNLKLWITGSLLNQGFEFIKSFT

>AtbHLH091

MYEESSCFDPNSMVDNNGGFCAAETTFTVSHQFQPPLGSTTNSFDDDLKLPTMDEFSVFPSVISLPNSETQNQNISNNNHLINQMIQESNWGVSEDNSNFFMNTSHPNTTTTPIPDLLSLLHLPRCSMSLPSSDIMAGSCFTYDPLFHLNLPPQPPLIPSNDYSGYLLGIDTNTTTQRDESNVGDENNNAQFDSGIIEFSKEIRRKGRGKRKNKPFTTERERRCHLNERYEALKLLIPSPSKGDRASILQDGIDYINELRRRVSELKYLVERKRCGGRHKNNEVDDNNNNKNLDDHGNEDDDDDDENMEKKPESDVIDQCSSNNSLRCSWLQRKSKVTEVDVRIVDDEVTIKVVQKKKINCLLLVSKVLDQLQLDLHHVAGGQIGEHYSFLFNTKIYEGSTIYASAIANRVIEVVDKHYMASLPNSNY

>AtbHLH038

MCALVPSFFTNFGWPSTNQYESYYGAGDNLNNGTFLELTVPQTYEVTHHQNSLGVSVSSEGNEIDNNPVVVKKLNHNASERDRRKKINTLFSSLRSCLPASDQSKKLSIPETVSKSLKYIPELQQQVKRLIQKKEEILVRVSGQRDFELYDKQQPKAVASYLSTVSATRLGDNEVMVQVSSSKIHNFSISNVLGGIEEDGFVLVDVSSSRSQGERLFYTLHLQVENMDDYKINCEELSERMLYLYEKCENSFN

>AtbHLH045

MSHIAVERNRRRQMNEHLKSLRSLTPCFYIKRGDQASIIGGVIEFIKELQQLVQVLESKKRRKTLNRPSFPYDHQTIEPSSLGAATTRVPFSRIENVMTTSTFKEVGACCNSPHANVEAKISGSNVVLRVVSRRIVGQLVKIISVLEKLSFQVLHLNISSMEETVLYFFVVKIGLECHLSLEELTLEVQKSFVSDEVIVSTN

>AtbHLH035

MEDIVDQELSNYWEPSSFLQNEDFEYDRSWPLEEAISGSYDSSSPDGAASSPASKNIVSERNRRQKLNQRLFALRSVVPNITKMDKASIIKDAISYIEGLQYEEKKLEAEIRELESTPKSSLSFSKDFDRDLLVPVTSKKMKQLDSGSSTSLIEVLELKVTFMGERTMVVSVTCNKRTDTMVKLCEVFESLNLKILTSNLTSFSGMIFHTVFIEVSIFLSLSLLSL

>AtbHLH022

MGGGSRFQEPVRMSRRKQVTKEKEEDENFKSPNLEAERRRREKLHCRLMALRSHVPIVTNMTKASIVEDAITYIGELQNNVKNLLETFHEMEEAPPEIDEEQTDPMIKPEVETSDLNEEMKKLGIEENVQLCKIGERKFWLKIITEKRDGIFTKFMEVMRFLGFEIIDISLTTSNGAILISASVQTQELCDVEQTKDFLLEVMRSNP

>AtbHLH015

MHHFVPDFDTDDDYVNNHNSSLNHLPRKSITTMGEDDDLMELLWQNGQVVVQNQRLHTKKPSSSPPKLLPSMDPQQQPSSDQNLFIQEDEMTSWLHYPLRDDDFCSDLLFSAAPTATATATVSQVTAARPPVSSTNESRPPVRNFMNFSRLRGDFNNGRGGESGPLLSKAVVRESTQVSPSATPSAAASESGLTRRTDGTDSSAVAGGGAYNRKGKAVAMTAPAIEITGTSSSVVSKSEIEPEKTNVDDRKRKEREATTTDETESRSEETKQARVSTTSTKRSRAAEVHNLSERKRRDRINERMKALQELIPRCNKSDKASMLDEAIEYMKSLQLQIQMMSMGCGMMPMMYPGMQQYMPHMAMGMGMNQPIPPPSFMPFPNMLAAQRPLPTQTHMAGSGPQYPVHASDPSRVFVPNQQYDPTSGQPQYPAGYTDPYQQFRGLHPTQPPQFQNQATSYPSSSRVSSSKESEDHGNHTTG

>AtbHLH083

MALVNDHPNETNYLSKQNSSSSEDLSSPGLDQPDAAYAGGGGGGGSASSSSTMNSDHQQHQGFVFYPSGEDHHNSLMDFNGSSFLNFDHHESFPPPAISCGGSSGGGGFSFLEGNNMSYGFTNWNHQHHMDIISPRSTETPQGQKDWLYSDSTVVTTGSRNESLSPKSAGNKRSHTGESTQPSKKLSSGVTGKTKPKPTTSPKDPQSLAAKNRRERISERLKILQELVPNGTKVDLVTMLEKAISYVKFLQVQVKVLATDEFWPAQGGKAPDISQVKDAIDAILSSSQRDRNSNLITN

>AtbHLH162

MEPSHSNTGQSRSVDRKTVEKNRRMQMKSLYSELISLLPHHSSTEPLTLPDQLDEAANYIKKLQVNVEKKRERKRNLVATTTLEKLNSVGSSSVSSSVDVSVPRKLPKIEIQETGSIFHIFLVTSLEHKFMFCEIIRVLTEELGAEITHAGYSIVDDAVFHTLHCKVEEHDYGARSQIPERLEKIVNSVH

>AtbHLH046

METELTQLRKQESNNLNGVNGGFMAIDQFVPNDWNFDYLCFNNLLQEDDNIDHPSSSSLMNLISQPPPLLHQPPQPSSPLYDSPPLSSAFDYPFLEDIIHSSYSPPPLILPASQENTNNYSPLMEESKSFISIGETNKKRSNKKLEGQPSKNLMAERRRRKRLNDRLSLLRSIVPKITKMDRTSILGDAIDYMKELLDKINKLQEDEQELGSNSHLSTLITNESMVRNSLKFEVDQREVNTHIDICCPTKPGLVVSTVSTLETLGLEIEQCVISCFSDFSLQASCFEVGEQRYMVTSEATKQALIRNAGYGGRCL

>AtbHLH041

MMHLILSCSYLISMDGYYNEASEEPSSSSSSGSLARSLFHEYRQSVIPLQNGHVPSMAFMNNLPYVEIRPQESQRLAFNDTQRLFYQMKIEASLREWFPEDFNRKSSPANSDYLRPPHYPSSSSSSLSPNNISEYSSLLFPLIPKPSTTTEAVNVPVLPPLAPINMIHPQHQEPLFRNRQREEEAMTQAILAVLTGPSSPPSTSSSPQRKGRATAFKRYYSMISDRGRAPLPSVRKQSMMTRAMSFYNRLNINQRERFTRENATTHGEGSGGSGGGGRYTSGPSATQLQHMISERKRREKLNESFQALRSLLPPGTKKDKASVLSIAREQLSSLQGEISKLLERNREVEAKLAGEREIENDLRPEERFNVRIRHIPESTSRERTLDLRVVLRGDIIRVDDLMIRLLEFLKQINNVSLVSIEARTLARAEGDTSIVLVISLRLKIEGEWDESAFQEAVRRVVADLAH

>AtbHLH017

MNMSDLGWDDEDKSVVSAVLGHLASDFLRANSNSNQNLFLVMGTDDTLNKKLSSLVDWPNSENFSWNYAIFWQQTMSRSGQQVLGWGDGCCREPNEEEESKVVRSYNFNNMGAEEETWQDMRKRVLQKLHRLFGGSDEDNYALSLEKVTATEIFFLASMYFFFNHGEGGPGRCYSSGKHVWLSDAVNSESDYCFRSFMAKSAGIRTIVMVPTDAGVLELGSVWSLPENIGLVKSVQALFMRRVTQPVMVTSNTNMTGGIHKLFGQDLSGAHAYPKKLEVRRNLDERFTPQSWEGYNNNKGPTFGYTPQRDDVKVLENVNMVVDNNNYKTQIEFAGSSVAASSNPSTNTQQEKSESCTEKRPVSLLAGAGIVSVVDEKRPRKRGRKPANGREEPLNHVEAERQRREKLNQRFYALRSVVPNISKMDKASLLGDAISYIKELQEKVKIMEDERVGTDKSLSESNTITVEESPEVDIQAMNEEVVVRVISPLDSHPASRIIQAMRNSNVSLMEAKLSLAEDTMFHTFVIKSNNGSDPLTKEKLIAAFYPETSSTQPPLPSSSSQVSGDI

>AtbHLH128

MYQSSSSTSSSSQRSSLPGGGGLIRYGSAPGSFLNSVVDEVIGGGSSNARDFTGYQPSSDNFIGNFFTGAADSSSLRSDSTTCGVNNSSDGQKQLGNNNNNNSNKDIFLDRSYGGFNEISQQHKSNDIGGGNSSGSYSLARQRSSPADFFTYLASDKNNFSLNQPTSDYSPQGGSNGGRGHSRLKSQLSFTNHDSLARINEVNETPVHDGSGHSFSAASFGAATTDSWDDGSGSIGFTVTRPSKRSKDMDSGLFSQYSLPSDTSMNYMDNFMQLPEDSVPCKIRAKRGCATHPRSIAERERRTRISGKLKKLQDLVPNMDKQTSYSDMLDLAVQHIKGLQHQLQNLKKDQENCTCGCSEKPS

>AtbHLH085

MEAMGEWSNNLGGMYTYATEEADFMNQLLASYDHPGTGSSSGAAASGDHQGLYWNLGSHHNHLSLVSEAGSFCFSQESSSYSAGNSGYYTVVPPTVEENQNETMDFGMEDVTINTNSYLVGEETSECDVEKYSSGKTLMPLETVVENHDDEESLLQSEISVTTTKSLTGSKKRSRATSTDKNKRARVNKRAQKNVEMSGDNNEGEEEEGETKLKKRKNGAMMSRQNSSTTFCTEEESNCADQDGGGEDSSSKEDDPSKALNLNGKTRASRGAATDPQSLYARKRRERINERLRILQNLVPNGTKVDISTMLEEAVHYVKFLQLQIKLLSSDDLWMYAPIAFNGMDIGLSSPR

>AtbHLH116

MGLDGNNGGGVWLNGGGGEREENEEGSWGRNQEDGSSQFKPMLEGDWFSSNQPHPQDLQMLQNQPDFRYFGGFPFNPNDNLLLQHSIDSSSSCSPSQAFS

LDPSQQNQFLSTNNNKGCLLNVPSSANPFDNAFEFGSESGFLNQIHAPISMGFGSLTQLGNRDLSSVPDFLSARSLLAPESNNNNTMLCGGFTAPLELEG

FGSPANGGFVGNRAKVLKPLEVLASSGAQPTLFQKRAAMRQSSGSKMGNSESSGMRRFSDDGDMDETGIEVSGLNYESDEINESGKAAESVQIGGGGKGKKKGMPAKNLMAERRRRKKLNDRLYMLRSVVPKISKMDRASILGDAIDYLKELLQRINDLHNELESTPPGSLPPTSSSFHPLTPTPQTLSCRVKEELCPSSLPSPKGQQARVEVRLREGRAVNIHMFCGRRPGLLLATMKALDNLGLDVQQAVISCFNGFALDVFRAEQCQEGQEILPDQIKAVLFDTAGYAGMI

>AtbHLH033

MNSDGVWLDGSGESPEVNNGEAASWVRNPDEDWFNNPPPPQHTNQNDFRFNGGFPLNPSENLLLLLQQSIDSSSSSSPLLHPFTLDAASQQQQQQQQQQEQSFLATKACIVSLLNVPTINNNTFDDFGFDSGFLGQQFHGNHQSPNSMNFTGLNHSVPDFLPAPENSSGSCGLSPLFSNRAKVLKPLQVMASSGSQPTLFQKRAAMRQSSSSKMCNSESSSEMRKSSYEREIDDTSTGIIDISGLNYESDDHNTNNNKGKKKGMPAKNLMAERRRRKKLNDRLYMLRSVVPKISKMDRASILGDAIDYLKELLQRINDLHTELESTPPSSSSLHPLTPTPQTLSYRVKEELCPSSSLPSPKGQQPRVEVRLREGKAVNIHMFCGRRPGLLLSTMRALDNLGLDVQQAVISCFNGFALDVFRAEQCQEDHDVLPEQIKAVLLDTAGYAGLV

>AtbHLH087

MEGLESVYAQAMYGMTRESKIMEHQGSDLIWGGNELMARELCSSSSYHHQLINPNLSSCFMSDLGVLGEIQQQQHVGNRASSIDPSSLDCLLSATSNSNNTSTEDDEGISVLFSDCQTLWSFGGVSSAESENREITTETTTTIKPKPLKRNRGGDGGTTETTTTTTKPKSLKRNRGDETGSHFSLVHPQDDSEKGGFKLIYDENQSKSKKPRTEKERGGSSNISFQHSTCLSDNVEPDAEAIAQMKEMIYRAAAFRPVNFGLEIVEKPKRKNVKISTDPQTVAARQRRERISEKIRVLQTLVPGGTKMDTASMLDEAANYLKFLRAQVKALENLRPKLDQTNLSFSSAPTSFPLFHPSFLPLQNPNQIHHPEC

>AtbHLH030

MCAKKEEEEEEEEDSSEAMNNIQNYQNDLFFHQLISHHHHHHHDPSQSETLGASGNVGSGFTIFSQDSVSPIWSLPPPTSIQPPFDQFPPPSSSPASFYGSFFNRSRAHHQGLQFGYEGFGGATSAAHHHHEQLRILSEALGPVVQAGSGPFGLQAELGKMTAQEIMDAKALAASKSHSEAERRRRERINNHLAKLRSILPNTTKTDKASLLAEVIQHVKELKRETSVISETNLVPTESDELTVAFTEEEETGDGRFVIKASLCCEDRSDLLPDMIKTLKAMRLKTLKAEITTVGGRVKNVLFVTGEESSGEEVEEEYCIGTIEEALKAVMEKSNVEESSSSGNAKRQRMSSHNTITIVEQQQQYNQR

>AtbHLH013

MNIGRLVWNEDDKAIVASLLGKRALDYLLSNSVSNANLLMTLGSDENLQNKLSDLVERPNASNFSWNYAIFWQISRSKAGDLVLCWGDGYCREPKEGEKSEIVRILSMGREEETHQTMRKRVLQKLHDLFGGSEEENCALGLDRVTDTEMFLLSSMYFSFPRGEGGPGKCFASAKPVWLSDVVNSGSDYCVRSFLAKSAGIQTVVLVPTDLGVVELGSTSCLPESEDSILSIRSLFTSSLPPVRAVALPVTVAEKIDDNRTKIFGKDLHNSGFLQHHQHHQQQQQQPPQQQQHRQFREKLTVRKMDDRAPKRLDAYPNNGNRFMFSNPGTNNNTLLSPTWVQPENYTRPINVKEVPSTDEFKFLPLQQSSQRLLPPAQMQIDFSAASSRASENNSDGEGGGEWADAVGADESGNNRPRKRGRRPANGRAEALNHVEAERQRREKLNQRFYALRSVVPNISKMDKASLLGDAVSYINELHAKLKVMEAERERLGYSSNPPISLDSDINVQTSGEDVTVRINCPLESHPASRIFHAFEESKVEVINSNLEVSQDTVLHTFVVKSEELTKEKLISALSREQTNSVQSRTSSGR

>AtbHLH074

MGGESNEGGEMGFKHGDDESGGISRVGITSMPLYAKADPFFSSADWDPVVNAAAAGFSSSHYHPSMAMDNPGMSCFSHYQPGSVSGFAADMPASLLPFGDCGGGQIGHFLGSDKKGERLIRAGESSHEDHHQVSDDAVLGASPVGKRRLPEAESQWNKKAVEEFQEDPQRGNDQSQKKHKNDQSKETVNKESSQSEEAPKENYIHMRARRGQATNSHSLAERVRREKISERMRLLQELVPGCNKITGKAVMLDEIINYVQSLQQQVEFLSMKLATVNPEINIDIDRILAKDLLQSRDRNTPTLGLNPFAGFQGNIPNLSATTNPQYNPLPQTTLESELQNLYQMGFVSNPSTMSSFSPNGRLKPEL

>AtbHLH024

MISQREEREEKKQRVMGDKKLISSSSSSSVYDTRINHHLHHPPSSSDEISQFLRHIFDRSSPLPSYYSPATTTTTASLIGVHGSGDPHADNSRSLVSHHPPSDSVLMSKRVGDFSEVLIGGGSGSAAACFGFSGGGNNNNVQGNSSGTRVSSSSVGASGNETDEYDCESEEGGEAVVDEAPSSKSGPSSRSSSKRCRAAEVHNLSEKRRRSRINEKMKALQSLIPNSNKTDKASMLDEAIEYLKQLQLQVQMLTMRNGINLHPLCLPGTTLHPLQLSQIRPPEATNDPLLNHTNQFASTSNAPEMINTVASSYALEPSIRSHFGPFPLLTSPVEMSREGGLTHPRLNIGHSNANITGEQALFDGQPDLKDRIT

>AtbHLH096

MALEAVVYPQDPFSYISCKDFPFYDLYFQEEEDQDPQDTKNNIKLGQGQGHGFASNNYNGRTGDYSDDYNYNEEDLQWPRDLPYGSAVDTESQPPPSDVAAGGGRRKRRRTRSSKNKEEIENQRMTHIAVERNRRKQMNEYLAVLRSLMPPYYAQRGDQASIVGGAINYLKELEHHLQSMEPPVKTATEDTGAGHDQTKTTSASSSGPFSDFFAFPQYSNRPTSAAAAEGMAEIEVTMVESHASLKILAKKRPRQLLKLVSSIQSLRLTLLHLNVTTRDDSVLYSISVKVEEGSQLNTVEDIAAAVNQILRRIEEESSFS

>AtbHLH102

MRTGKGNQEEEDYGEEDFNSKREGPSSNTTVHSNRDSKENDKASAIRSKHSVTEQRRRSKINERFQILRELIPNSEQKRDTASFLLEVIDYVQYLQEKVQKYEGSYPGWSQEPTKLTPWRNNHWRVQSLGNHPVAINNGSGPGIPFPGKFEDNTVTSTPAIIAEPQIPIESDKARAITGISIESQPELDDKGLPPLQPILPMVQGEQANECPATSDGLGQSNDLVIEGGTISISSAYSHELLSSLTQALQNAGIDLSQAKLSVQIDLGKRANQGLTHEEPSSKNPLSYDTQGRDSSVEEESEHSHKRMKTL

>AtbHLH137

MATFSYFQNYPHSLLDPLLFPTPHSSINLTSFIDQNHLYPLPNISTVEDISFLEYNVDKTENSGSEKLANTTKTATTGSSSCDQLSHGPSAITNTGKTRGRKARNSNNSKEGVEGRKSKKQKRGSKEEPPTDYIHVRARRGQATDSHSLAERVRREKISERMRTLQNLVPGCDKVTGKALMLDEIINYVQTLQTQVEFLSMKLTSISPVVYDFGSDLDGLILQSEMGSPEVGTSFTNAMPTTTPIFPSLLDNSVVPTHAQVQEEGEERENFVDRSGFNNNNFCSFP

>AtbHLH066

MMNSSLLTPSSSSSSHIQTPSTTFDHEDFLDQIFSSAPWPSVVDDAHPLPSDGFHGHDVDSRNQPIMMMPLNDGSSVHALYNGFSVAGSLPNFQIPQGSGGGLMNQQGQTQTQTQPQASASTATGGTVAAPPQSRTKIRARRGQATDPHSIAERLRRERIAERMKALQELVPNGNKTDKASMLDEIIDYVKFLQLQVKVLSMSRLGGAASVSSQISEAGGSHGNASSAMVGGSQTAGNSNDSVTMTEHQVAKLMEEDMGSAMQYLQGKGLCLMPISLATAISTATCHSRNPLIPGAVADVGGPSPPNLSGMTIQSTSTKMGSGNGKLNGNGVTERSSSIAVKEAVSVSKA

>AtbHLH037

MDNSDILMNMMMQQMEKLPEHFSNSNPNPNPHNIMMLSESNTHPFFFNPTHSHLPFDQTMPHHQPGLNFRYAPSPSSSLPEKRGGCSDNANMAAMREMIFRIAVMQPIHIDPESVKPPKRKNVRISKDPQSVAARHRRERISERIRILQRLVPGGTKMDTASMLDEAIHYVKFLKKQVQSLEEHAVVNGGGMTAVAGGALAGTVGGGYGGKGCGIMRSDHHQMLGNAQILR

>AtbHLH006

MTDYRLQPTMNLWTTDDNASMMEAFMSSSDISTLWPPASTTTTTATTETTPTPAMEIPAQAGFNQETLQQRLQALIEGTHEGWTYAIFWQPSYDFSGASVLGWGDGYYKGEEDKANPRRRSSSPPFSTPADQEYRKKVLRELNSLISGGVAPSDDAVDEEVTDTEWFFLVSMTQSFACGAGLAGKAFATGNAVWVSGSDQLSGSGCERAKQGGVFGMHTIACIPSANGVVEVGSTEPIRQSSDLINKVRILFNFDGGAGDLSGLNWNLDPDQGENDPSMWINDPIGTPGSNEPGNGAPSSSSQLFSKSIQFENGSSSTITENPNLDPTPSPVHSQTQNPKFNNTFSRELNFSTSSSTLVKPRSGEILNFGDEGKRSSGNPDPSSYSGQTQFENKRKRSMVLNEDKVLSFGDKTAGESDHSDLEASVVKEVAVEKRPKKRGRKPANGREEPLNHVEAERQRREKLNQRFYALRAVVPNVSKMDKASLLGDAIAYINELKSKVVKTESEKLQIKNQLEEVKLELAGRKASASGGDMSSSCSSIKPVGMEIEVKIIGWDAMIRVESSKRNHPAARLMSALMDLELEVNHASMSVVNDLMIQQATVKMGFRIYTQEQLRASLISKIG

>AtbHLH092

MDNFFLGLSCQEENNFWDLIVADISGDRSVSVPIRSAFRSYMKDTELRMMSPKISSSKVNVKKRMVNLLRKNWEEKKNTVAPEKERSRRHMLKERTRREKQKQSYLALHSLLPFATKNDKNSIVEKAVDEIAKLQRLKKELVRRIKVIEEKSAKDGHDEMSETKVRVNLKEPLSGLDSMLEALHYLKSMGTKLKTVHANFSPQEFSATMTIETQIRGEEVEKRVERRLQETEWKLLFLPEASFYKDY

>AtbHLH059

MASNNPHDNLSDQTPSDDFFEQILGLPNFSASSAAGLSGVDGGLGGGAPPMMLQLGSGEEGSHMGGLGGSGPTGFHNQMFPLGLSLDQGKGPGFLRPEGGHGSGKRFSDDVVDNRCSSMKPVFHGQPMQQPPPSAPHQPTSIRPRVRARRGQATDPHSIAERLRRERIAERIRALQELVPTVNKTDRAAMIDEIVDYVKFLRLQVKVLSMSRLGGAGAVAPLVTDMPLSSSVEDETGEGGRTPQPAWEKWSNDGTERQVAKLMEENVGAAMQLLQSKALCMMPISLAMAIYHSQPPDTSSVVKPENNPPQ

>AtbHLH106

MQPETSDQMLYSFLAGNEVGGGGYCVSGDYMTTMQSLCGSSSSTSSYYPLAISGIGETMAQDRALAALRNHKEAERRRRERINSHLNKLRNVLSCNSKTDKATLLAKVVQRVRELKQQTLETSDSDQTLLPSETDEISVLHFGDYSNDGHIIFKASLCCEDRSDLLPDLMEILKSLNMKTLRAEMVTIGGRTRSVLVVAADKEMHGVESVHFLQNALKSLLERSSKSLMERSSGGGGGERSKRRRALDHIIMV

>AtbHLH053

MSMDCLSYFFNYDPPVQLQDCFIPEMDMIIPETDSFFFQSQPQLEFHQPLFQEEAPSQTHFDPFCDQFLSPQEIFLPNPKNEIFNETHDLDFFLPTPKRQRLVNSSYNCNTQNHFQSRNPNFFDPFGDTDFVPESCTFQEFRVPDFSLAFKVGRGDQDDSKKPTLSSQSIAARGRRRRIAEKTHELGKLIPGGNKLNTAEMFQAAAKYVKFLQSQVGILQLMQTTKKGSSNVQMETQYLLESQAIQEKLSTEEVCLVPCEMVQDLTTEETICRTPNISREINKLLSKHLAN

>AtbHLH049

MDLSAKDEFSAEKRNPDNYDSVNNPSGDWRVDSYPSENLISAGPASCSPSQMMDSFGQTLWYDPTSVQAVGYAGFNGGNASSSSFRGSIDRSLEMGWNLPNLLPPKGNGLFLPNASSFLPPSMAQFPADSGFIERAARFSLFSGGNFSDMVNQPLGNSEAIGLFLQGGGTMQGQCQSNELNVGEPHNDVSVAVKESTVRSSEQAKPNVPGSGNVSEDTQSSGGNGQKGRETSSNTKKRKRNGQKNSEAAQSHRSQQSEEEPDNNGDEKRNDEQSPNSPGKKSNSGKQQGKQSSDPPKDGYIHVRARRGQATNSHSLAERVRREKISERMKFLQDLVPGCNKVTGKAVMLDEIINYVQSLQRQVEFLSMKLATVNPQMDFNLEGLLAKDALQLRAGSSSTTPFPPNMSMAYPPLPHGFMQQTLSSIGRTITSPLSPMNGGFKRQETNGWEGDLQNVIHINYGAGDVTPDPQAAATASLPAANMKVEP

>AtbHLH063

MNGAIGGDLLLNFPDMSVLERQRAHLKYLNPTFDSPLAGFFADSSMITGGEMDSYLSTAGLNLPMMYGETTVEGDSRLSISPETTLGTGNFKKRKFDTETKDCNEKKKKMTMNRDDLVEEGEEEKSKITEQNNGSTKSIKKMKHKAKKEENNFSNDSSKVTKELEKTDYIHVRARRGQATDSHSIAERVRREKISERMKFLQDLVPGCDKITGKAGMLDEIINYVQSLQRQIEFLSMKLAIVNPRPDFDMDDIFAKEVASTPMTVVPSPEMVLSGYSHEMVHSGYSSEMVNSGYLHVNPMQQVNTSSDPLSCFNNGEAPSMWDSHVQNLYGNLGV

>AtbHLH089

MGGGGMFEEIGCFDPNAPAEMTAESSFSPSEPPPTITVIGSNSNSNCSLEDLSAFHLSPQDSSLPASASAYAHQLHINATPNCDHQFQSSMHQTLQDPSYAQQSNHWDNGYQDFVNLGPNHTTPDLLSLLQLPRSSLPPFANPSIQDIIMTTSSSVAAYDPLFHLNFPLQPPNGSFMGVDQDQTETNQGVNLMYDEENNNLDDGLNRKGRGSKKRKIFPTERERRVHFKDRFGDLKNLIPNPTKNDRASIVGEAIDYIKELLRTIDEFKLLVEKKRVKQRNREGDDVVDENFKAQSEVVEQCLINKKNNALRCSWLKRKSKFTDVDVRIIDDEVTIKIVQKKKINCLLFVSKVVDQLELDLHHVAGAQIGEHHSFLFNAKISEGSSVYASAIADRVMEVLKKQYMEALSANNGYHCYSSD

>AtbHLH082

MENGNGEGKGEFINQNNDFFLDSMSMLSSLPPCWDPSLPPPPPPPQSLFHALAVDAPFPDQFHHPQESGGPTMGSQEGLQPQGTVSTTSAPVVRQKPRVRARRGQATDPHSIAERLRRERIAERMKSLQELVPNTNKTDKASMLDEIIEYVRFLQLQVKVLSMSRLGGAGSVGPRLNGLSAEAGGRLNALTAPCNGLNGNGNATGSSNESLRSTEQRVAKLMEEDMGSAMQYLQGKGLCLMPISLATAISSSTTHSRGSLFNPISSAVAAEDSNVTATAVAAPEASSTMDDVSASKA

>AtbHLH095

MTNAQELGQEGFMWGISNSDDSGGGCKRIEKEPLPSHPSHPSPEIQTTTVKKGKKRTKRNDKNHEEESPDHEIHIWTERERRKKMRDMFSKLHALLPQLPPKADKSTIVDEAVSSIKSLEQTLQKLEMQKLEKLQYSSASTNTTPTTTFAYAPSSSSSPTALLTPISNHPIDATATDSYPRAAFLADQVSSSSAAAANLPYPCNDPIVNFDTWSSRNVVLTICGNEAFFNLCVPKHKPGVFTSVCYLFEKYNMEVLFANVSSNVFWSTYVIQAQVNPSCENQLLGNGLGVVDVFKQVSQELVLYFSSL

>AtbHLH086

MSLINEHCNERNYISTPNSSEDLSSPQNCGLDEGASASSSSTINSDHQNNQGFVFYPSGETIEDHNSLMDFNASSFFTFDNHRSLISPVTNGGAFPVVDGNMSYSYDGWSHHQVDSISPRVIKTPNSFETTSSFGLTSNSMSKPATNHGNGDWLYSGSTIVNIGSRHESTSPKLAGNKRPFTGENTQLSKKPSSGTNGKIKPKATTSPKDPQSLAAKNRRERISERLKVLQELVPNGTKVDLVTMLEKAIGYVKFLQVQVKVLAADEFWPAQGGKAPDISQVKEAIDAILSSSQRDSNSTRETSIAE

>AtbHLH098

MQEIIPDFLEECEFVDTSLAGDDLFAILESLEGAGEISPTAASTPKDGTTSSKELVKDQDYENSSPKRKKQRLETRKEEDEEEEDGDGEAEEDNKQDGQQKMSHVTVERNRRKQMNEHLTVLRSLMPCFYVKRGDQASIIGGVVEYISELQQVLQSLEAKKQRKTYAEVLSPRVVPSPRPSPPVLSPRKPPLSPRINHHQIHHHLLLPPISPRTPQPTSPYRAIPPQLPLIPQPPLRSYSSLASCSSLGDPPPYSPASSSSSPSVSSNHESSVINELVANSKSALADVEVKFSGANVLLKTVSHKIPGQVMKIIAALEDLALEILQVNINTVDETMLNSFTIKIGIECQLSAEELAQQIQQTFC

>AtbHLH130

MDSNNHLYDPNPTGSGLLRFRSAPSSVLAAFVDDDKIGFDSDRLLSRFVTSNGVNGDLGSPKFEDKSPVSLTNTSVSYAATLPPPPQLEPSSFLGLPPHYPRQSKGIMNSVGLDQFLGINNHHTKPVESNLLRQSSSPAGMFTNLSDQNGYGSMRNLMNYEEDEESPSNSNGLRRHCSLSSRPPSSLGMLSQIPEIAPETNFPYSHWNDPSSFIDNLSSLKREAEDDGKLFLGAQNGESGNRMQLLSHHLSLPKSSSTASDMVSVDKYLQLQDSVPCKIRAKRGCATHPRSIAERVRRTRISERMRKLQELVPNMDKQTNTSDMLDLAVDYIKDLQRQYKILNDNRANCKCMNKEKKSI

>AtbHLH040

MENGMYKKKGVCDSCVSSKSRSNHSPKRSMMEPQPHHLLMDWNKANDLLTQEHAAFLNDPHHLMLDPPPETLIHLDEDEEYDEDMDAMKEMQYMIAVMQPVDIDPATVPKPNRRNVRISDDPQTVVARRRRERISEKIRILKRIVPGGAKMDTASMLDEAIRYTKFLKRQVRILQPHSQIGAPMANPSYLCYYHNSQP

>AtbHLH161

MSSRKSRSRQTGASMITDEQINDLVLQLHRLLPELANNRRSGKVSASRVLQETCSYIRNLSKEVDDLSERLSQLLESTDSAQAALIRSLLMQ

>AtbHLH019

MDEDFFLPDFSLVDIDFDFNIYEENNLSPDESLSNSRRADQSSKFDHQMHFECLREKPKAAVKPMMKINNKQQLISFDFSSNVISSPAAEEIIMDKLVGRGTKRKTCSHGTRSPVLAKEHVLAERKRREKLSEKFIALSALLPGLKKADKVTILDDAISRMKQLQEQLRTLKEEKEATRQMESMILVKKSKVFFDEEPNLSCSPSVHIEFDQALPEIEAKISQNDILIRILCEKSKGCMINILNTIENFQLRIENSIVLPFGDSTLDITVLAQMDKDFSMSILKDLVRNLRLAMV

>AtbHLH021

MESNMQNLLEKLRPLVGARAWDYCVLWRLNEDQRFVKWMGCCCGGTELIAENGTEEFSYGGCRDVMFHHPRTKSCEFLSHLPASIPLDSGIYAETLLTNQTGWLSESSEPSFMQETICTRVLIPIPGGLVELFATRHVAEDQNVVDFVMGHCNMLMDDSVTINMMVADEVESKPYGMLSGDIQQKGSKEEDMMNLPSSYDISADQIRLNFLPQMSDYETQHLKMKSDYHHQALGYLPENGNKEMMGMNPFNTVEEDGIPVIGEPSLLVNEQQVVNDKDMNENGRVDSGSDCSDQIDDEDDPKYKKKSGKGSQAKNLMAERRRRKKLNDRLYALRSLVPRITKLDRASILGDAINYVKELQNEAKELQDELEENSETEDGSNRPQGGMSLNGTVVTGFHPGLSCNSNVPSVKQDVDLENSNDKGQEMEPQVDVAQLDGREFFVKVICEYKPGGFTRLMEALDSLGLEVTNANTTRYLSLVSNVFKVEKNDNEMVQAEHVRNSLLEITRNTSRGWQDDQMATGSMQNEKNEVDYQHYDDHQHHNGHHHPFDHQMNQSAHHHHHHQHINHYHNQ

>ppa024966m

MLALSPPVFSTVGWPSEDPIGYDQNYFYRDSSTDQTAESFLHILPSQLPQFELDLSTTISGDNSSGSTMAKKLNHNASER

DRRKKINSLYSSMRSLLPADQAKKLSIPDTISRVLKYIPELQKQVEGLIRKREELLSRASNQEDVMREEKDMKSTARSSS

LPAVSTYRLSDRELAIQISTLKTHNNLLSEILLNLEEEGLQVLNASFLESSGERVFYNLHLQVERSYRLECENLSEKLMS

FYA

>ppa010295m

MGSQDPVPAADKANVTEASVRGACSGKKIQGKIPKRIHKAEREKQKREHFNELFLELADALELNQQNNGKASILCEATRL

LKDLIGQIEYLQKENASLLSESNYMTIEKNELREDNSALETQNEKLHSEIEDKVVQSKPDLNAALPCAELRPEVASHFTG

TSVSLPTQDPSLQQAPAVFVMPLCPDLQSYPLPDAAQLTSNTTSHVSKPHARYPTSVDSWPSQLLGEKPTAGKESRQLGD

SSNICRSRETDPDNM

>ppa016095m

MLALSPPLFSTIAWPSEDLLSHDQNYFYRDSSIDQSAESFLHILPSHQPQVDLDRSTTISGDYSGVSTMAKKLNHNASER

DRRKKMNSLYSSLRSLLPVDQGKKLSIPNTISHVLKYIPELQKLVEGLIRKRGELLSRACKQEAAMHEEKRIKSTARSSS

SAVSTYRLSDRAVAIQISTLKTHNNLLSEILLNLEEEGLQVLNASSFQSSGGRVFYNIHLQVERADRLECKNLSEKLMSF

YA

>ppa002645m

MANGTQNHERVPENLRKQFAVAVRSIKWSYAIFWSLSTSQQGVLEWCEGYYNGDIKTRKTVEGVELKTDKMGLERNAQLR

ELYKSLLEGETEPQAKAPSAALNPEDLSDAEWYYLLCMSFVFNPGEGLPGRALANGQTIWLCDAQYADSKVFSRSLLAKS

ASIQTVVCFPYLGGVVELGVTELVPEDLSLIQHIKASLLDFSKPDCSEKSSSAPHKADDDSDQVLAKVDHEIVDTLALEN

LYSPSEEIKFDPMGINDLHGNYEEFNMDSPEECSNGCEHNHQTEDSFMPEGINDGASQVQSWHFMDEDFSIGVQDSMNSS

DCISEAFVNKKRAQSSPRHESVNRNHLKELENLNDTKFSSLDLGPADDHIHYTRTLSNILGSSTRLTENPCSCDGDCKSS

FVTWKKGVVDNCRPTVHQKILKKILFTVPLMCGASSQNTIQDGLSKLQSDDIHKGHVMPDKLKENEKLLVLRSMVPSISE

VDKASVLDDTIKYLKELEARAEEMESCMDTVEAIARRKYLDRAEKTSDNYDKIKMDNVKKPWLNKRKACDIDETDPDLNR

LVPRESLPLDVKVILKEQEVLIEMRCPYREYILLDIMDAINNLYLDAHSVQSSTLDGVLTLSLTSKFRGAAVAPVGMIKQ

ALWKIAGKC

>ppa027182m

MEDIISPCSSPKFGQENSATFHQRLQFIVQNRPEWWAYSIFWQASKDNNDDQVVLSWAGGHFKSSRDFASKRSNKMTNNY

QPKFGFSSVERKNVNNREVEVLFDEEDMVDLDTSLVDHGVGDVTDSEWFYFYTVSLTQSFAAGHGNNSILDRAFCSGGFV

WLAGDHELQFYECERVKEARMHGIQTLVCVATPCGVLELASLDVIKEDWGLVHLSKSLFESNNNRVSKQGSRDGNVLVSL

LENEMFSGGQKDLTRQGGSSSDSPSDSVGNFTSANAKNIRLKKRGRSSNHGTGRESSTHVEAERQRREKLNHRFYVLRSV

VPNVSKMDRSSLLADAVAYINQLKEKVEELEAKIQAQPQNPKVGHVSNLDHHHHHNSQSTGSIVDHHSSSYNINKAGAAL

EVDVKILGSEAMIRVQCPDQDYPYAKLMNALKALGFQVYHASISSVKELMIQDVVARVPYGFNSEEAVKMGIIKRWYN

**MYB**

>AtMYB27

MDFKKEETLRRGPWLEEEDERLVKVISLLGERRWDSLAIVSGLKRSGKSCRLRWMNYLNPTLKRGPMSQEEERIIFQLHALWGNKWSKIARRLPGRTDNEIKNYWRTHYRKKQEAQNYGKLFEWRGNTGEELLHKYKETEITRTKTTSQEHGFVEVVSMESGKEANGGVGGRESFGVMKSPYENRISDWISEISTDQSEANLSEDHSSNSCSENNINIGTWWFQETRDFEEFSCSLWS

>AtMYB121

MLDWGVQGHHQKHDHDIYQQQHQQQGCRKGPWTLEEDKLLAEYVTSHGEGRWSTVAKCAGLNRSGKSCRLRWVNYLRPGLKRGQITPQEEGIILELHSLWGNKWSTIARYLPGRTDNEIKNYWRTHYKKNQKSSSKQDKVKKSLSRKQQQVDLKPQPQAQSENHQSQLVSQDHMNIDNDHNIASSLYYPTSVFDDKLYMPQSVATTSSDHSMIDEGHLWGSLWNLDEDDPHSFGGGSGQGTAADIDEKFPDSGIEAPSCGSGDYSYTGVYMGGYIF

>AtMYB79

MVEEVWRKGPWTAEEDRLLIEYVRVHGEGRWNSVSKLAGLKRNGKSCRLRWVNYLRPDLKRGQITPHEESIILELHAKWGNRWSTIARSLPGRTDNEIKNYWRTHFKKKAKPTTNNAEKIKSRLLKRQHFKEQREIELQQEQQLFQFDQLGMKKIISLLEENNSSSSSDGGGDVFYYPDQITHSSKPFGYNSNSLEEQLQGRFSPVNIPDANTMNEDNAIWDGFWNMDVVNGHGGNLGVVAATAACGPRKPYFHNLVIPFC

>AtMYB71

MSLWGGMGGGWGMVEEGWRKGPWTAEEDRLLIDYVQLHGEGRWNSVARLAGLKRNGKSCRLRWVNYLRPDLKRGQITPHEETIILELHAKWGNRWSTIARSLPGRTDNEIKNYWRTHFKKKTKSPTNSAEKTKNRILKRQQFQQQRQMELQQEQQLLQFNQIDMKKIMSLLDDDNNNGDNTFSSSSSGESGALYVPHQITHSTTTSGCEPNSNGYYPVVPVTIPEANVNEDNAIWDGLWNLDFEGQGSFGGAACAPRKHYFQNMVIPFC

>AtMYB59

MGFCSESFRFEGGGRNIRIGLNRTGKSCRLRWVNYLHPGLKRGKMTPQEERLVLELHAKWGNRWSKIARKLPGRTDNEIKNYWRTHMRKKAQEKKRPMSPTSSSSNCCSSSMTTTTSQDTGGSNGKMNQECEDGYYSMDDIWREIDQSGANVIKPVKDNYYSEQSCYLNFPPLASPTWESSLESIWNMDADESKMSSFAIDQFPLSFEHGSGRL

>AtMYB48

MTPQEERLVLELHAKWGNRWSKIARKLPGRTDNEIKNYWRTHMRKKAQEKKRPVSPTSSFSNCSSSSVTTTTTNTQDTSCHSRKSSGEVSFYDTGGSRSTREMNQENEDVYSLDDIWREIDHSAVNIIKPVKDIYSEQSHCLSYPNLASPSWESSLDSIWNMDADKSKISSYFANDQFPFCFQHSRSPWSSG

>AtMYB57

METTMKKKGRVKATITSQKEEEGTVRKGPWTMEEDFILFNYILNHGEGLWNSVAKASGLKRTGKSCRLRWLNYLRPDVRRGNITEEEQLLIIQLHAKLGNRWSKIAKHLPGRTDNEIKNFWRTKIQRHMKVSSENMMNHQHHCSGNSQSSGMTTQGSSGKAIDTAESFSQAKTTTFNVVEQQSNENYWNVEDLWPVHLLNGDHHVI

>AtMYB24

MEKRESSGGSGSGDAEVRKGPWTMEEDLILINYIANHGEGVWNSLAKSAGLKRTGKSCRLRWLNYLRPDVRRGNITPEEQLTIMELHAKWGNRWSKIAKHLPGRTDNEIKNFWRTKIQKYIIKSGETTTVGSQSSEFINHHATTSHVMNDTQETMDMYSPTTSYQHASNINQQLNYGNYVPESGSIMMPLSVDQSEQNYWSVDDLWPMNIYNGN

>AtMYB21

MEKRGGGSSGGSGSSAEAEVRKGPWTMEEDLILINYIANHGDGVWNSLAKSAGLKRTGKSCRLRWLNYLRPDVRRGNITPEEQLIIMELHAKWGNRWSKIAKHLPGRTDNEIKNFWRTRIQKYIKQSDVTTTSSVGSHHSSEINDQAASTSSHNVFCTQDQAMETYSPTPTSYQHTNMEFNYGNYSAAAVTATVDYPVPMTVDDQTGENYWGMDDIWSSMHLLNGN

>AtMYB116

MSNITKKKCNGNEEGAEQRKGPWTLEEDTLLTNYISHNGEGRWNLLAKSSGLKRAGKSCRLRWLNYLKPDIKRGNLTPQEQLLILELHSKWGNRWSKISKYLPGRTDNDIKNYWRTRVQKQARQLNIDSNSHKFIEVVRSFWFPRLINEIKDNSYTNNIKANAPDLLGPILRDSKDLGFNNMDCSTSMSEDLKKTSQFMDFSDLETTMSLEGSRGGSSQCVSEVYSSFPCLEEEYMVAVMGSSDISALHDCHVADSKYEDDVTQDLMWNMDDIWQFNEYAHFN

>AtMYB62

MENSMKKKKSFKESEDEELRRGPWTLEEDTLLTNYILHNGEGRWNHVAKCAGLKRTGKSCRLRWLNYLKPDIRRGNLTPQEQLLILELHSKWGNRWSKIAQYLPGRTDNEIKNYWRTRVQKQARQLNIESNSDKFFDAVRSFWVPRLIEKMEQNSSTTTTYCCPQNNNNNSLLLPSQSHDSLSMQKDIDYSGFSNIDGSSSTSTCMSHLTTVPHFMDQSNTNIIDGSMCFHEGNVQEFGGYVPGMEDYMVNSDISMECHVADGYSAYEDVTQDPMWNVDDIWQFRE

>AtMYB2

MEDYERINSNSPTHEEDSDVRKGPWTEEEDAILVNFVSIHGDARWNHIARSSGLKRTGKSCRLRWLNYLRPDVRRGNITLEEQFMILKLHSLWGNRWSKIAQYLPGRTDNEIKNYWRTRVQKQAKHLRCDVNSNLFKETMRNVWMPRLVERINAQSLPTTCEQVESMITDPSQPVNEPSPVEPGFVQFSQNHHQQFVPATELSATSSNSPAETFSDVRGGVVNGSGYDPSGQTGFGEFNDWGCVGGDNMWTDEESFWFLQDQFCPDTTSYSYN

>AtMYB112

MNISRTEFANCKTLINHKEEVEEVEKKMEIEIRRGPWTVEEDMKLVSYISLHGEGRWNSLSRSAGLNRTGKSCRLRWLNYLRPDIRRGDISLQEQFIILELHSRWGNRWSKIAQHLPGRTDNEIKNYWRTRVQKHAKLLKCDVNSKQFKDTIKHLWMPRLIERIAATQSVQFTSNHYSPENSSVATATSSTSSSEAVRSSFYGGDQVEFGTLDHMTNGGYWFNGGDTFETLCSFDELNKWLIQ

>AtMYB108

MDEKGRSLKNNNMEDEMDLKRGPWTAEEDFKLMNYIATNGEGRWNSLSRCAGLQRTGKSCRLRWLNYLRPDVRRGNITLEEQLLILELHSRWGNRWSKIAQYLPGRTDNEIKNYWRTRVQKHAKQLKCDVNSQQFKDTMKYLWMPRLVERIQSASASSAAAATTTTTTTTGSAGTSSCITTSNNQFMNYDYNNNNMGQQFGVMSNNDYITPENSSVAVSPASDLTEYYSAPNPNPEYYSGQMGNSYYPDQNLVSSQLLPDNYFDYSGLLDEDLTAMQEQSNLSWFENINGAASSSDSLWNIGETDEEFWFLQQQQQFNNNGSF

>AtMYB78

MGDKGRSLKINKNMEEFTKVEEEMDVRRGPWTVEEDLELINYIASHGEGRWNSLARCAELKRTGKSCRLRWLNYLRPDVRRGNITLEEQLLILELHTRWGNRWSKIAQYLPGRTDNEIKNYWRTRVQKHAKQLKCDVNSQQFKDTMKYLWMPRLVERIQAASIGSVSMSSCVTTSSDQFVINNNNTNNVDNLALMSNPNGYITPDNSSVAVSPVSDLTECQVSSEVWKIGQDENLVDPKMTSPNYMDNSSGLLNGDFTKMQDQSDLNWFENINGMVPNYSDSFWNIGNDEDFWLLQQHQQVHDNGSF

>AtMYB86

MGRHSCCFKQKLRKGLWSPEEDEKLLNYITRHGHGCWSSVPKLAGLQRCGKSCRLRWINYLRPDLKRGAFSQDEESLIIELHAALGNRWSQIATRLPGRTDNEIKNFWNSCLKKKLRRKGIDPTTHKPLITNELQSLNVIDQKLTSSEVVKSTGSINNLHDQSMVVSSQQGPWWFPANTTTTNQNSAFCFSSSNTTTVSDQIVSLISSMSTSSSPTPMTSNFSPAPNNWEQLNYCNTVPSQSNSIYSAFFGNQYTEASQTMNNNNPLVDQHHHHQDMKSWASEILHYTEHNQSSETVIEAEVKPDIANYYWRSASSSSSPNQEAATLLHDANVEVYGKNLQKLNNMVFDQSL

>AtMYB55

MGRHSCCYKQKLRKGLWSPEEDEKLLRYITKYGHGCWSSVPKQAGLQRCGKSCRLRWINYLRPDLKRGAFSQDEENLIIELHAVLGNRWSQIAAQLPGRTDNEIKNLWNSCLKKKLRLRGIDPVTHKLLTEIETGTDDKTKPVEKSQQTYLVETDGSSSTTTCSTNQNNNTDHLYTGNFGFQRLSLENGSRIAAGSDLGIWIPQTGRNHHHHVDETIPSAVVLPGSMFSSGLTGYRSSNLGLIELENSFSTGPMMTEHQQIQESNYNNSTFFGNGNLNWGLTMEENQNPFTISNHSNSSLYSDIKSETNFFGTEATNVGMWPCNQLQPQQHAYGHI

>AtMYB61

MGRHSCCYKQKLRKGLWSPEEDEKLLTHITNHGHGCWSSVPKLAGLQRCGKSCRLRWINYLRPDLKRGAFSPEEENLIVELHAVLGNRWSQIASRLPGRTDNEIKNLWNSSIKKKLKQRGIDPNTHKPISEVESFSDKDKPTTSNNKRSGNDHKSPSSSSATNQDFFLERPSDLSDYFGFQKLNFNSNLGLSVTTDSSLCSMIPPQFSPGNMVGSVLQTPVCVKPSISLPPDNNSSSPISGGDHVKLAAPNWEFQTNNNNTSNFFDNGGFSWSIPNSSTSSSQVKPNHNFEEIKWSEYLNTPFFIGSTVQSQTSQPIYIKSETDYLANVSNMTDPWSQNENLGTTETSDVFSKDLQRMAVSFGQSL

>AtMYB50

MKRHSCCYKQKLRKGLWSPEEDEKLLNYITKHGHGCWSSVPKLAGLERCGKSCRLRWINYLRPDLKRGAFSSEEQNLIVELHAVLGNRWSQIAARLPGRTDNEIKNLWNSCIKKKLMKKGIDPITHKPLSEKEESNSVSLRNSLSSMIPTQFNIDDGSVSNAGFDTQVCVKPSIILLPPPNNTSSTVSGQDHVNVSEPNWESNSGTTSHLNNPGMEEMKWSEEYLNESLFSTQVYVKSETDFNSNIAFPWSQSQACDVFPKDLQRMAFSFGGQTL

>AtMYB26

MGHHSCCNKQKVKRGLWSPEEDEKLINYINSYGHGCWSSVPKHAGLQRCGKSCRLRWINYLRPDLKRGSFSPQEAALIIELHSILGNRWAQIAKHLPGRTDNEVKNFWNSSIKKKLMSHHHHGHHHHHLSSMASLLTNLPYHNGFNPTTVDDESSRFMSNIITNTNPNFITPSHLSLPSPHVMTPLMFPTSREGDFKFLTTNNPNQSHHHDNNHYNNLDILSPTPTINNHHQPSLSSCPHDNNLQWPALPDFPASTISGFQETLQDYDDANKLNVFVTPFNDNAKKLLCGEVLEGKVLSSSSPISQDHGLFLPTTYNFQMTSTSDHQHHHRVDSYINHMIIPSSSSSSPISCGQYVIT

>AtMYB67

MREKWEMKRDEMGHRCCGKHKVKRGLWSPEEDEKLLRYITTHGHPSWSSVPKLAGLQRCGKSCRLRWINYLRPDLRRGSFNEEEEQIIIDVHRILGNKWAQIAKHLPGRTDNEVKNFWNSCIKKKLLSQGLDPSTHNLMPSHKRSSSSNNNNIPKPNKTTSIMKNPTDLDQSTTAFSITNINPPTSTKPNKLKSPNQTTIPSQTVIPINDNMSSTQTMIPINDPMSSLLDDENMIPHWSDVDGMAIHEAPMLPSDKAVVGVDDDDLNMDILFNTPSSSAFDPDFASIFSSAMSIDFNPMDDLGSWTF

>AtMYB103

MGHHSCCNQQKVKRGLWSPEEDEKLIRYITTHGYGCWSEVPEKAGLQRCGKSCRLRWINYLRPDIRRGRFSPEEEKLIISLHGVVGNRWAHIASHLPGRTDNEIKNYWNSWIKKKIRKPHHHYSRHQPSVTTVTLNADTTSIATTIEASTTTTSTIDNLHFDGFTDSPNQLNFTNDQETNIKIQETFFSHKPPLFMVDTTLPILEGMFSENIITNNNKNNDHDDTQRGGRENVCEQAFLTTNTEEWDMNLRQQEPFQVPTLASHVFNNSSNSNIDTVISYNLPALIEGNVDNIVHNENSNVQDGEMASTFECLKRQELSYDQWDDSQQCSNFFFWDNLNINVEGSSLVGNQDPSMNLGSSALSSSFPSSF

>AtMYB83

MMMRKPDITTIRDKGKPNHACGGNNNKPKLRKGLWSPDEDEKLIRYMLTNGQGCWSDIARNAGLLRCGKSCRLRWINYLRPDLKRGSFSPQEEDLIFHLHSILGNRWSQIATRLPGRTDNEIKNFWNSTLKKRLKNNSNNNTSSGSSPNNSNSNSLDPRDQHVDMGGNSTSLMDDYHHDENMMTVGNTMRMDSSSPFNVGPMVNSVGLNQLYDPLMISVPDNGYHQMGNTVNVFSVNGLGDYGNTILDPISKRVSVEGDDWFIPPSENTNVIACSTSNNLNLQALDPCFNSKNLCHSESFKVGNVLGIENGSWEIENPKIGDWDLDGLIDNNSSFPFLDFQVD

>AtMYB46

MRKPEVAIAASTHQVKKMKKGLWSPEEDSKLMQYMLSNGQGCWSDVAKNAGLQRCGKSCRLRWINYLRPDLKRGAFSPQEEDLIIRFHSILGNRWSQIAARLPGRTDNEIKNFWNSTIKKRLKKMSDTSNLINNSSSSPNTASDSSSNSASSLDIKDIIGSFMSLQEQGFVNPSLTHIQTNNPFPTGNMISHPCNDDFTPYVDGIYGVNAGVQGELYFPPLECEEGDWYNANINNHLDELNTNGSGNAPEGMRPVEEFWDLDQLMNTEVPSFYFNFKQSI

>AtMYB45

MVFKSEKSNREMKSKEKQRKGLWSPEEDEKLRSHVLKYGHGCWSTIPLQAGLQRNGKSCRLRWVNYLRPGLKKSLFTKQEETILLSLHSMLGNKWSQISKFLPGRTDNEIKNYWHSNLKKGVTLKQHETTKKHQTPLITNSLEALQSSTERSSSSINVGETSNAQTSSFSPNLVFSEWLDHSLLMDQSPQKSSYVQNLVLPEERGFIGPCGPRYLGNDSLPDFVPNSEFLLDDEISSEIEFCTSFSDNFLFDGLINELRPM

>AtMYB19

MTKSGERPKQRQRKGLWSPEEDQKLKSFILSRGHACWTTVPILAGLQRNGKSCRLRWINYLRPGLKRGSFSEEEEETILTLHSSLGNKWSRIAKYLPGRTDNEIKNYWHSYLKKRWLKSQPQLKSQISDLTESPSSLLSCGKRNLETETLDHVISFQKFSENPTSSPSKESNNNMIMNNSNNLPKLFFSEWISSSNPHIDYSSAFTDSKHINETQDQINEEEVMMINNNNYSSLEDVMLRTDFLQPDHEYANYYSSGDFFINSDQNYV

>AtMYB18

MAKTKYGERHRKGLWSPEEDEKLRSFILSYGHSCWTTVPIKAGLQRNGKSCRLRWINYLRPGLKRDMISAEEEETILTFHSSLGNKWSQIAKFLPGRTDNEIKNYWHSHLKKKWLKSQSLQDAKSISPPSSSSSSLVACGKRNPETLISNHVFSFQRLLENKSSSPSQESNGNNSHQCSSAPEIPRLFFSEWLSSSYPHTDYSSEFTDSKHSQAPNVEETLSAYEEMGDVDQFHYNEMMINNSNWTLNDIVFGSKCKKQEHHIYREASDCNSSAEFFSPSTTT

>AtMYB34

MVRTPCCKEEGIKKGAWTPEEDQKLIAYLHLHGEGGWRTLPEKAGLKRCGKSCRLRWANYLRPDIKRGEFSPEEDDTIIKLHALKGNKWAAIATSLAGRTDNEIKNYWNTNLKKRLKQKGIDAITHKPINSTGQTGFEPKVNKPVYSSGSARLLNRVASKYAVELNRDLLTGIISGNSTVAEDSQNSGDVDSPTSTLLNKMAATSVLINTTTTYSGFSDNCSFTDEFNEFFNNEEISDIYTTVDNFGFMEELKSILSYGDASAGVIENSPEVNVADAMEFIDSWNEDDNMVGVFV

>AtMYB122

MVRTPCCRAEGLKKGAWTQEEDQKLIAYVQRHGEGGWRTLPDKAGLKRCGKSCRLRWANYLRPDIKRGEFSQDEEDSIINLHAIHGNKWSAIARKIPRRTDNEIKNHWNTHIKKCLVKKGIDPLTHKSLLDGAGKSSDHSAHPEKSSVHDDKDDQNSNNKKLSGSSSARFLNRVANRFGHRINHNVLSDIIGSNGLLTSHTTPTTSVSEGERSTSSSSTHTSSNLPINRSITVDATSLSSSTFSDSPDPCLYEEIVGDIEDMTRFSSRCLSHVLSHEDLLMSVESCLENTSFMREITMIFQEDKIETTSFNDSYVTPINEVDDSCEGIDNYFG

>AtMYB51

MVRTPCCKAELGLKKGAWTPEEDQKLLSYLNRHGEGGWRTLPEKAGLKRCGKSCRLRWANYLRPDIKRGEFTEDEERSIISLHALHGNKWSAIARGLPGRTDNEIKNYWNTHIKKRLIKKGIDPVTHKGITSGTDKSENLPEKQNVNLTTSDHDLDNDKAKKNNKNFGLSSASFLNKVANRFGKRINQSVLSEIIGSGGPLASTSHTTNTTTTSVSVDSESVKSTSSSFAPTSNLLCHGTVATTPVSSNFDVDGNVNLTCSSSTFSDSSVNNPLMYCDNFVGNNNVDDEDTIGFSTFLNDEDFMMLEESCVENTAFMKELTRFLHEDENDVVDVTPVYERQDLFDEIDNYFG

>AtMYB28

MSRKPCCVGEGLKKGAWTTEEDKKLISYIHDHGEGGWRDIPQKAGLKRCGKSCRLRWTNYLKPEIKRGEFSSEEEQIIIMLHASRGNKWSVIARHLPRRTDNEIKNYWNTHLKKRLMEQGIDPVTHKPLASSSNPTVDENLNSPNASSSDKQYSRSSSMPFLSRPPPSSCNMVSKVSELSSNDGTPIQGSSLSCKKRFKKSSSTSRLLNKVAAKATSIKDILSASMEGSLSATTISHASFFNGFTEQIRNEEDSSNTSLTNTLAEFDPFSPSSLYPEHEINATSDLNMDQDYDFSQFFEKFGGDNHNEENSMNDLLMSDVSQEVSSTSVDDQDNMVGNFEGWSNYLLDHTNFMYDTDSDSLEKHFI

>AtMYB76

MSKRPYCIGEGLKKGAWTTEEDKKLISYIHDHGEGGWRDIPEKAGLKRCGKSCRLRWTNYLKPDIKRGEFSYEEEQIIIMLHASRGNKWSVIARHLPKRTDNEVKNYWNTHLKKRLIDDGIDPVTHKPLASSNPNPVEPMKFDFQKKSNQDEHSSQSSSTTPASLPLSSNLNSVKSKISSGETQIESGHVSCKKRFGRSSSTSRLLNKVAARASSIGNILSTSIEGTLRSPASSSGLPDSFSQSYEYMIDNKEDLGTSIDLNIPEYDFPQFLEQLINDDDENENIVGPEQDLLMSDFPSTFVDEDDILGDITSWSTYLLDHPNFMYESDQDSDEKNFL

>AtMYB29

MSRKPCCVGEGLKKGAWTAEEDKKLISYIHEHGEGGWRDIPQKAGLKRCGKSCRLRWANYLKPDIKRGEFSYEEEQIIIMLHASRGNKWSVIARHLPKRTDNEIKNYWNTHLKKLLIDKGIDPVTHKPLAYDSNPDEQSQSGSISPKSLPPSSSKNVPEITSSDETPKYDASLSSKKRCFKRSSSTSKLLNKVAARASSMGTILGASIEGTLISSTPLSSCLNDDFSETSQFQMEEFDPFYQSSEHIIDHMKEDISINNSEYDFSQFLEQFSNNEGEEADNTGGGYNQDLLMSDVSSTSVDEDEMMQNITGWSNYLLDHSDFNYDTSQDYDDKNFI

>AtMYB4

MGRSPCCEKAHTNKGAWTKEEDERLVAYIKAHGEGCWRSLPKAAGLLRCGKSCRLRWINYLRPDLKRGNFTEEEDELIIKLHSLLGNKWSLIAGRLPGRTDNEIKNYWNTHIRRKLINRGIDPTSHRPIQESSASQDSKPTQLEPVTSNTINISFTSAPKVETFHESISFPGKSEKISMLTFKEEKDECPVQEKFPDLNLELRISLPDDVDRLQGHGKSTTPRCFKCSLGMINGMECRCGRMRCDVVGGSSKGSDMSNGFDFLGLAKKETTSLLGFRSLEMK

>AtMYB32

MGRSPCCEKDHTNKGAWTKEEDDKLISYIKAHGEGCWRSLPRSAGLQRCGKSCRLRWINYLRPDLKRGNFTLEEDDLIIKLHSLLGNKWSLIATRLPGRTDNEIKNYWNTHVKRKLLRKGIDPATHRPINETKTSQDSSDSSKTEDPLVKILSFGPQLEKIANFGDERIQKRVEYSVVEERCLDLNLELRISPPWQDKLHDERNLRFGRVKYRCSACRFGFGNGKECSCNNVKCQTEDSSSSSYSSTDISSSIGYDFLGLNNTRVLDFSTLEMK

>AtMYB7

MGRSPCCEKEHMNKGAWTKEEDERLVSYIKSHGEGCWRSLPRAAGLLRCGKSCRLRWINYLRPDLKRGNFTHDEDELIIKLHSLLGNKWSLIAARLPGRTDNEIKNYWNTHIKRKLLSKGIDPATHRGINEAKISDLKKTKDQIVKDVSFVTKFEETDKSGDQKQNKYIRNGLVCKEERVVVEEKIGPDLNLELRISPPWQNQREISTCTASRFYMENDMECSSETVKCQTENSSSISYSSIDISSSNVGYDFLGLKTRILDFRSLEMK

>AtMYB3

MIIFVKDSETLCCCYRWSLIAGRLPGRTDNEIKNYWNTHIKRKLLSRGIDPNSHRLINESVVSPSSLQNDVVETIHLDFSGPVKPEPVREEIGMVNNCESSGTTSEKDYGNEEDWVLNLELSVGPSYRYESTRKVSVVDSAESTRRWGSELFGAHESDAVCLCCRIGLFRNESCRNCRVSDVRTH

>AtMYB8

MGRSPCCEKAHTNKGAWTKEEDQRLVDYIRNHGEGCWRSLPKSAGLLRCGKSCRLRWINYLRPDLKRGNFTDDEDQIIIKLHSLLGNKWSLIAGRLPGRTDNEIKNYWNTHIKRKLLSHGIDPQTHRQINESKTVSSQVVVPIQNDAVEYSFSNLAVKPKTENSSDNGASTSGTTTDEDLRQNGECYYSDNSGHIKLNLDLTLGFGSWSGRIVGVGSSADSKPWCDPVMEARLSLL

>AtMYB6

MGRSPCCEKAHTNKGAWTKEEDQRLVDYIRNHGEGCWRSLPKSAGLLRCGKSCRLRWINYLRPDLKRGNFTDDEDQIIIKLHSLLGNKWSLIAGRLPGRTDNEIKNYWNTHIKRKLLSHGIDPQTHRQINESKTVSSQVVVPIQNDAVEYSFSNLAVKPKTENSSDNGASTSGTTTDEDLRQNGECYYSDNSGHIKLNLDLTLGFGSWSGRIVGVGSSADSKPWCDPVMEARLSLL

>AtMYB123

MKTRSLEITSPLTAKANGALSLTKLVLISLSTDPSLYLLRTIIVLAHIKGLKRCGKSCRLRWKNYLRPGIKRGNISSDEEELIIRLHNLLGNRWSLIAGRLPGRTDNEIKNHWNSNLRKRLPKTQTKQPKRIKHSTNNENNVCVIRTKAIRCSKTLLFSDLSLQKKSSTSPLPLKEQEMDQGGSSLMGDLEFDFDRIHSEFHFPDLMDFDGLDCGNVTSLVSSNEILGELVPAQGNLDLNRPFTSCHHRGDDEDWLRDFTC

>AtMYB111

MGRAPCCEKIGLKRGRWTAEEDEILTKYIQTNGEGSWRSLPKKAGLLRCGKSCRLRWINYLRRDLKRGNITSDEEEIIVKLHSLLGNRWSLIATHLPGRTDNEIKNYWNSHLSRKIYAFTAVSGDGHNLLVNDVVLKKSCSSSSGAKNNNKTKKKKKGRTSRSSMKKHKQMVTASQCFSQPKELESDFSEGGQNGNFEGESLGPYEWLDGELERLLSSCVWECTSEEAVIGVNDEKVCESGDNSSCCVNLFEEEQGSETKIGHVGITEVDHDMTVEREREGSFLSSNSNENNDKDWWVGLCNSSEVGFGVDEELLDWEFQGNVTCQSDDLWDLSDIGEITLE

>AtMYB12

MGRAPCCEKVGIKRGRWTAEEDQILSNYIQSNGEGSWRSLPKNAGLKRCGKSCRLRWINYLRSDLKRGNITPEEEELVVKLHSTLGNRWSLIAGHLPGRTDNEIKNYWNSHLSRKLHNFIRKPSISQDVSAVIMTNASSAPPPPQAKRRLGRTSRSAMKPKIHRTKTRKTKKTSAPPEPNADVAGADKEALMVESSGAEAELGRPCDYYGDDCNKNLMSINGDNGVLTFDDDIIDLLLDESDPGHLYTNTTCGGDGELHNIRDSEGARGFSDTWNQGNLDCLLQSCPSVESFLNYDHQVNDASTDEFIDWDCVWQEGSDNNLWHEKENPDSMVSWLLDGDDEATIGNSNCENFGEPLDHDDESALVAWLLS

>AtMYB11

MGRAPCCEKVGIKKGRWTAEEDRTLSDYIQSNGEGSWRSLPKNAGLKRCGKSCRLRWINYLRSDIKRGNITPEEEDVIVKLHSTLGTRWSTIASNLPGRTDNEIKNYWNSHLSRKLHGYFRKPTVANTVENAPPPPKRRPGRTSRSAMKPKFILNPKNHKTPNSFKANKSDIVLPTTTIENGEGDKEDALMVLSSSSLSGAEEPGLGPCGYGDDGDCNPSINGDDGALCLNDDIFDSCFLLDDSHAVHVSSCESNNVKNSEPYGGMSVGHKNIETMADDFVDWDFVWREGQTLWDEKEDLDSVLSRLLDGEEMESEIRQRDSNDFGEPLDIDEENKMAAWLLS

>AtMYB5

MMSCGGKKPVSKKTTPCCTKMGMKRGPWTVEEDEILVSFIKKEGEGRWRSLPKRAGLLRCGKSCRLRWMNYLRPSVKRGGITSDEEDLILRLHRLLGNRWSLIAGRIPGRTDNEIKNYWNTHLRKKLLRQGIDPQTHKPLDANNIHKPEEEVSGGQKYPLEPISSSHTDDTTVNGGDGDSKNSINVFGGEHGYEDFGFCYDDKFSSFLNSLINDVGDPFGNIIPISQPLQMDDCKDGIVGASSSSLGHD

>AtMYB113

MGESPKGLRKGTWTTEEDILLRQCIDKYGEGKWHRVPLRTGLNRCRKSCRLRWLNYLKPSIKRGKLCSDEVDLVLRLHKLLGNRWSLIAGRLPGRTANDVKNYWNTHLSKKHDERCCKTKMINKNITSHPTSSAQKIDVLKPRPRSFSDKNSCNDVNILPKVDVVPLHLGLNNNYVCESSITCNKDEQKDKLININLLDGDNMWWESLLEADVLGPEATETAKGVTLPLDFEQIWARFDEETLELN

>AtMYB114

MEGSSKGLRKGAWTAEEDSLLRQCIGKYGEGKWHQVPLRAGLNRCRKSCRLRWLNYLKPSIKRGKFSSDEVDLLLRLHKLLGNRWSLIAGRLPGRTANDVKNYWNTHLSKKHEPCCKTKIKRINIITPPNTPAQKVDIF

>AtMYB90

MEGSSKGLRKGAWTAEEDSLLRLCIDKYGEGKWHQVPLRAGLNRCRKSCRLRWLNYLKPSIKRGRLSNDEVDLLLRLHKLLGNRWSLIAGRLPGRTANDVKNYWNTHLSKKHESSCCKSKMKKKNIISPPTTPVQKIGVFKPRPRSFSVNNGCSHLNGLPEVDLIPSCLGLKKNNVCENSITCNKDDEKDDFVNNLMNGDNMWLENLLGENQEADAIVPEATTAEHGATLAFDVEQLWSLFDGETVELD

>AtMYB75

MEGSSKGLRKGAWTTEEDSLLRQCINKYGEGKWHQVPVRAGLNRCRKSCRLRWLNYLKPSIKRGKLSSDEVDLLLRLHRLLGNRWSLIAGRLPGRTANDVKNYWNTHLSKKHEPCCKIKMKKRDITPIPTTPALKNNVYKPRPRSFTVNNDCNHLNAPPKVDVNPPCLGLNINNVCDNSIIYNKDKKKDQLVNNLIDGDNMWLEKFLEESQEVDILVPEATTTEKGDTLAFDVDQLWSLFDGETVKFD

>AtMYB82

MECKREEGKSYVKRGLWKPEEDMILKSYVETHGEGNWADISRRSGLKRGGKSCRLRWKNYLRPNIKRGSMSPQEQDLIIRMHKLLGNRWSLIAGRLPGRTDNEVKNYWNTHLNKKPNSRRQNAPESIVGATPFTDKPVMSTELRRSHGEGGEEESNTWMEETNHFGYDVHVGSPLPLISHYPDNTLVFDPCFSFTDFFPLL

>AtMYB0

MRIRRRDEKENQEYKKGLWTVEEDNILMDYVLNHGTGQWNRIVRKTGLKRCGKSCRLRWMNYLSPNVNKGNFTEQEEDLIIRLHKLLGNRWSLIAKRVPGRTDNQVKNYWNTHLSKKLVGDYSSAVKTTGEDDDSPPSLFITAATPSSCHHQQENIYENIAKSFNGVVSASYEDKPKQELAQKDVLMATTNDPSHYYGNNALWVHDDDFELSSLVMMNFASGDVEYCL

>AtMYB66

MRKKVSSSGDEGNNEYKKGLWTVEEDKILMDYVKAHGKGHWNRIAKKTGLKRCGKSCRLRWMNYLSPNVKRGNFTEQEEDLIIRLHKLLGNRWSLIAKRVPGRTDNQVKNYWNTHLSKKLGIKDQKTKQSNGDIVYQINLPNPTETSEETKISNIVDNNNILGDEIQEDHQGSNYLSSLWVHEDEFELSTLTNMMDFIDGHCF

>AtMYB23

MRMTRDGKEHEYKKGLWTVEEDKILMDYVRTHGQGHWNRIAKKTGLKRCGKSCRLRWMNYLSPNVNRGNFTDQEEDLIIRLHKLLGNRWSLIAKRVPGRTDNQVKNYWNTHLSKKLGLGDHSTAVKAACGVESPPSMALITTTSSSHQEISGGKNSTLRFDTLVDESKLKPKSKLVHATPTDVEVAATVPNLFDTFWVLEDDFELSSLTMMDFTNGYCL

>AtMYB49

MGKSSSSEESEVKKGPWTPEEDEKLVGYIQTHGPGKWRTLPKNAGLKRCGKSCRLRWTNYLRPDIKRGEFSLQEEETIIQLHRLLGNKWSAIAIHLPGRTDNEIKNYWNTHIKKKLLRMGIDPVTHCPRINLLQLSSFLTSSLFKSMSQPMNTPFDLTTSNINPDILNHLTASLNNVQTESYQPNQQLQNDLNTDQTTFTGLLNSTPPVQWQNNGEYLGDYHSYTGTGDPSNNKVPQAGNYSSAAFVSDHINDGENFKAGWNFSSSMLAGTSSSSSTPLNSSSTFYVNGGSEDDRESFGSDMLMFHHHHDHNNNALNLS

>AtMYB41

MGRSPCCDKNGVKKGPWTAEEDQKLIDYIRFHGPGNWRTLPKNAGLHRCGKSCRLRWTNYLRPDIKRGRFSFEEEETIIQLHSVMGNKWSAIAARLPGRTDNEIKNHWNTHIRKRLVRSGIDPVTHSPRLDLLDLSSLLSALFNQPNFSAVATHASSLLNPDVLRLASLLLPLQNPNPVYPSNLDQNLQTPNTSSESSQPQAETSTVPTNYETSSLEPMNARLDDVGLADVLPPLSESFDLDSLMSTPMSSPRQNSIEAETNSSTFFDFGIPEDFILDDFMF

>AtMYB102

MARSPCCEKNGLKKGPWTSEEDQKLVDYIQKHGYGNWRTLPKNAGLQRCGKSCRLRWTNYLRPDIKRGRFSFEEEETIIQLHSFLGNKWSAIAARLPGRTDNEIKNFWNTHIRKKLLRMGIDPVTHSPRLDLLDISSILASSLYNSSSHHMNMSRLMMDTNRRHHQQHPLVNPEILKLATSLFSQNQNQNLVVDHDSRTQEKQTVYSQTGVNQYQTNQYFENTITQELQSSMPPFPNEARQFNNMDHHFNGFGEQNLVSTSTTSVQDCYNPSFNDYSSSNFVLDPSYSDQSFNFANSVLNTPSSSPSPTTLNSSYINSSSCSTEDEIESYCSNLMKFDIPDFLDVNGFII

>AtMYB74

MGRSPCCEKKNGLKKGPWTPEEDQKLIDYINIHGYGNWRTLPKNAGLQRCGKSCRLRWTNYLRPDIKRGRFSFEEEETIIQLHSIMGNKWSAIAARLPGRTDNEIKNYWNTHIRKRLLKMGIDPVTHTPRLDLLDISSILSSSIYNSSHHHHHHHQQHMNMSRLMMSDGNHQPLVNPEILKLATSLFSNQNHPNNTHENNTVNQTEVNQYQTGYNMPGNEELQSWFPIMDQFTNFQDLMPMKTTVQNSLSYDDDCSKSNFVLEPYYSDFASVLTTPSSSPTPLNSSSSTYINSSTCSTEDEKESYYSDNITNYSFDVNGFLQFQ

>AtMYB95

MGRTTWFDVDGLRKGEWTAEEDRKLVVYINEHGLGEWGSLPKRAGLQRCGKSCRLRWLNYLRPGIKRGKFTPQEEEEIIKYHALLGNRWAAIAKQMPNRTDNDIKNHWNSCLKKRLAKKGIDPMTHEPTTTTSLTVDVTSSSTTSSPTPSPTSSSFSSCSSTGSARFLNKLAAGISSRKHGLESIKTVILAEQPREAVDEEKMMTINMKEKELISCYMEIDETMSIDELPCDDSTSGFVAFDDYSLIDPYRDGVYVSDFYDETEHLDLFLL

>AtMYB47

MGRTTWFDVDGLRKGEWTAEEDRKLVVYINEHGLGEWGSLPKRAGLQRCGKSCRLRWLNYLRPGIKRGKFTPQEEEEIIKYHALLGNRWAAIAKQMPNRTDNDIKNHWNSCLKKRLAKKGIDPMTHEPTTTTSLTVDVTSSSTTSSPTPSPTSSSFSSCSSTGSARFLNKLAAGISSRKHGLESIKTVILAEQPREAVDEEKMMTINMKEKELISCYMEIDETMSIDELPCDDSTSGFVAFDDYSLIDPYRDGVYVSDFYDETEHLDLFLL

>AtMYB17

MGRTPCCDKIGLKKGPWTPEEDEVLVAHIKKNGHGSWRTLPKLAGLLRCGKSCRLRWTNYLRPDIKRGPFTADEEKLVIQLHAILGNRWAAIAAQLPGRTDNEIKNLWNTHLKKRLLSMGLDPRTHEPLPSYGLAKQAPSSPTTRHMAQWESARVEAEARLSRESMLFSPSFYSGVVKTECDHFLRIWNSEIGEAFRNLAPLDESTITSQSPCSRATSTSSALLKSSTNSWGGKEVTVAIHGSDYSPYSNDLEDDSTDSALQLLLDFPISDDDMSFLEENIDSYSQAPPIGLVSMVSKF

>AtMYB106

MGRSPCCDKAGLKKGPWTPEEDQKLLAYIEEHGHGSWRSLPEKAGLQRCGKSCRLRWTNYLRPDIKRGKFTVQEEQTIIQLHALLGNRWSAIATHLPKRTDNEIKNYWNTHLKKRLIKMGIDPVTHKHKNETLSSSTGQSKNAATLSHMAQWESARLEAEARLARESKLLHLQHYQNNNNLNKSAAPQQHCFTQKTSTNWTKPNQGNGDQQLESPTSTVTFSENLLMPLGIPTDSSRNRNNNNNESSAMIELAVSSSTSSDVSLVKEHEHDWIRQINCGSGGIGEGFTSLLIGDSVGRGLPTGKNEATAGVGNESEYNYYEDNKNYWNSILNLVDSSPSDSATMF

>AtMYB16

MILFLFVYRWSAIATHLPKRTDNEIKNYWNTHLKKRLVKMGIDPVTHKPKNETPLSSLGLSKNAAILSHTAQWESARLEAEARLARESKLLHLQHYQTKTSSQPHHHHGFTHKSLLPNWTTKPHEDQQQLESPTSTVSFSEMKESIPAKIEFVGSSTGVTLMKEPEHDWINSTMHEFETTQMGEGIEEGFTGLLLGGDSIDRSFSGDKNETAGESSGGDCNYYEDNKNYLDSIFNFVDPSPSDSPMF

>AtMYB93

MGRSPCCDENGLKKGPWTPEEDQKLIDYIHKHGHGSWRALPKLADLNRCGKSCRLRWTNYLRPDIKRGKFSAEEEQTILHLHSILGNKWSAIATHLQGRTDNEIKNFWNTHLKKKLIQMGIDPVTHQPRTDLFASLPQLIALANLKDLIEQTSQFSSMQGEAAQLANLQYLQRMFNSSASLTNNNGNNFSPSSILDIDQHHAMNLLNSMVSWNKDQNPAFDPVLELEANDQNQDLFPLGFIIDQPTQPLQQQKYHLNNSPSELPSQGDPLLDHVPFSLQTPLNSEDHFIDNLVKHPTDHEHEHDDNPSSWVLPSLIDNNPKTVTSSLPHNNPADASSSSSYGGCEAASFYWPDICFDESLMNVIS

>AtMYB92

MGRSPISDDSGLKKGPWTPDEDEKLVNYVQKHGHSSWRALPKLAGLNRCGKSCRLRWTNYLRPDIKRGRFSPDEEQTILNLHSVLGNKWSTIANQLPGRTDNEIKNFWNTHLKKKLIQMGFDPMTHRPRTDIFSGLSQLMSLSSNLRGFVDLQQQFPIDQEHTILKLQTEMAKLQLFQYLLQPSSMSNNVNPNDFDTLSLLNSIASFKETSNNTTSNNLDLGFLGSYLQDFHSLPSLKTLNSNMEPSSVFPQNLDDNHFKFSTQRENLPVSPIWLSDPSSTTPAHVNDDLIFNQYGIEDVNSNITSSSGQESGASASAAWPDHLLDDSIFSDIP

>AtMYB53

MGRSPSSDETGLKKGPWLPEEDDKLINYIHKHGHSSWSALPKLAGLNRCGKSCRLRWTNYLRPDIKRGKFSAEEEETILNLHAVLGNKWSMIASHLPGRTDNEIKNFWNTHLKKKLIQMGFDPMTHQPRTDDIFSSLSQLMSLSNLRGLVDLQQQFPMEDQALLNLQTEMAKLQLFQYLLQPSPAPMSINNINPNILNLLIKENSVTSNIDLGFLSSHLQDFNNNNLPSLKTLDDNHFSQNTSPIWLHEPPSLNQTMLPTHDPCAQSVDGFGSNQASSSHDQEVAVTDSVDWPDHHLFDDSMFPDISYQS

>AtMYB39

MEEEEKNPSSIYIVADLLEDIFLRLPLKSILISKSVSKRWRSILESKTFVERRMSLQKKRKILAAYNCKCGWEPRLLPGSSQCKGNEEIVYLHCNAAQPSFTCDGLVCILEPRWIDVLNPWTRQLRRYGFGFGTIFGVGSAFSPRHWAMGFGKDKVTGSYKVVKMCLISFSEICARDPEVEYSVLDVETGEWRMLSPPPYKVFEVRKSECANGSIYWLHKPTERAWTILALDLHKEELHNISVPDMSVTQETFQIVNLEDRLAIANTYTKTEWKLEIWSMDTEVETWTKTYSIDLENRVASRERRNRWFTPVSVSKQGNIVFYDNHKRLFKYYPRKNEILYLSADTCVISPFFENLAPLPQKSTLHTPIIAGEPNAPLTTLVVVGSIWSPLFLFSVKL

>AtMYB107

MGRSPCCDESGLKKGPWTPEEDQKLINHIRKHGHGSWRALPKQAGLNRCGKSCRLRWTNYLRPDIKRGNFTAEEEQTIINLHSLLGNKWSSIAGHLPGRTDNEIKNYWNTHIRKKLIQMGIDPVTHRPRTDHLNVLAALPQLLAAANFNNLLNLNQNIQLDATSVAKAQLLHSMIQVLSNNNTSSSFDIHHTTNNLFGQSSFLENLPNIENPYDQTQGLSHIDDQPLDSFSSPIRVVAYQHDQNFIPPLISTSPDESKETQMMVKNKEIMKYNDHTSNPSSTSTFTQDHQPWCDIIDDEASDSYWKEIIE

>AtMYB9

MGRSPCCDENGLKKGPWTQEEDDKLIDHIQKHGHGSWRALPKQAGLNRCGKSCRLRWTNYLRPDIKRGNFTEEEEQTIINLHSLLGNKWSSIAGNLPGRTDNEIKNYWNTHLRKKLLQMGIDPVTHRPRTDHLNVLAALPQLIAAANFNSLLNLNQNVQLDATTLAKAQLLHTMIQVLSTNNNTTNPSFSSSTMQNSNTNLFGQASYLENQNLFGQSQNFSHILEDENLMVKTQIIDNPLDSFSSPIQPGFQDDHNSLPLLVPASPEESKETQRMIKNKDIVDYHHHDASNPSSSNSTFTQDHHHPWCDTIDDGASDSFWKEIIEQTCSEPWPFPE

>AtMYB38

MSLSLIITEPENSFSFPFFSFKKQNKDISTKTLRPRETDREMGRAPCCDKANVKRGPWSPEEDAKLKDYIEKQGTGGNWIALPHKAGLRRCGKSCRLRWLNYLRPNIRHGDFTEEEDNIIYSLFASIGSRWSVIAAHLQGRTDNDIKNYWNTKLKKKLIATMAPPPHHHLAIATSSSSASPSSSSHYNMINSLLPYNPSTNQLLTPHQGIMMTMMGQQQQLFYQEDMGNLVNSPNRNNLIMSHQEDNQEQSTNKGIMLLSDVRSGSSTTSTVTRVKMEHRDHDDHHHHHEEDERSMTSVVMEDYGMEEIKQLISSSCTSSNNSLWFDENKTEDKFMLYY

>AtMYB37

MGRAPCCDKTKVKRGPWSPEEDSKLRDYIEKYGNGGNWISFPLKAGLRRCGKSCRLRWLNYLRPNIKHGDFSEEEDRIIFSLFAAIGSRWSIIAAHLPGRTDNDIKNYWNTKLRKKLLSSSSDSSSSAMASPYLNPISQDVKRPTSPTTIPSSSYNPYAENPNQYPTKSLISSINGFEAGDKQIISYINPNYPQDLYLSDSNNNTSNANGFLLNHNMCDQYKNHTSFSSDVNGIRSEIMMKQEEIMMMMMIDHHIDQRTKGYNGEFTQGYYNYYNGHGDLKQMISGTGTNSNINMGGSGSSSSSISNLAENKSSGSLLLEYKCLPYFYS

>AtMYB84

MGRAPCCDKANVKKGPWSPEEDAKLKSYIENSGTGGNWIALPQKIGLKRCGKSCRLRWLNYLRPNIKHGGFSEEEENIICSLYLTIGSRWSIIAAQLPGRTDNDIKNYWNTRLKKKLINKQRKELQEACMEQQEMMVMMKRQHQQQQIQTSFMMRQDQTMFTWPLHHHNVQVPALFMNQTNSFCDQEDVKPVLIKNMVKIEDQELEKTNPHHHQDSMTNAFDHLSFSQLLLDPNHNHLGSGEGFSMNSILSANTNSPLLNTSNDNQWFGNFQAETVNLFSGASTSTSADQSTISWEDISSLVYSDSKQFF

>AtMYB68

MGRAPCCDKANVKKGPWSPEEDAKLKDYIENSGTGGNWIALPQKIGLRRCGKSCRLRWLNYLRPNIKHGGFSEEEDNIICNLYVTIGSRWSIIAAQLPGRTDNDIKNYWNTRLKKKLLNKQRKEFQEARMKQEMVMMKRQQQGQGQGQSNGSTDLYLNNMFGSSPWPLLPQLPPPHHQIPLGMMEPTSCNYYQTTPSCNLEQKPLITLKNMVKIEEEQERTNPDHHHQDSVTNPFDFSFSQLLLDPNYYLGSGGGGEGDFAIMSSSTNSPLPNTSSDQHPSQQQEILQWFGSSNFQTEAINDMFINNNNNIVNLETIENTKVYGDASVAGAAVRAALGGGTTSTSADQSTISWEDITSLVNSEDASYFNAPNHV

>AtMYB87

MGRAPCCDKMAVKKGPWSTEEDAVLKSYIEKHGTGNNWISLPQRIGIKRCGKSCRLRWLNYLRPNLKHGGFTDEEDYIICSLYITIGSRWSIIASQLPGRTDNDIKNYWNTRLKKKLLSKQGKAFHQQLNVKFERGTTSSSSSQNQIQIFHDENTKSNQTLYNQVVDPSMRAFAMEEQSMIKNQILEPFSWEPNKVLFDVDYDAAASSYHHHASPSLNSMSSTSSIGTNNSSLQMSHYTVNHNDHDQPDMFFMDGFENFQAELFDEIANNNTVENGFDGTEILINNNYLDHDISSFIDYPLYDNE

>AtMYB36

MGRAPCCDKANVKKGPWSPEEDVKLKDYIDKYGTGGNWIALPQKIGLKRCGKSCRLRWLNYLRPNIKHGGFSEEEDRIILSLYISIGSRWSIIAAQLPGRTDNDIKNYWNTKLKKKLLGRQKQMNRQDSITDSTENNLSNNNNNKSPQNLSNSALERLQLHMQLQNLQSPFSSFYNNPILWPKLHPLLQSTTTNQNPKLASQESFHPLGVNVDHQHNNTKLAQINNGASSLYSENVEQSQNPAHEFQPNFGFSQDLRLDNHNMDFMNRGVSKELFQVGNEFELTNGSSWWSEEVELERKTTSSSSWGSASVLDQTTEGMVMLQDYAQMSYHSV

>AtMYB80

MGRIPCCEKENVKRGQWTPEEDNKLASYIAQHGTRNWRLIPKNAGLQRCGKSCRLRWTNYLRPDLKHGQFSEAEEHIIVKFHSVLGNRWSLIAAQLPGRTDNDVKNYWNTKLKKKLSGMGIDPVTHKPFSHLMAEITTTLNPPQVSHLAEAALGCFKDEMLHLLTKKRVDLNQINFSNHNPNPNNFHEIADNEAGKIKMDGLDHGNGIMKLWDMGNGFSYGSSSSSFGNEERNDGSASPAVAAWRGHGGIRTAVAETAAAEEEERRKLKGEVVDQEEIGSEGGRGDGMTMMRNHHHHQHVFNVDNVLWDLQADDLINHMV

>AtMYB35

MGRPPCCDKSNVKKGLWTEEEDAKILAYVAIHGVGNWSLIPKKAGLNRCGKSCRLRWTNYLRPDLKHDSFSTQEEELIIECHRAIGSRWSSIARKLPGRTDNDVKNHWNTKLKKKLMKMGIDPVTHKPVSQLLAEFRNISGHGNASFKTEPSNNSILTQSNSAWEMMRNTTTNHESYYTNSPMMFTNSSEYQTTPFHFYSHPNHLLNGTTSSCSSSSSSTSITQPNQVPQTPVTNFYWSDFLLSDPVPQVVGSSATSDLTFTQNEHHFNIEAEYISQNIDSKASGTCHSASSFVDEILDKDQEMLSQFPQLLNDFDY

>AtMYB60

MGRPPCCDKIGIKKGPWTPEEDIILVSYIQEHGPGNWRSVPTNTGLLRCSKSCRLRWTNYLRPGIKRGNFTPHEEGMIIHLQALLGNKWASIASYLPQRTDNDIKNYWNTHLKKKLNKSDSDERSRSENIALQTSSTRNTINHRSTYASSTENISRLLEGWMRASPKSSTSTTFLEHKMQNRTNNFIDHHSDQFPYEQLQGSWEEGHSKGINGDDDQGIKNSENNNGDDVHHEDGDHEDDDDHNATPPLTFIEKWLLEETSTTGGQMEEMSHLMELSNML

>AtMYB31

MGRPPCCEKIEVKKGPWTPEEDIILVSYIQQHGPGNWRSVPANTGLLRCSKSCRLRWTNYLRPGIKRGNFTQPEEKMIIHLQALLGNRWAAIASYLPQRTDNDIKNYWNTHLKKKLVMMKFQNGIINENKTNLATDISSCNNNNNGCNHNKRTTNKGQWEKKLQTDINMAKQALFQALSLDQPSSLIPPDPDSPKPHHHSTTTYASSTDNISKLLQNWTSSSSSKPNTSSVSNNRSSSPGEGGLFDHHSLFSSNSESGSVDEKLNLMSETSMFKGESKPDIDMEATPTTTTTDDQGSLSLIEKWLFDDQGLVQCDDSQEDLIDVSLEELK

>AtMYB30

MVRPPCCDKGGVKKGPWTPEEDIILVTYIQEHGPGNWRAVPTNTGLLRCSKSCRLRWTNYLRPGIKRGNFTEHEEKMIVHLQALLGNRWAAIASYLPQRTDNDIKNYWNTHLKKKLNKVNQDSHQELDRSSLSSSPSSSSANSNSNISRGQWERRLQTDIHLAKKALSEALSPAVAPIITSTVTTTSSSAESRRSTSSASGFLRTQETSTTYASSTENIAKLLKGWVKNSPKTQNSADQIASTEVKEVIKSDDGKECAGAFQSFSEFDHSYQQAGVSPDHETKPDITGCCSNQSQWSLFEKWLFEDSGGQIGDILLDENTNFF

>AtMYB96

MGRPPCCEKIGVKKGPWTPEEDIILVSYIQEHGPGNWRSVPTHTGLRRCSKSCRLRWTNYLRPGIKRGNFTEHEEKTIVHLQALLGNRWAAIASYLPERTDNDIKNYWNTHLKKKLKKINESGEEDNDGVSSSNTSSQKNHQSTNKGQWERRLQTDINMAKQALCEALSLDKPSSTLSSSSSLPTPVITQQNIRNFSSALLDRCYDPSSSSSSTTTTTTSNTTNPYPSGVYASSAENIARLLQDFMKDTPKALTLSSSSPVSETGPLTAAVSEEGGEGFEQSFFSFNSMDETQNLTQETSFFHDQVIKPEITMDQDHGLISQGSLSLFEKWLFDEQSHEMVGMALAGQEGMF

>AtMYB94

MGRPPCCDKIGVKKGPWTPEEDIILVSYIQEHGPGNWRSVPTHTGLRRCSKSCRLRWTNYLRPGIKRGNFTEHEEKMILHLQALLGNRWAAIASYLPERTDNDIKNYWNTHLKKKLKKMNDSCDSTINNGLDNKDFSISNKNTTSHQSSNSSKGQWERRLQTDINMAKQALCDALSIDKPQNPTNFSIPDLGYGPSSSSSSTTTTTTTTRNTNPYPSGVYASSAENIARLLQNFMKDTPKTSVPLPVAATEMAITTAASSPSTTEGDGEGIDHSLFSFNSIDEAEEKPKLIDHDINGLITQGSLSLFEKWLFDEQSHDMIINNMSLEGQEVLF

>AtMYB85

MGRQPCCDKLGVKKGPWTVEEDKKLINFILTNGHCCWRALPKLAGLRRCGKSCRLRWTNYLRPDLKRGLLSHDEEQLVIDLHANLGNKWSKIASRLPGRTDNEIKNHWNTHIKKKLLKMGIDPMTHQPLNQEPSNIDNSKTIPSNPDDVSVEPKTTNTKYVEISVTTTEEESSSTVTDQNSSMDNENHLIDNIYDDDELFSYLWSDETTKDEASWSDSNFGVGGTLYDHNISGADADFPIWSPERINDEKMFLDYCQDFGVHDFGF

>AtMYB42

MGRQPCCDKLMVKKGPWTAEEDKKLINFILTNGHCCWRALPKLAGLRRCGKSCRLRWTNYLRPDLKRGLLSDAEEQLVIDLHALLGNRWSKIAARLPGRTDNEIKNHWNTHIKKKLLKMEIDPSTHQPLNKVFTDTNLVDKSETSSKADNVNDNKIVEIDGTTTNTIDDSIITHQNSSNDDYELLGDIIHNYGDLFNILWTNDEPPLVDDASWSNHNVGIGGTAAVAASDKNNTAAEEDFPERSFEKQNGESWMFLDYCQEFGVEDFGFECYHGFGQSSMKTGHKD

>AtMYB99

MGGRKPCCDEVGLRKGPWTVEEDGKLVDFLRARGNCGGGGGGWCWRDVPKLAGLRRCGKSCRLRWTNYLRPDLKRGLFTEEEIQLVIDLHARLGNRWSKIAVELPGRTDNDIKNYWNTHIKRKLIRMGIDPNTHRRFDQQKVNEEETILVNDPKPLSETEVSVALKNDTSAVLSGNLNQLADVDGDDQPWSFLMENDEGGGGDAAGELTMLLSGDITSSCSSSSSLWMKYGEFGYEDLELGCFDV

>AtMYB40

MFYGCFGHCRWSKIASHFSGRTDNEIKNHWNTKIKKKMKHLGLDPATHKPMNDITHQTDPNQDKKPNMCSTINEGEEIKDQTPKDDVITETTKTLMLSDNDEELVAKNCKILCAEEVDLESLFETQCNEISSSSFSSLCSNISRSESSSYLAEDSISLEQWDLDMTDPFVPWDLFANLDDNLFLLU

>AtMYB43

MGRQPCCDKVGLKKGPWTIEEDKKLINFILTNGHCCWRALPKLSGLLRCGKSCRLRWINYLRPDLKRGLLSEYEEQKVINLHAQLGNRWSKIASHLPGRTDNEIKNHWNTHIKKKLRKMGIDPLTHKPLSEQEASQQAQGRKKSLVPHDDKNPKQDQQTKDEQEQHQLEQALEKNNTSVSGDGFCIDEVPLLNPHEILIDISSSHHHHSNDDNVNINTSKFTSPSSSSSSTSSCISSVVPGDEFSKFFDEMEILDLKWLSSDDSLGDDISKDGKFNNSTVDTMNLWDINDLSSLDMFMNEHDDGFIGNGNGCSRMVLDQDSWTFDLL

>AtMYB20

MGRQPCCDKVGLKKGPWTAEEDRKLINFILTNGQCCWRAVPKLSGLLRCGKSCRLRWTNYLRPDLKRGLLSDYEEKMVIDLHSQLGNRWSKIASHLPGRTDNEIKNHWNTHIKKKLRKMGIDPLTHKPLSIVEKEDEEPLKKLQNNTVPFQETMERPLENNIKNISRLEESLGDDQFMEINLEYGVEDVPLIETESLDLICSNSTMSSSTSTSSHSSNDSSFLKDLQFPEFEWSDYGNSNNDNNNGVDNIIENNMMSLWEISDFSSLDLLLNDESSSTFGLF

>AtMYB15

MGRAPCCEKMGLKRGPWTPEEDQILVSFILNHGHSNWRALPKQAGLLRCGKSCRLRWMNYLKPDIKRGNFTKEEEDAIISLHQILGNRWSAIAAKLPGRTDNEIKNVWHTHLKKRLEDYQPAKPKTSNKKKGTKPKSESVITSSNSTRSESELADSSNPSGESLFSTSPSTSEVSSMTLISHDGYSNEINMDNKPGDISTIDQECVSFETFGADIDESFWKETLYSQDEHNYVSNDLEVAGLVEIQQEFQNLGSANNEMIFDSEMDFWFDVLARTGGEQDLLAGL

>AtMYB14

MGRAPCCEKMGVKRGPWTPEEDQILINYIHLYGHSNWRALPKHAGLLRCGKSCRLRWINYLRPDIKRGNFTPQEEQTIINLHESLGNRWSAIAAKLPGRTDNEIKNVWHTHLKKRLSKNLNNGGDTKDVNGINETTNEDKGSVIVDTASLQQFSNSITTFDISNDNKDDIMSYEDISALIDDSFWSDVISVDNSNKNEKKIEDWEGLIDRNSKKCSYSNSKLYNDDMEFWFDVFTSNRRIEEFSDIPEF

>AtMYB13

MGRRPCCEKIGLKKGPWSAEEDRILINYISLHGHPNWRALPKLAGLLRCGKSCRLRWINYLRPDIKRGNFTPHEEDTIISLHQLLGNRWSAIAAKLPGRTDNEIKNVWHTHLKKRLHHSQDQNNKEDFVSTTAAEMPTSPQQQSSSSADISAITTLGNNNDISNSNKDSATSSEDVLAIIDESFWSEVVLMDCDISGNEKNEKKIENWEGSLDRNDKGYNHDMEFWFDHLTSSSCIIGEMSDISEF

>AtMYB72

MGKGRAPCCDKNKVKRGPWSPQEDLTLITFIQKHGHQNWRSLPKLAGLLRCGKSCRLRWINYLRPDVKRGNFSKKEEDAIIHYHQTLGNKWSKIASFLPGRTDNEIKNVWNTHLKKRLTPSSSSSSLSSTHDQSTKADHDKNCDGAQEEIHSGLNESQNSATSSHHQGECMHTKPELHEVNGLNEIQFLLDHDDFDDITSEFLQDNDILFPLDSLLHNHQTHISTQEMTREVTKSQSFDHPQPDIPCGFEDTNEESDLRRQLVESTTPNNEYDEWFNFIDNQTYFDDFNFVGEVCL

>AtMYB10

MGNRRAPCCDKSQVKRGPWSDEESERLRSFILKNGHQNWRSLPKLAGLMRCGKSCRLRWINYLRPGLKRGNFTKEEEDTIIHLHQAYGNKWSKIASNFPGRTDNEIKNVWNTHLKKRLVKRSISSSSSDVTNHSVSSTSSSSSSISSVLQDVIIKSERPNQEEEFGEILVEQMACGFEVDAPQSLECLFDDSQVPPPISKPDSLQTHGKSSDHEFWSRLIEPGFDDYNEWLIFLDNQTC

>AtMYB63

MGKGRAPCCDKTKVKRGPWSPEEDIKLISFIQKFGHENWRSLPKQSGLLRCGKSCRLRWINYLRPDLKRGNFTSEEEETIIKLHHNYGNKWSKIASQLPGRTDNEIKNVWHTHLKKRLAQSSGTADEPASPCSSDSVSRGKDDKSSHVEDSLNRETNHRNELSTSMSSGGSNQQDDPKIDELRFEYIEEAYSEFNDIIIQEVDKPDLLEIPFDSDPDIWSFLDTSNSFQQSTANENSSGSRATTEEESDEDEVKKWFKHLESELGLEEDDNQQQYKEEESSSSSLLKNYELMIH

>AtMYB58

MGKGRAPCCDKTKVKRGPWSHDEDLKLISFIHKNGHENWRSLPKQAGLLRCGKSCRLRWINYLRPDVKRGNFSAEEEDTIIKLHQSFGNKWSKIASKLPGRTDNEIKNVWHTHLKKRLSSETNLNADEAGSKGSLNEEENSQESSPNASMSFAGSNISSKDDDAQISQMFEHILTYSEFTGMLQEVDKPELLEMPFDLDPDIWSFIDGSDSFQQPENRALQESEEDEVDKWFKHLESELGLEENDNQQQQQQHKQGTEDEHSSSLLESYELLIH

>AtMYB101

MDGGGETTATATMEGRGLKKGPWTTTEDAILTEYVRKHGEGNWNAVQKNSGLLRCGKSCRLRWANHLRPNLKKGSFTPDEEKIIIDLHAKLGNKWARMASQLPGRTDNEIKNYWNTRMKRRQRAGLPLYPHEIQHQGIDIDDEFEFDLTSFQFQNQDLDHNHQNMIQYTNSSNTSSSSSSFSSSSSQPSKRLRPDPLVSTNPGLNPIPDSSMDFQMFSLYNNSLENDNNQFGFSVPLSSSSSSNEVCNPNHILEYISENSDTRNTNKKDIDAMSYSSLLMGDLEIRSSSFPLGLDNSVLELPSNQRPTHSFSSSPIIDNGVHLEPPSGNSGLLDALLEESQALSRGGLFKDVRVSSSDLCEVQDKRVKMDFENLLIDHLNSSNHSSLGANPNIHNKYNEP

TMVKVTVDDDDELLTSLLNNFPSTTTPLPDWYRVTEMQNEASYLAPPSGILMGNHQGNGRVEPPTVPPSSSVDPMASLGSCYWSNMPSIC

>AtMYB120

MIMYGGGGAGKDGGSTNHLSDGGVILKKGPWTAAEDEILAAYVRENGEGNWNAVQKNTGLARCGKSCRLRWANHLRPNLKKGSFTGDEERLIIQLHAQLGNKWARMAAQLPGRTDNEIKNYWNTRLKRLLRQGLPLYPPDIIPNHQLHPHPHHQQQQQHNHHHHHHQQQQQHQQMYFQPQSSQRNTPSSSPLPSPTPANAKSSSSFTFHTTTANLLHPLSPHTPNTPSQLSSTPPPPPLSSPLCSPRNNQYPTLPLFALPRSQINNNNNGNFTFPRPPPLLQPPSSLFAKRYNNANTPLNCINRVSTAPFSPVSRDSYTSFLTLPYPSPTAQTATYHNTNNPYSSSPSFSLNPSSSSYPTSTSSPSFLHSHYTPSSTSFHTNPVYSMKQEQLPSNQIPQIDGFNNVNNFTDNERQNHNLNSSGAHRRSSSCSLLEDVFEEAEALASGGRGRPPKRRQLTASLPNHNNNTNNNDNFFSVSFGHYDSSDNLCSLQGKTKTTYNTSNLNYSSLQVKCKMFMIKTQI

>AtMYB97

MIVYGGGASEDGEGGGVVLKKGPWTVAEDETLAAYVREYGEGNWNSVQKKTWLARCGKSCRLRWANHLRPNLRKGSFTPEEERLIIQLHSQLGNKWARMAAQLPGRTDNEIKNYWNTRLKRFQRQGLPLYPPEYSQNNHQQQMYPQQPSSPLPSQTPASSFTFPLLQPPSLCPKRCYNTAFSPKASYISSPTNFLVSSPTFLHTHSSLSSYQSTNPVYSMKHELSSNQIPYSASLGVYQVSKFSDNGDCNQNLNTGLHTNTCQLLEDLMEEAEALADSFRAPKRRQIMAALEDNNNNNNFFSGGFGHRVSSNSLCSLQGLTPKEDESLQMNTMQDEDITKLLDWGSESEEISNGQSSVITTENNLVLDDHQFAFLFPVDDDTNNLPGIC

>AtMYB104

MIQDQANDLLAMKKTFTKSKWKPEEDRILKDYVIQYGDRTWTHVPKRTGLPHNPASCRFRWMNHLKPSLKKGPFTDEEEKRVLQLHAVLGNKWSQMAREFPGRTDNEIKNFWNARRMRLKGKGLPVYPDEVREQAIRTAAQYGVKVELLNAHYSQDSLMAGNVEKPQELNNLALNQCSPYYQSTLANVQPSRNRVMEPETTFPFTGGSAMNEQNPTLLCNPYVESTQEQLPDSHLFGNVTYSSPPMPLIHEVENLELPSFQGFDFHEEPSSFGAEQYNPMLNLEPHNTLVQSPLIGQTPTDFPSSFYDELLDELLESVVNGSLGEIPKTDTSSESQLFQSSLRSHTDATPDIANTTGYVGSNERNTTNDDDWIRLLLDEGFI

>AtMYB81

MGKVRQDSGSDDDNSIKKSFTKGPWTQAEDNLLIAYVDKHGDGNWNAVQNNSGLSRCGKSCRLRWVNHLRPDLKKGAFTEKEEKRVIELHALLGNKWARMAEELPGRTDNEIKNFWNTRLKRLQRLGLPVYPDEVREHAMNAATHSGLNTDSLDGHHSQEYMEADTVEIPEVDFEHLPLNRSSSYYQSMLRHVPPTNVFVRQKPCFFQPPNVYNLIPPSPYMSTGKRPREPETAFPCPGGYTMNEQSPRLWNYPFVENVSEQLPDSHLLGNAAYSSPPGPLVHGVENFEFPSFQYHEEPGGWGADQPNPMPEHESDNTLVQSPLTAQTPSDCPSSSLYDGLLESVVYGSSGEKPATDTDSESSLFQSFTPANENITGKTCFLTLYALHALHCLCNQFKKSPLLHLHDKLNWCNKFRFNSFKSGTHIL

>AtMYB65

MSYTTATADSDDGMHSSIHNESPAPDSISNGCRSRGKRSVLKKGPWTSTEDGILIDYVKKHGEGNWNAVQKHTSLARCGKSCRLRWANHLRPNLKKGAFSQEEEQLIVEMHAKMGNKWAQMAEHLPGRTDNEIKNYWNTRIKRRQRAGLPLYPPEIYVDDLHWSEEYTKSNIIRVDRRRRHQDFLQLGNSKDNVLFDDLNFAASLLPAASDLSDLVACNMLGTGASSSRYESYMPPILPSPKQIWESGSRFPMCSSNIKHEFQSPEHFQNTAVQKNPRSCSISPCDVDHHPYENQHSSHMMMVPDSHTVTYGMHPTSKPLFGAVKLELPSFQYSETSAFDQWKTTPSPPHSDLLDSVDAYIQSPPPSQVEESDCFSSCDTGLLDMLLHEAKIKTSAKHSLLMSSPQKSFSSTTCTTNVTQNVPRGSENLIKSGEYEDSQKYLGRSEITSPSQLSAGGFSSGELIKVTC

>AtMYB33

MSYTSTDSDHNESPAADDNGSDCRSRWDGHALKKGPWSSAEDDILIDYVNKHGEGNWNAVQKHTSLFRCGKSCRLRWANHLRPNLKKGAFSQEEEQLIVELHAKMGNRWARMAAHLPGRTDNEIKNYWNTRIKRRQRAGLPLYPPEMHVEALEWSQEYAKSRVMGEDRRHQDFLQLGSCESNVFFDTLNFTDMVPGTFDLADMTAYKNMGNCASSPRYENFMTPTIPSSKRLWESELLYPGCSSTIKQEFSSPEQFRNTSPQTISKTCSFSVPCDVEHPLYGNRHSPVMIPDSHTPTDGIVPYSKPLYGAVKLELPSFQYSETTFDQWKKSSSPPHSDLLDPFDTYIQSPPPPTGGEESDLYSNFDTGLLDMLLLEAKIRNNSTKNNLYRSCASTIPSAD

LGQVTVSQTKSEEFDNSLKSFLVHSEMSTQNADETPPRQREKKRKPLLDITRPDVLLASSWLDHGLGIVKETGSMSDALAVLLGDDIGNDYMNMSVGASSGVGSCSWSNMPPVCQMTELP

>AtMYB125

MRKMEAKKEEIKKGPWKAEEDEVLINHVKRYGPRDWSSIRSKGLLQRTGKSCRLRWVNKLRPNLKNGCKFSADEERTVIELQSEFGNKWARIATYLPGRTDNDVKNFWSSRQKRLARILHNSSDASSSSFNPKSSSSHRLKGKNVKPIRQSSQGFGLVEEEVTVSSSCSQMVPYSSDQVGDEVLRLPDLGVKLEHQPFAFGTDLVLAEYSDSQNDANQQAISPFSPESRELLARLDDPFYYDILGPADSSEPLFALPQPFFEPSPVPRRCRHVSKDEEADVFLDDFPADMFDQVDPIPSP

>AtMYB98

MENFVDENGFASLNQNIFTRDQEHMKEEDFPFEVVDQSKPTSFLQDFHHLDHDHQFDHHHHHGSSSSHPLLSVQTTSSCINNAPFEHCSYQENMVDFYETKPNLMNHHHFQAVENSYFTRNHHHHQEINLVDEHDDPMDLEQNNMMMMRMIPFDYPPTETFKPMNFVMPDEISCVSADNDCYRATSFNKTKPFLTRKLSSSSSSSSWKETKKSTLVKGQWTAEEDRVLIQLVEKYGLRKWSHIAQVLPGRIGKQCRERWHNHLRPDIKKETWSEEEDRVLIEFHKEIGNKWAEIAKRLPGRTENSIKNHWNATKRRQFSKRKCRSKYPRPSLLQDYIKSLNMGALMASSVPARGRRRESNNKKKDVVVAVEEKKKEEEVYGQDRIVPECVFTDDFGFNEKLLEEGCSIDSLLDDIPQPDIDAFVHGL

>AtMYB119

MEDRRLVHGAAPPLTAVERFLYGQKNDALCSKKQESSRDQPIVKTKISIETRSDNKENTTFGPTREKHLVLNGGNRNPTGEVVARSAARDYQNSTKKRSSKNLIKGQWTAEEDRKLIRLVRQHGERKWAMISEKLEGRAGKQCRERWHNHLRPDIKKDGWSEEEERVLVESHMRIGNKWAEIAKLIPGRTENSIKNHWNATKRRQNSKRKHKRESNADNNDRDASPSAKRPCILQDYIKSIERNNINKDNDEKKNENTISVISTPNLDQIYSDGDSASSILGGPYDEELDYFQNIFANHPISLENLGLSQTSDEVTQSSSSGFMIKNPNPNLHDSVGIHHQEATITAPANTPHLASDIYLSYLLNGTTSSYSDTHFPSSSSSTSSTTVEHGGHNEFLEPQANSTSERREMDLIEMLSGSIQGSNICFPLV

>AtMYB64

MEEQKIQEKSLAHGAAPPLTAVERFLNGQKNEALCFKKQERSIDRPIVKTTRAIEIRNENKENMMFGPRKEKNLAVIGEIVVKGAAKDYTCKDITKKQPYKNIIKGQWTADEDRKLIKLVMQHGERKWAVISEKLEGRAGKQCRERWHNHLRPDIKKDSWSEEEERLLVEAHTRIGNKWAEIAKLIQGRTENSIKNHWNATKRRQNSKRKHKRSKNADSNSDIDDLSPSAKRPRILEDYIKNIENNDKNNGENIMTTSGNNVLSTSNYDQFNSEDSTSSLLDDPYDEELVFLKNIFENHSLENINLSQGTEITQSSSSGFMIENPKPKPNLYNNTFGTHLGAMVTEPANSSHLASDIYLSDLLNGTASSSSSLTFLSSNNNEHAGENELLLPQANSTSERREMDLIEMLSGSTQGSNIWFPLF

>AtMYB118

MEFESVFKMHYPYLAAVIYDDSSTLKDFHPSLTDDFSCVHNVHHKPSMPHTYEIPSKETIRGITPSPCTEAFEACFHGTSNDHVFFGMAYTTPPTIEPNVSHVSHDNTMWENDQNQGFIFGTESTLNQAMADSNQFNMPKPLLSANEDTIMNRRQNNQVMIKTEQIKKKNKRFQMRRICKPTKKASIIKGQWTPEEDKLLVQLVDLHGTKKWSQIAKMLQGRVGKQCRERWHNHLRPDIKKDGWTEEEDIILIKAHKEIGNRWAEIARKLPGRTENTIKNHWNATKRRQHSRRTKGKDEISLSLGSNTLQNYIRSVTYNDDPFMTANANANIGPRNMRGKGKNVMVAVSEYDEGECKYIVDGVNNLGLEDGRIKMPSLAAMSASGSASTSGSASGSGSGVTMEIDEPMTDSWMVMHGCDEVMMNEIALLEMIAHGRL

>AtMYB115

MYHQNLISSTPNQNSNPHDWDIQNPLFSIHPSAEIPSKYPFMGITSCPNTNVFEEFQYKITNDQNFPTTYNTPFPVISEGISYNMHDVQENTMCGYTAHNQGLIIGCHEPVLVHAVVESQQFNVPQSEDINLVSQSERVTEDKVMFKTDHKKKDIIGKGQWTPTEDELLVRMVKSKGTKNWTSIAKMFQGRVGKQCRERWHNHLRPNIKKNDWSEEEDQILIEVHKIVGNKWTEIAKRLPGRSENIVKNHWNATKRRLHSVRTKRSDAFSPRNNALENYIRSITINNNALMNREVDSITANSEIDSTRCENIVDEVMNLNLHATTSVYVPEQAVLTWGYDFTKCYEPMDDTWMLMNGWN

>AtMYB100

MKKNYQKKNIKVVSTSKYLKKSDIDKVNWTESEDIKLKEIMALGPKNKWTKVAKKFEGRTGKQCRERWYNHARPNIKKTAWSEEEDQILIEAHKVLGTKWVEIAQQLPGRSDNNIKNHWNTTKRRVQNKRGGTVNPVGNNILENYIRCITINNEDFLKTDGSYGEPTNIESDDDSKDMLYGEMNLSLETITTQTTKPLTNASTTSPYVQMPEDNYTMEDCESLEDILELLRWWE

>AtMYB22

MGEPQLFDVPVLEGIKNTTNEIMNQLEDDKMKKTYENKKEASTSKYLKKSDITKKRWTESEDIKLKEMVALEPKKWTKVAKHFEGRTPKQCRERWHNHARPNVKKTTWSEEEDQILIEVHKVIGAKWIQISEQLPGRSYNNVKNHWNTTKRRVQNKSGRTVNRVGNNILENYIRSITINNDDESDGEPTNIENYHDDSEDMLYGEMNLSPEAITQTTKPLTDASTISPYIPMPKENYTLEVCESLEDYLELLRWWD

>AtMYB124

MEDTKKKKKKNINNNQDSKKKERHIVTWSQEEDVILREQITLHGTENWAIIASKFKDKSTRQCRRRWYTYLNSDFKRGGWSPEEDMLLCEAQRVFGNRWTEIAKVVSGRTDNAVKNRFTTLCKKRAKHEAMTKDSNSNTKRMLFLDGISTPRKSENETPIAKKLKRSHILDLTEISNYGRAEACVNQQIRSPFSVLARNATGIDSLEEQNQTSNVNESDGEGMFLKKDDPKVTALMQQAELLSSLAQKVNADNTEQSMENAWKVLQDFLNKGKENDLFRYGIPDIDFKIEEFKDLIEDLRSGYEDNQLSWRQPDLHDSPASSEYSSGSTIMVDQSGDKTQPFSADTQTEHKQVGEELLVPKNPDENMPISGEEKFSSPIQVTPLFRSLADGIPSPQFSESERSFLLKTLGIESSSPCPSANPSKPPPCKRVLLHSL

>AtMYB88

MEETTKQNNMKKKKKILLHSDDSKKKERHIVTWSPEEDDILRKQISLQGTENWAIIASKFNDKSTRQCRRRWYTYLNSDFKRGGWSPEEDTLLCEAQRLFGNRWTEIAKVVSGRTDNAVKNRFTTLCKKRAKHEAMAKENRIACCVNSDNKRLLFPDGISTPLKAESESPLTKKMRRSHIPNLTEIKSYGDRSHIKVESTMNQQRRHPFSVVAHNATSSDGTEEQKQIGNVKESDGEDKSNQEVFLKKDDSKVTALMQQAELLSSLAQKVNADNTDQSMENAWKVLQDFLNKSKENDLFRYGIPDIDFQLDEFKDLVEDLRSSNEDSQSSWRQPDLHDSPASSEYSSGSGSGSTIMTHPSGDKTQQLMSDTQTTSHQQNGGELLQDNGIVSDATVEQVGLLSTGHDVLKNSNETVPIPGEEEFNSPVQVTPLFRSLAAGIPSPQFSESERNFLLKTLGVESPSPYPSANPSQPPPCKRVLLDSL

>AtMYB91

MKERQRWSGEEDALLRAYVRQFGPREWHLVSERMNKPLNRDAKSCLERWKNYLKPGIKKGSLTEEEQRLVIRLQEKHGNKWKKIAAEVPGRTAKRLGKWWEVFKEKQQREEKESNKRVEPIDESKYDRILESFAEKLVKERSNVVPAAAAAATVVMANSNGGFLHSEQQVQPPNPVIPPWLATSNNGNNVVARPPSVTLTLSPSTVAAAAPQPPIPWLQQQQPERAENGPGGLVLGSMMPSCSGSSESVFLSELVECCRELEEGHRAWADHKKEAAWRLRRLELQLESEKTCRQREKMEEIEAKMKALREEQKNAMEKIEGEYREQLVGLRRDAEAKDQKLADQWTSRHIRLTKFLEQQMGCRLDRP

>AtMYB89

MYLFMYKCNIVLEETHVFQNTPCDVSLQRPFNGFGENNALPLRKMHQEEKKKKHRGGHWTLSEDLKLKELVAVFGPQNWKFIGEKMEPRTSLSCRQRWFNQLDPKINKRNFTDEEEEKLLRAHILYGNKWSKIAKLFNRRTDHAVKNHWHSLMNRIIRKQSASDIRSFDNIQNYQTSNFLPGLCLLNTKQ

>AtMYB110

MKMDFSCFQEYPFEFHCRGTTFNGFRENNAVSETVEEFCNKRRMQKKSDDLKTKKKKKQSVSRVCSRGHWRISEDTQLMELVSVYGPQNWNHIAESMQGRTGKSCRLRWFNQLDPRINKRAFSDEEEERLLAAHRAFGNKWAMIAKLFNGRTDNALKNHWHVLMARKMRQQSSSYVQRFNGSAHESNTDHKIFNLSPGNVDDDEDVNLKKCSWEMLKEGTTNLKAQYLQEEYSSSRMPMQGPHHHYSTFPADSLALTLHVSIQEPSSSSSLSLPSSSTTGEHTMVTRYFETIKPPAFIDFLGVGH

>AtMYB117

MFITEKQVWMDEIVARRASSSWDFPFNDINIHQHHHRHCNTSHEFEILKSPLGDVAVHEEESNNNNPNFSNSESGKKETTDSGQSWSSSSSKPSVLGRGHWRPAEDVKLKELVSIYGPQNWNLIAEKLQGRSGKSCRLRWFNQLDPRINRRAFTEEEEERLMQAHRLYGNKWAMIARLFPGRTDNSVKNHWHVVMARKYREHSSAYRRRKLMSNNPLKPHLTNNHHPNPNPNYHSFISTNHYFAQPFPEFNLTHHLVNNAPITSDHNQLVLPFHCFQGYENNEPPMVVSMFGNQMMVGDNVGATSDALCNIPHIDPSNQEKPEPNDAMHWIGMDAVDEEVFEKAKQQPHFFDFLGLGTA

>AtMYB105

MMFKVIFNICSSPPMRMEMVHADVASLSITPCFPSSLSSSSHHHYNQQQHCIMSEDQHHSMDQTTSSDYFSLNIDNAQHLRSYYTSHREEDMNPNLSDYS

NCNKKDTTVYRSCGHSSKASVSRGHWRPAEDTKLKELVAVYGPQNWNLIAEKLQGRSGKSCRLRWFNQLDPRINRRAFTEEEEERLMQAHRLYGNKWAMIARLFPGRTDNSVKNHWHVIMARKFREQSSSYRRRKTMVSLKPLINPNPHIFNDFDPTRLALTHLASSDHKQLMLPVPCFPGYDHENESPLMVDMFETQMMVGDYIAWTQEATTFDFLNQTGKSEIFERINEEKKPPFFDFLGLGTV

>AtMYB69

MEMSRGSNSFDNKKPSCQRGHWRPVEDDNLRQLVEQYGPKNWNFIAQHLYGRSGKSCRLRWYNQLDPNITKKPFTEEEEERLLKAHRIQGNRWASIARLFPGRTDNAVKNHFHVIMARRKRENFSSTATSTFNQTWHTVLSPSSSLTRLNRSHFGLWRYRKDKSCGLWPYSFVSPPTNGQFGSSSVSNVHHEIYLERRKSKELVDPQNYTFHAATPDHKMTSNEDGPSMGDDGEKNDVTFIDFLGVGLAS

>AtMYB56

MNPNLLEKDLRGKETTNGSIRYKEANNFRSLPNSHTAACKTSLNNPSISRNHPHNKSASVLESEDEHGNERGENEKSLRMRGKSGINTKVCSRGHWRPTEDAKLKELVAQFGPQNWNLISNHLLGRSGKSCRLRWFNQLDPRINKRAFTEEEEFRLLAAHRAYGNKWALISRLFPGRTDNAVKNHWHVIMARRTRESQRQRQQPPPTLSRDAEMTVSSSCRYNQGKFINEEDDDDDVSAVSTCTTELSLTPPSSAYQPRFFNYDSTLASGKDGQCVQRAEVNGIYGKKMDHQNHHTISVSERKVEMKMRSGYYYFDFLGVGAS

>AtMYB54

MIMCSRGHWRPAEDEKLKDLVEQYGPHNWNAIALKLPGRSGKSCRLRWFNQLDPRINRNPFTEEEEERLLAAHRIHGNRWSIIARLFPGRTDNAVKNHWHVIMARRTRQTSKPRLLPSTTSSSSLMASEQIMMSSGGYNHNYSSDDRKKIFPADFINFPYKFSHINHLHFLKEFFTGKIALNHKANQSKKPMEFYNFLQVNTDSNKSEIIDQDSGQSKRSDSDTKHESHVPFFDFLSVGNSAS

>AtMYB52

MMCSRGHWRPAEDEKLRELVEQFGPHNWNAIAQKLSGRSGKSCRLRWFNQLDPRINRNPFTEEEEERLLASHRIHGNRWSVIARFFPGRTDNAVKNHWHVIMARRGRERSKLRPRGLGHDGTVAATGMIGNYKDCDKERRLATTTAINFPYQFSHINHFQVLKEFLTGKIGFRNSTTPIQEGAIDQTKRPMEFYNFLQVNTDSKIHELIDNSRKDEEEDVDQNNRIPNENCVPFFDFLSVGNSASQGLC

>AtMYB73

MSNPTRKNMERIKGPWSPEEDDLLQRLVQKHGPRNWSLISKSIPGRSGKSCRLRWCNQLSPEVEHRAFSQEEDETIIRAHARFGNKWATISRLLNGRTDNAIKNHWNSTLKRKCSVEGQSCDFGGNGGYDGNLGEEQPLKRTASGGGGVSTGLYMSPGSPSGSDVSEQSSGGAHVFKPTVRSEVTASSSGEDPPTYLSLSLPWTDETVRVNEPVQLNQNTVMDGGYTAELFPVRKEEQVEVEEEEAKGISGGFGGEFMTVVQEMIRTEVRSYMADLQRGNVGGSSSGGGGGGSCMPQSVNSRRVGFREFIVNQIGIGKME

>AtMYB70

MSGSTRKEMDRIKGPWSPEEDDLLQSLVQKHGPRNWSLISKSIPGRSGKSCRLRWCNQLSPEVEHRGFTAEEDDTIILAHARFGNKWATIARLLNGRTDNAIKNHWNSTLKRKCSGGGGGGEEGQSCDFGGNGGYDGNLTDEKPLKRRASGGGGVVVVTALSPTGSDVSEQSQSSGSVLPVSSSCHVFKPTARAGGVVIESSSPEEEEKDPMTCLRLSLPWVNESTTPPELFPVKREEEEEKEREISGLGGDFMTVVQEMIKTEVRSYMADLQLGNGGGAGGGASSCMVQGTNGRNVGFREFIGLGRIE

>AtMYB77

MADRVKGPWSQEEDEQLRRMVEKYGPRNWSAISKSIPGRSGKSCRLRWCNQLSPEVEHRPFSPEEDETIVTARAQFGNKWATIARLLNGRTDNAVKNHWNSTLKRKCSGGVAVTTVTETEEDQDRPKKRRSVSFDSAFAPVDTGLYMSPESPNGIDVSDSSTIPSPSSPVAQLFKPMPISGGFTVVPQPLPVEMSSSSEDPPTSLSLSLPGAENTSSSHNNNNNALMFPRFESQMKINVEERGEGRRGEFMTVVQEMIKAEVRSYMAEMQKTSGGFVVGGLYESGGNGGFRDCGVITPKVE

>AtMYB44

MADRIKGPWSPEEDEQLRRLVVKYGPRNWTVISKSIPGRSGKSCRLRWCNQLSPQVEHRPFSAEEDETIARAHAQFGNKWATIARLLNGRTDNAVKNHWNSTLKRKCGGYDHRGYDGSEDHRPVKRSVSAGSPPVVTGLYMSPGSPTGSDVSDSSTIPILPSVELFKPVPRPGAVVLPLPIETSSSSDDPPTSLSLSLPGADVSEESNRSHESTNINNTTSSRHNHNNTVSFMPFSGGFRGAIEEMGKSFPGNGGEFMAVVQEMIKAEVRSYMTEMQRNNGGGFVGGFIDNGMIPMSQIGVGRIE

>AtMYB1

MEAEIVRRSEVTGLRREVEESSIGRGDCDGDGGDVGEDAAGFVGTSGRGRRDRVKGPWSKEEDDVLSELVKRLGARNWSFIARSIPGRSGKSCRLRWCNQLNPNLIRNSFTEVEDQAIIAAHAIHGNKWAVIAKLLPGRTDNAIKNHWNSALRRRFIDFEKAKNIGTGSLVVDDSGFDRTTTVASSEETLSSGGGCHVTTPIVSPEGKEATTSMEMSEEQCVEKTNGEGISRQDDKDPPTLFRPVPRLSSFNACNHMEGSPSPHIQDQNQLQSSKQDAAMLRLLEGAYSERFVPQTCGGGCCSNNPDGSFQQESLLGPEFVDYLDSPTFPSSELAAIATEIGSLAWLRSGLESSSVRVMEDAVGRLRPQGSRGHRDHYLVSEQGTNITNVLST

>AtMYB109

MEGETHQSEPLPLASGDSDEGISAAIEAELAELAAGDSSGGGGCGGGGGGIRSKVKGPWSTEEDAVLTKLVRKLGPRNWSLIARGIPGRSGKSCRLRWCNQLDPCLKRKPFSDEEDRMIISAHAVHGNKWAVIAKLLTGRTDNAIKNHWNSTLRRKYADLWNNGQWMANSVTTASVKNENVDETTNPPSSKQQLPQGDINSSPPKPPQVSDVVMEEAANEPQEPQEQQEQAPPVVSNVPTENNVFRPVARVGAFSIYNPTSQKNGYRDYNIVPCEGPLIQAAKPDSLAGKFLQSLCDEPQIPSKCGHGCSTLPAETKFSRNSVLGPEFVDYEEPSAVFNQELISIATDLNNIAWIKSGLDNAVVREAEQSLKMDNYNYNDPRIKFTGMMPRQDFFCARS

>AtMYB25

MNGEISRPPELISSRNPCKSFENAIHKAVEAELAELAKSDANGGGKSKVKGPWLPEQDEALTRLVKMCGPRNWNLISRGIPGRSGKSCRLRWCNQLDPILKRKPFSDEEEHMIMSAQAVLGNKWSVIAKLLPGRTDNAIKNHWNSNLRRKPAEQWKIPLLMSNTEIVYQLYPSMVRRISNASPKEHLPQEEETGVLSDDKMDDEAKEPPREQNSKTGVYRPVARMGAFSVCKPGYMAPCEGPLVQASRPDSLAGKFLQSLCYDPIIPSKCGHGCCNHQDSTTLSSSSVLGSEFVDYEEHSSAELDKELISISNDLNNTAWIRSGKEAEQSLKADDQFRREYAHSKFSGMVNNGVSSQMVRQDLRALS

>ppa009439m

MGRAPCCSKVGLHRGPWTPREDTLLTKYIEAHGEGHWRSMPKKAGLLRCGKSCRLRWMNYLRPDIKRGNITPDEDDLIVR

LHSLLGNRWSLIAGRLPGRTDNEIKNYWNTHLSKKLRSQGTDPSTHKKLSEPVVKENKRRKNQKTKNNMNKKEMVVKNKN

KTGQHVEPLKPKVHLPKPTRVTSFLSLQRNDSFTSSTTTTTTTTSSQDLKGGGGAFGINENDQVLVNTRANGIVFCVGDD

QDQDQVPNSADDNDHTLENLYEEYLQALLKTDHHHDHQNQLELDSFAESLLI

>ppa010846m

MRKPCCDKQDTNRGAWSKQEDLKLIDYIRKHGEGCWRTLPQAAGLLRCGKSCRLRWINYLRPDLKRGNFAEDEEDLIVKL

HALLGNRWSLIAGRLPGRTDNEVKNYWNSHLRRKLISMGIDPNNHRPNTFNLPRPHHKNSQAISSTAKLSAGLKTPTNDQ

PARSGGNNCDQVSDGTSCLEDESCGHQLPDLNLDLTMTAPLSNSEPENLKEEQNLMSLKCHRNLPRPPISFLS

>ppa007753m

MEMKERQRWRAEEDALLRAYVQQYGPREWNLVSQRMNTPLNRDAKSCLERWKNYLKPGIKKGSLTEEEQRLVICLQAKHG

NKWKKIAAEVPGRTAKRLGKWWEVFKEKQQREQKNNKTVDPVDEGKYDRILETFAEKLVQERAAPSYLMATSNGSYLHTE

TSSPAPTMLPPWLSNSNVTSNVRPPSPSVTLSLSPTVAPSPPIPWLQPDRGSDNSIVLGNMPHHGSVPVCGENLVISELV

ECSRELEEVHRAWAAHKKEASWRLRRVELQLESEKACRRREKMEEIEAKVKALREEQKAALDRIEAEYREQLAGLRRDAE

AKEQKLTEQWVAKHLRLSKFLDQMGGRPRLAEPNGR

>GmMYB3a

MRKPSCDIKELNKGAWSKQEDQKLVDYIKKHGEVCWRTLPQAAGLHRCGKSCRLRWINYLRPDLKRGNFAEDEEDLIIKLHALLGNRWSLIAGRLPGRTDNEVKNYWNSHIRKKLISNGIDPNNHRLNHTIPSVYQNPPMSDDSSKHFGMKDNNSKNETSKSPRVYNHGEVSDAASGEAESSCALPDLNLDLSICEKNLKPFHESKLSRKVPFDSPSTLLLFQ

**WRKY**

>AtWRKY70

MDTNKAKKLKVMNQLVEGHDLTTQLQQLLSQPGSGLEDLVAKILVCFNNTISVLDTFEPISSSSSLAAVEGSQNASCDNDGKFEDSGDSRKRLGPVKGKRGCYKRKKRSETCTIESTILEDAFSWRKYGQKEILNAKFPRSYFRCTHKYTQGCKATKQVQKVELEPKMFSITYIGNHTCNTNAETPKSKTCDHHDEIFMDSEDHKSPSLSTSMKEEDNPHRHHGSSTENDLSLVWPEMVFEEDYHHQASYVNGKTSTSIDVLGSQDLMVFGGGGDFEFSENEHFSIFSSCSNLS

>AtWRKY33

MAASFLTMDNSRTRQNMNGSANWSQQSGRTSTSSLEDLEIPKFRSFAPSSISISPSLVSPSTCFSPSLFLDSPAFVSSSANVLASPTTGALITNVTNQKGINEGDKSNNNNFNLFDFSFHTQSSGVSAPTTTTTTTTTTTTTNSSIFQSQEQQKKNQSEQWSQTETRPNNQAVSYNGREQRKGEDGYNWRKYGQKQVKGSENPRSYYKCTFPNCPTKKKVERSLEGQITEIVYKGSHNHPKPQSTRRSSSSSSTFHSAVYNASLDHNRQASSDQPNSNNSFHQSDSFGMQQEDNTTSDSVGDDEFEQGSSIVSRDEEDCGSEPEAKRWKGDNETNGGNGGGSKTVREPRIVVQTTSDIDILDDGYRWRKYGQKVVKGNPNPRSYYKCTTIGCPVRKHVERASHDMRAVITTYEGKHNHDVPAARGSGYATNRAPQDSSSVPIRPAAIAGHSNYTTSSQAPYTLQMLHNNNTNTGPFGYAMNNNNNNSNLQTQQNFVGGGFSRAKEEPNEETSFFDSFMP

>AtWRKY46

MMMEEKLVINELELGKELANRLMNNLKHTSSVDSNKTLISDILRIYQNAIFMLSFNQDKNILKRSLEIDGKDSKNVFKKRKVSEKNTEKVKVFVATEQENGSIDDGHCWRKYGQKEIHGSKNPRAYYRCTHRFTQDCLAVKQVQKSDTDPSLFEVKYLGNHTCNNITSPKTTTNFSVSLTNTNIFEGNRVHVTEQSEDMKPTKSEEVMISLEDLENKKNIFRTFSFSNHEIENGVWKSNLFLGNFVEDLSPATSGSAITSEVLSAPAAVENSETADSYFSSLDNIIDFGQDWLWS

>AtWRKY54

MDSNSNNTKSIKRKVVDQLVEGYEFATQLQLLLSHQHSNQYHIDETRLVSGSGSVSGGPDPVDELMSKILGSFHKTISVLDSFDPVAVSVPIAVEGSWNASCGDDSATPVSCNGGDSGESKKKRLGVGKGKRGCYTRKTRSHTRIVEAKSSEDRYAWRKYGQKEILNTTFPRSYFRCTHKPTQGCKATKQVQKQDQDSEMFQITYIGYHTCTANDQTHAKTEPFDQEIIMDSEKTLAASTAQNHVNAMVQEQENNTSSVTAIDAGMVKEEQNNNGDQSKDYYEGSSTGEDLSLVWQETMMFDDHQNHYYCGETSTTSHQFGFIDNDDQFSSFFDSYCADYERTSAM

>AtWRKY53

MEGRDMLSWEQKTLLSELINGFDAAKKLQARLREAPSPSSSFSSPATAVAETNEILVKQIVSSYERSLLLLNWSSSPSVQLIPTPVTVVPVANPGSVPESPASINGSPRSEEFADGGGSSESHHRQDYIFNSKKRKMLPKWSEKVRISPERGLEGPQDDVFSWRKYGQKDILGAKFPRSYYRCTHRSTQNCWATKQVQRSDGDATVFEVTYRGTHTCSQAITRTPPLASPEKRQDTRVKPAITQKPKDILESLKSNLTVRTDGLDDGKDVFSFPDTPPFYNYGTINGEFGHVESSPIFDVVDWFNPTVEIDTTFPAFLHESIYY

>AtWRKY1

MAEVGKVLASDMELDHSNETKAVDDVVATTDKAEVIPVAVTRTETVVESLESTDCKELEKLVPHTVASQSEVDVASPVSEKAPKVSESSGALSLQSGSEGNSPFIREKVMEDGYNWRKYGQKLVKGNEFVRSYYRCTHPNCKAKKQLERSAGGQVVDTVYFGEHDHPKPLAGAVPINQDKRSDVFTAVSKGEQRIDIVSLIYKLCIVSYDIMFVEKTSGSSVQTLRQTEPPKIHGGLHVSVIPPADDVKTDISQSSRITGDNTHKDYNSPTAKRRKKGGNIELSPVERSTNDSRIVVHTQTLFDIVNDGYRWRKYGQKSVKGSPYPRSYYRCSSPGCPVKKHVERSSHDTKLLITTYEGKHDHDMPPGRVVTHNNMLDSEVDDKEGDANKTPQSSTLQSITKDQHVEDHLRKKTKTNGFEKSLDQGPVLDEKLKEEIKERSDANKDHAANHAKPEAKSDDKTTVCQEKAVGTLESEEQKPKTEPAQS

>AtWRKY4

MSEKEEAPSTSKSTGAPSRPTLSLPPRPFSEMFFNGGVGFSPGPMTLVSNMFPDSDEFRSFSQLLAGAMSSPATAAAAAAAATASDYQRLGEGTNSSSGDVDPRFKQNRPTGLMISQSQSPSMFTVPPGLSPAMLLDSPSFLGLFSPVQGSYGMTHQQALAQVTAQAVQANANMQPQTEYPPPSQVQSFSSGQAQIPTSAPLPAQRETSDVTIIEHRSQQPLNVDKPADDGYNWRKYGQKQVKGSEFPRSYYKCTNPGCPVKKKVERSLDGQVTEIIYKGQHNHEPPQNTKRGNKDNTANINGSSINNNRGSSELGASQFQTNSSNKTKREQHEAVSQATTTEHLSEASDGEEVGNGETDVREKDENEPDPKRRSTEVRISEPAPAASHRTVTEPRIIVQTTSEVDLLDDGYRWRKYGQKVVKGNPYPRSYYKCTTPGCGVRKHVERAATDPKAVVTTYEGKHNHDLPAAKSSSHAAAAAQLRPDNRPGGLANLNQQQQQQPVARLRLKEEQTT

>AtWRKY25

MSSTSFTDLLGSSGVDCYEDDEDLRVSGSSFGGYYPERTGSGLPKFKTAQPPPLPISQSSHNFTFSDYLDSPLLLSSSHSLISPTTGTFPLQGFNGTTNNHSDFPWQLQSQPSNASSALQETYGVQDHEKKQEMIPNEIATQNNNQSFGTERQIKIPAYMVSRNSNDGYGWRKYGQKQVKKSENPRSYFKCTYPDCVSKKIVETASDGQITEIIYKGGHNHPKPEFTKRPSQSSLPSSVNGRRLFNPASVVSEPHDQSENSSISFDYSDLEQKSFKSEYGEIDEEEEQPEMKRMKREGEDEGMSIEVSKGVKEPRVVVQTISDIDVLIDGFRWRKYGQKVVKGNTNPRSYYKCTFQGCGVKKQVERSAADERAVLTTYEGRHNHDIPTALRRS

>AtWRKY52

MTNCEKDEEFVCISCVEEVRYSFVSHLSEALRRKGINNVVVDVDIDDLLFKESQAKIEKAGVSVMVLPGNCDPSEVWLDKFAKVLECQRNNKDQAVVSVLYGDSLLRDQWLSELDFRGLSRIHQSRKECSDSILVEEIVRDVYETHFYVGRIGIYSKLLEIENMVNKQPIGIRCVGIWGMPGIGKTTLAKAVFDQMSSAFDASCFIEDYDKSIHEKGLYCLLEEQLLPGNDATIMKLSSLRDRLNSKRVLVVLDDVRNALVGESFLEGFDWLGPGSLIIITSRDKQVFCLCGINQIYEVQGLNEKEARQLFLLSASIKEDMGEQNLQELSVRVINYANGNPLAISVYGRELKGKKKLSEMETAFLKLKRRPPFKIVDAFKSTYDTLSDNEKNIFLDIACFFQGENVNYVIQLLEGCGFFPHVEIDVLVDKCLVTISENRVWLHKLTQDIGREIINGETVQIERRRRLWEPWSIKYLLEYNEHKANGEPKTTFKRAQGSEEIEGLFLDTSNLRFDLQPSAFKNMLNLRLLKIYCSNPEVHPVINFPTGSLHSLPNELRLLHWENYPLKSLPQNFDPRHLVEINMPYSQLQKLWGGTKNLEMLRTIRLCHSHHLVDIDDLLKAENLEVIDLQGCTRLQNFPAAGRLLRLRVVNLSGCIKIKSVLEIPPNIEKLHLQGTGILALPVSTVKPNHRELVNFLTEIPGLSEELERLTSLLESNSSCQDLGKLICLELKDCSCLQSLPNMANLDLNVLDLSGCSSLNSIQGFPRFLKQLYLGGTAIREVPQLPQSLEILNAHGSCLRSLPNMANLEFLKVLDLSGCSELETIQGFPRNLKELYFAGTTLREVPQLPLSLEVLNAHGSDSEKLPMHYKFNNFFDLSQQVVNDFLLKTLTYVKHIPRGYTQELINKAPTFSFSAPSHTNQNATFDLQSGSSVMTRLNHSWRNTLVGFGMLVEVAFPEDYCDATDVGISCVCRWSNKEGRSCRIERKFHCWAPWQVVPKVRKDHTFVFSDVNMRPSTGEGNDPDIWAGLVVFEFFPINQQTKCLNDRFTVRRCGVRVINVATGNTSLENIALVLSLDPVEVSGYEVLRVSYDDLQEMDKVLFLYIASLFNDEDVDFVAPLIAGIDLDVSSGLKVLADVSLISVSSNGEIVMHSLQRQMGKEILHGQSMLLSDCESSMTENLSDVPKKKKKHSESRVKKVVSIPAIDEGDLWTWRKYGQKDILGSRFPRGYYRCAYKFTHGCKATKQVQRSETDSNMLAITYLSEHNHPRPTKRKALADSTRSTSSSIC

>AtWRKY8

MSHEIKDLNNYHYTSSYNHYNINNQNMINLPYVSGPSAYNANMISSSQVGFDLPSKNLSPQGAFELGFELSPSSSDFFNPSLDQENGLYNAYNYNSSQKSHEVVGDGCATIKSEVRVSASPSSSEADHHPGEDSGKIRKKREVRDGGEDDQRSQKVVKTKKKEEKKKEPRVSFMTKTEVDHLEDGYRWRKYGQKAVKNSPYPRSYYRCTTQKCNVKKRVERSYQDPTVVITTYESQHNHPIPTNRRTAMFSGTTASDYNPSSSPIFSDLIINTPRSFSNDDLFRVPYASVNVNPSYHQQQHGFHQQESEFELLKEMFPSVFFKQEP

>AtWRKY11

MAVDLMRFPKIDDQTAIQEAASQGLQSMEHLIRVLSNRPEQQHNVDCSEITDFTVSKFKTVISLLNRTGHARFRRGPVHSTSSAASQKLQSQIVKNTQPEAPIVRTTTNHPQIVPPPSSVTLDFSKPSIFGTKAKSAELEFSKENFSVSLNSSFMSSAITGDGSVSNGKIFLASAPLQPVNSSGKPPLAGHPYRKRCLEHEHSESFSGKVSGSAYGKCHCKKSRKNRMKRTVRVPAISAKIADIPPDEYSWRKYGQKPIKGSPHPRGYYKCSTFRGCPARKHVERALDDPAMLIVTYEGEHRHNQSAMQENISSSGINDLVFASA

>AtWRKY18

MDGSSFLDISLDLNTNPFSAKLPKKEVSVLASTHLKRKWLEQDESASELREELNRVNSENKKLTEMLARVCESYNELHNHLEKLQSRQSPEIEQTDIPIKKRKQDPDEFLGFPIGLSSGKTENSSSNEDHHHHHQQHEQKNQLLSCKRPVTDSFNKAKVSTVYVPTETSDTSLTVKDGFQWRKYGQKVTRDNPSPRAYFRCSFAPSCPVKKKVQRSAEDPSLLVATYEGTHNHLGPNASEGDATSQGGSSTVTLDLVNGCHRLALEKNERDNTMQEVLIQQMASSLTKDSKFTAALAAAISGRLMEQSRT

>AtWRKY6

MDRGWSGLTLDSSSLDLLNPNRISHKNHRRFSNPLAMSRIDEEDDQKTRISTNGSEFRFPVSLSGIRDREDEDFSSGVAGDNDREVPGEVDFFSDKKSRVCREDDEGFRVKKEEQDDRTDVNTGLNLRTTGNTKSDESMIDDGESSEMEDKRAKNELVKLQDELKKMTMDNQKLRELLTQVSNSYTSLQMHLVSLMQQQQQQNNKVIEAAEKPEETIVPRQFIDLGPTRAVGEAEDVSNSSSEDRTRSGGSSAAERRSNGKRLGREESPETESNKIQKVNSTTPTTFDQTAEATMRKARVSVRARSEAPMISDGCQWRKYGQKMAKGNPCPRAYYRCTMATGCPVRKQVQRCAEDRSILITTYEGNHNHPLPPAAVAMASTTTAAANMLLSGSMSSHDGMMNPTNLLARAVLPCSTSMATISASAPFPTVTLDLTHSPPPPNGSNPSSSAATNNNHNSLMQRPQQQQQQMTNLPPGMLPHVIGQALYNQSKFSGLQFSGGSPSTAAFSQSHAVADTITALTADPNFTAALAAVISSMINGTNHHDGEGNNKNQ

>AtWRKY10

MSDFDENFIEMTSYWAPPSSPSPRTILAMLEQTDNGLNPISEIFPQESLPRDHTDQSGQRSGLRERLAARVGFNLPTLNTEENMSPLDAFFRSSNVPNSPVVAISPGFSPSALLHTPNMVSDSSQIIPPSSATNYGPLEMVETSGEDNAAMMMFNNDLPYQPYNVDLPSLEVFDDIATEESFYIPSYEPHVDPIGTPLVTSFESELVDDAHTDIISIEDSESEDGNKDDDDEDFQYEDEDEDQYDQDQDVDEDEEEEKDEDNVALDDPQPPPPKRRRYEVSNMIGATRTSKTQRIILQMESDEDNPNDGYRWRKYGQKVVKGNPNPRSYFKCTNIECRVKKHVERGADNIKLVVTTYDGIHNHPSPPARRSNSSSRNRSAGATIPQNQNDRTSRLGRAPPTPTPPTPPPSSYTPEEMRPFSSLATEIDLTEVYMTGISMLPNIPVYENSGFMYQNDEPTMNAMPDGSDVYDGIMERLYFKFGVDM

>AtWRKY2

MAGFDENVAVMGEWVPRSPSPGTLFSSAIGEEKSSKRVLERELSLNHGQVIGLEEDTSSNHNKDSSQSNVFRGGLSERIAARAGFNAPRLNTENIRTNTDFSIDSNLRSPCLTISSPGLSPATLLESPVFLSNPLAQPSPTTGKFPFLPGVNGNALSSEKAKDEFFDDIGASFSFHPVSRSSSSFFQGTTEMMSVDYGNYNNRSSSHQSAEEVKPGSENIESSNLYGIETDNQNGQNKTSDVTTNTSLETVDHQEEEEEQRRGDSMAGGAPAEDGYNWRKYGQKLVKGSEYPRSYYKCTNPNCQVKKKVERSREGHITEIIYKGAHNHLKPPPNRRSGMQVDGTEQVEQQQQQRDSAATWVSCNNTQQQGGSNENNVEEGSTRFEYGNQSGSIQAQTGGQYESGDPVVVVDASSTFSNDEDEDDRGTHGSVSLGYDGGGGGGGGEGDESESKRRKLEAFAAEMSGSTRAIREPRVVVQTTSDVDILDDGYRWRKYGQKVVKGNPNPRSYYKCTAPGCTVRKHVERASHDLKSVITTYEGKHNHDVPAARNSSHGGGGDSGNGNSGGSAAVSHHYHNGHHSEPPRGRFDRQVTTNNQSPFSRPFSFQPHLGPPSGFSFGLGQTGLVNLSMPGLAYGQGKMPGLPHPYMTQPVGMSEAMMQRGMEPKVEPVSDSGQSVYNQIMSRLPQI

>AtWRKY40

MDQYSSSLVDTSLDLTIGVTRMRVEEDPPTSALVEELNRVSAENKKLSEMLTLMCDNYNVLRKQLMEYVNKSNITERDQISPPKKRKSPAREDAFSCAVIGGVSESSSTDQDEYLCKKQREETVVKEKVSRVYYKTEASDTTLVVKDGYQWRKYGQKVTRDNPSPRAYFKCACAPSCSVKKKVQRSVEDQSVLVATYEGEHNHPMPSQIDSNNGLNRHISHGGSASTPVAANRRSSLTVPVTTVDMIESKKVTSPTSRIDFPQVQKLLVEQMASSLTKDPNFTAALAAAVTGKLYQQNHTEK

>AtWRKY51

MNISQNPSPNFTYFSDENFINPFMDNNDFSNLMFFDIDEGGNNGLIEEEISSPTSIVSSETFTGESGGSGSATTLSKKESTNRGSKESDQTKETGHRVAFRTRSKIDVMDDGFKWRKYGKKSVKNNINKRNYYKCSSEGCSVKKRVERDGDDAAYVITTYEGVHNHESLSNVYYNEMVLSYDHDNWNQHSLLRS

>AtWRKY30

MEKNHSSGEWEKMKNEINELMIEGRDYAHQFGSASSQETREHLAKKILQSYHKSLTIMNYSGELDQVSQGGGSPKSDDSDQEPLVIKSSKKSMPRWSSKVRIAPGAGVDRTLDDGFSWRKYGQKDILGAKFPRGYYRCTYRKSQGCEATKQVQRSDENQMLLEISYRGIHSCSQAANVGTTMPIQNLEPNQTQEHGNLDMVKESVDNYNHQAHLHHNLHYPLSSTPNLENNNAYMLQMRDQNIEYFGSTSFSSDLGTSINYNFPASGSASHSASNSPSTVPLESPFESYDPNHPYGGFGGFYS

>AtWRKY60

MDYDPNTNPFDLHFSGKLPKREVSASASKVVEKKWLVKDEKRNMLQDEINRVNSENKKLTEMLARVCEKYYALNNLMEELQSRKSPESVNFQNKQLTGKRKQELDEFVSSPIGLSLGPIENITNDKATVSTAYFAAEKSDTSLTVKDGYQWRKYGQKITRDNPSPRAYFRCSFSPSCLVKKKVQRSAEDPSFLVATYEGTHNHTGPHASVSRTVKLDLVQGGLEPVEEKKERGTIQEVLVQQMASSLTKDPKFTAALATAISGRLIEHSRT

>AtWRKY65

MKRGLDMARSYNDHESSQETGPESPNSSTFNGMKALISSHSPKRSRRSVEKRVVNVPMKEMEGSRHKGDTTPPSDSWAWRKYGQKPIKGSPYPRGYYRCSSTKGCPARKQVERSRDDPTMILITYTSEHNHPWPLTSSTRNGPKPKPEPKPEPEPEVEPEAEEEDNKFMVLGRGIETTPSCVDEFAWFTEMETTSSTILESPIFSSEKKTAVSGADDVAVFFPMGEEDESLFADLGELPECSVVFRHRSSVVGSQVEIF

>AtWRKY9

MGFDFSTSKSKAKRQKRIEVRFASPLMGIDLSLKLEAEEKKKEIEGSKHSRENKEDEEHDASGDEDEQMVKEDEDDSSSLGLRTREEENEREELLQLQIQMESVKEENTRLRKLVEQTLEDYRHLEMKFPVIDKTKKMDLEMFLGVQGKRCVDITSKARKRGAERSPSMEREIGLSLSLEKKQKQEESKEAVQSHHQRYNSSSLDMNMPRIISSSQGNRKARVSVRARCETATMNDGCQWRKYGQKTAKGNPCPRAYYRCTVAPGCPVRKQVQRCLEDMSILITTYEGTHNHPLPVGATAMASTASTSPFLLLDSSDNLSHPSYYQTPQAIDSSLITYPQNSSYNNRTIRSLNFDGPSRGDHVSSSQNRLNWMM

>AtWRKY41

MEMMNWERRSLLNELIHGLKAAKQLQGSSSPSLSASSSYLTTEIKENLLHNIVSSFKKAILMLNGSTTQHNPTIELAPDPLAHPGKVPGSPASITGNPRSEEFFNVRSKEFNLSSKKRKMLPKWTEQVRISPERGLEGPHDDIFSWRKYGQKDILGAKFPRSYYRCTFRNTQYCWATKQVQRSDGDPTIFEVTYRGTHTCSQGIPLPEKRETKPKHTVAVNYQNLRASLTVRTGGLGSEAFSFPVTSPLYTYESINGGGTFYHHVGSSGPSDFTGLISTNTSTGSSPIFDVNFQFDPTAE

INTGFPTFFHNSI

>AtWRKY13

MGAINQGISLFDESQTVINPINTNHLGFFFSFPSHSTLSSSSSSSSSSPSSLVSPFLGHNSLNSFLHNNPSSFISHPQDSINLMTNLPETLISSLSSSKQRDDHDGFLNLDHHRLTGSISSQRPLSNPWAWSCQAGYGSSQKNNHGSEIDVDDNDDEVGDGGGINDDDNGRHHHHDTPSRHDKHNTASLGVVSSLKMKKLKTRRKVREPRFCFKTLSEVDVLDDGYRWRKYGQKVVKNTQHPRSYYRCTQDKCRVKKRVERLADDPRMVITTYEGRHLHSPSNHLDDDSLSTSHLHPPLSNFFW

>AtWRKY62

MNSCQQKAMEKLLHGHGCANQLLIMDQTESDSSMEREDLAKSVLHCFSDALSILIDTNDHQDDQSNNSSPQDSSPVLESSRKPLHKRGRKTSMAESSDYHRHESSTPIYHDGFLWRKYGQKQIKESEYQRSYYKCAYTKDQNCEAKKQVQKIQHNPPLYSTTYFGQHICQLHQAYATFPIDTSDFEEHEGSHMIRFGHPNISFSSSTSNLRQHQNHQDRIKDEYMKPVIAEDWSPSQWMSSEVALAVEAFEFNPFWTSHDLSS

>AtWRKY45

MEDRRCDVLFPCSSSVDPRLTEFHGVDNSAQPTTSSEEKPRSKKKKKEREARYAFQTRSQVDILDDGYRWRKYGQKAVKNNPFPRSYYKCTEEGCRVKKQVQRQWGDEGVVVTTYQGVHTHAVDKPSDNFHHILTQMHIFPPFCLKE

>AtWRKY55

MYSYKKISYQMEEVMSMIFHGMKLVKSLESSLPEKPPESLLTSLDEIVKTFSDANERLKMLLEIKNSETALNKTKPVIVSVANQMLMQMEPGLMQEYWLRYGGSTSSQGTEAMFQTQLMAVDGGGERNLTAAVERSGASGSSTPRQRRRKDEGEEQTVLVAALRTGNTDLPPDDNHTWRKYGQKEILGSRFPRAYYRCTHQKLYNCPAKKQVQRLNDDPFTFRVTYRGSHTCYNSTAPTASSATPSTIPISSVTTGHSVDYGLAVVDMADVMFGSGGVGTNMDFIFPKNDPS

>AtWRKY15

MAVELMTRNYISGVGADSFAVQEAAASGLKSIENFIGLMSRDSFNSDQPSSSSASASASAAADLESARNTTADAAVSKFKRVISLLDRTRTGHARFRRAPVHVISPVLLQEEPKTTPFQSPLPPPPQMIRKGSFSSSMKTIDFSSLSSVTTESDNQKKIHHHQRPSETAPFASQTQSLSTTVSSFSKSTKRKCNSENLLTGKCASASSSGRCHCSKKRKIKQRRIIRVPAISAKMSDVPPDDYSWRKYGQKPIKGSPHPRGYYKCSSVRGCPARKHVERAADDSSMLIVTYEGDHNHSLS

AADLAGAAVADLILESS

>AtWRKY27

MSSEDWDLFAVVRSCSSSVSTTNSCAGHEDDIGNCKQQQDPPPPPLFQASSSCNELQDSCKPFLPVTTTTTTTWSPPPLLPPPKASSPSPNILLKQEQVLLESQDQKPPLSVRVFPPSTSSSVFVFRGQRDQLLQQQSQPPLRSRKRKNQQKRTICHVTQENLSSDLWAWRKYGQKPIKGSPYPRNYYRCSSSKGCLARKQVERSNLDPNIFIVTYTGEHTHPRPTHRNSLAGSTRNKSQPVNPVPKPDTSPLSDTVKEEIHLSPTTPLKGNDDVQETNGDEDMVGQEVNMEEEEEEEEVEEDDEEEEDDDDVDDLLIPNLAVRDRDDLFFAGSFPSWSAGSAGDGGG

>AtWRKY3

MAEKEEKEPSKLKSSTGVSRPTISLPPRPFGEMFFSGGVGFSPGPMTLVSNLFSDPDEFKSFSQLLAGAMASPAAAAVAAAAVVATAHHQTPVSSVGDGGGSGGDVDPRFKQSRPTGLMITQPPGMFTVPPGLSPATLLDSPSFFGLFSPLQGTFGMTHQQALAQVTAQAVQGNNVHMQQSQQSEYPSSTQQQQQQQQQASLTEIPSFSSAPRSQIRASVQETSQGQRETSEISVFEHRSQPQNADKPADDGYNWRKYGQKQVKGSDFPRSYYKCTHPACPVKKKVERSLDGQVTEIIYKGQHNHELPQKRGNNNGSCKSSDIANQFQTSNSSLNKSKRDQETSQVTTTEQMSEASDSEEVGNAETSVGERHEDEPDPKRRNTEVRVSEPVASSHRTVTEPRIIVQTTSEVDLLDDGYRWRKYGQKVVKGNPYPRSYYKCTTPDCGVRKHVERAATDPKAVVTTYEGKHNHDVPAARTSSHQLRPNNQHNTSTVNFNHQQPVARLRLKEEQIT

>AtWRKY7

MTVELMMSSYSGGGGGGDGFPAIAAAAKMEDTALREAASAGIHGVEEFLKLIGQSQQPTEKSQTEITAVTDVAVNSFKKVISLLGRSRTGHARFRRAPASTQTPFKQTPVVEEEVEVEEKKPETSSVLTKQKTEQYHGGGSAFRVYCPTPIHRRPPLSHNNNNNQNQTKNGSSSSSPPMLANGAPSTINFAPSPPVSATNSFMSSHRCDTDSTHMSSGFEFTNPSQLSGSRGKPPLSSASLKRRCNSSPSSRCHCSKKRKSRVKRVIRVPAVSSKMADIPSDEFSWRKYGQKPIKGSPHPRGYYKCSSVRGCPARKHVERALDDAMMLIVTYEGDHNHALVLETTTMNHDKTL

>AtWRKY38

MEMNSPHEKAVQAIRYGHSCAMRLKRRLNHPMADGGPLSSYDLAKSIVESFSNAISILSAKPETEDDQFSDLSSRDSSPPPQGSPSKKRKIDSTNSSENWRDDSPDPIYYDGYLWRKYGQKSIKKSNHQRSYYRCSYNKDHNCEARKHEQKIKDNPPVYRTTYFGHHTCKTEHNLDAIFIAGQDPLDDFKSTQMIRFGKDQDQEKESRSNGFSLSVKHEEDIIKEQAIDQYREITSNDQDCQDVIEEYLSSPSGSYPPSSSSGSESADFNSDLLFDNPDSWDRYDQFYF

>AtWRKY42

MFRFPVSLGGGPRENLKPSDEQHQRAVVNEVDFFRSAEKRDRVSREEQNIIADETHRVHVKRENSRVDDHDDRSTDHINIGLNLLTANTGSDESMVDDGLSVDMEEKRTKCENAQLREELKKASEDNQRLKQMLSQTTNNFNSLQMQLVAVMRQQEDHHHLATTENNDNVKNRHEVPEMVPRQFIDLGPHSDEVSSEERTTVRSGSPPSLLEKSSSRQNGKRVLVREESPETESNGWRNPNKVPKHHASSSICGGNGSENASSKVIEQAAAEATMRKARVSVRARSEAPMLSDGCQWRKYGQKMAKGNPCPRAYYRCTMAVGCPVRKQVQRCAEDRTILITTYEGNHNHPLPPAAMNMASTTTAAASMLLSGSTMSNQDGLMNPTNLLARTILPCSSSMATISASAPFPTITLDLTESPNGNNPTNNPLMQFSQRSGLVELNQSVLPHMMGQALYYNQQSKFSGLHMPSQPLNAGESVSAATAAIASNPNFAAALAAAITSIINGSNNQQNGNNNNSNVTTSNVDNRQ

>AtWRKY47

MEEHIQDRREIAFLHSGEFLHGDSDSKDHQPNESPVERHHESSIKEVDFFAAKSQPFDLGHVRTTTIVGSSGFNDGLGLVNSCHGTSSNDGDDKTKTQISRLKLELERLHEENHKLKHLLDEVSESYNDLQRRVLLARQTQVEGLHHKQHEDVPQAGSSQALENRRPKDMNHETPATTLKRRSPDDVDGRDMHRGSPKTPRIDQNKSTNHEEQQNPHDQLPYRKARVSVRARSDATTVNDGCQWRKYGQKMAKGNPCPRAYYRCTMAVGCPVRKQVQRCAEDTTILTTTYEGNHNHPLPPSATAMAATTSAAAAMLLSGSSSSNLHQTLSSPSATSSSSFYHNFPYTSTIATLSASAPFPTITLDLTNPPRPLQPPPQFLSQYGPAAFLPNANQIRSMNNNNQQLLIPNLFGPQAPPREMVDSVRAAIAMDPNFTAALAAAISNIIGGGNNDNNNNTDINDNKVDAKSGGSSNGDSPQLPQSCTTFSTN

>AtWRKY26

MGSFDRQRAVPKFKTATPSPLPLSPSPYFTMPPGLTPADFLDSPLLFTSSNILPSPTTGTFPAQSLNYNNNGLLIDKNEIKYEDTTPPLFLPSMVTQPLPQLDLFKSEIMSSNKTSDDGYNWRKYGQKQVKGSENPRSYFKCTYPNCLTKKKVETSLVKGQMIEIVYKGSHNHPKPQSTKRSSSTAIAAHQNSSNGDGKDIGEDETEAKRWKREENVKEPRVVVQTTSDIDILDDGYRWRKYGQKVVKGNPNPRSYYKCTFTGCFVRKHVERAFQDPKSVITTYEGKHKHQIPTPRRGPVLRLLGKTET

>AtWRKY32

MEEDTGIDEAKTYTVEKSEKVEPEKDGLSQFRDEEKSLGADMEDLHDETVRETLGKDQVQGVRENSSVEPNVEDVLEVNETDSVKETVVSAIVPVDEVEENRQVETSPSLAASSDSLTVTPCLSLDPATASTAQDLPLVSVPTKQEQRSDSPVVNRLSVTPVPRTPARDGYNWRKYGQKQVKSPKGSRSYYRCTYTECCAKKIECSNDSGNVVEIVNKGLHTHEPPRKTSFSPREIRVTTAIRPVSEDDTVVEELSIVPSGSDPSASTKEYICESQTLVDRKRHCENEAVEEPEPKRRLK

KDNSQSSDSVSKPGKKNKFVVHAAGDVGICGDGYRWRKYGQKMVKGNPHPRNYYRCTSAGCPVRKHIETAVENTKAVIITYKGVHNHDMPVPKKRHGPPSSMLVAAAAPTSMRTRTDDQVNIPTSSQCSVGRESEKQSKEALDVGGEKVMESARTLLSIGFEIKQC

>AtWRKY36

MIKEETVSYFQTFDGVMAESDKEEELDATKAKVEKVREENEKLKLLLSTILNNYNSLQMQVSKVLGQQQGASSMELDHIDRQDENNDYDVDISLRLGRSEQKISKKEENKVDKISTKNVEESKDKRSALGFGFQIQSYEASKLDDLCRQVKLANAENKCVSSRKDVKSVRNENHQDVLEEHEQTGLKKTRVCVKASCEDPSINDGCQWRKYGQKTAKTNPLPRAYYRCSMSSNCPVRKQVQRCGEEETSAFMTTYEGNHDHPLPMEASHMAAGTSAAASLLQSGSSSSSSSTSASLSYFFPFHHFSISTTNSHPTVTLDLTRPNYPNQLPDDYPLSSSSFSLNFSSPDPPPPSSHDHTLNFSGLRTQAPLSTDSLLARYRTRLSGQQ

>AtWRKY57

MNDPDNPDLSNDDSAWRELTLTAQDSDFFDRDTSNILSDFGWNLHHSSDHPHSLRFDSDLTQTTGVKPTTVTSSCSSSAAVSVAVTSTNNNPSATSSSSEDPAENSTASAEKTPPPETPVKEKKKAQKRIRQPRFAFMTKSDVDNLEDGYRWRKYGQKAVKNSPFPRSYYRCTNSRCTVKKRVERSSDDPSIVITTYEGQHCHQTIGFPRGGILTAHDPHSFTSHHHLPPPLPNPYYYQELLHQLHRDNNAPSPRLPRPTTEDTPAVSTPSEEGLLGDIVPQTMRNP

>AtWRKY61

MDEAKEENRRLKSSLSKIKKDFDILQTQYNQLMAKHNEPTKFQSKGHHQDKGEDEDREKVNEREELVSLSLGRRLNSEVPSGSNKEEKNKDVEEAEGDRNYDDNEKSSIQGLSMGIEYKALSNPNEKLEIDHNQETMSLEISNNNKIRSQNSFGFKNDGDDHEDEDEILPQNLVKKTRVSVRSRCETPTMNDGCQWRKYGQKIAKGNPCPRAYYRCTIAASCPVRKQVQRCSEDMSILISTYEGTHNHPLPMSATAMASATSAAASMLLSGASSSSSAAADLHGLNFSLSGNNITPKPKTHFLQSPSSSGHPTVTLDLTTSSSSQQPFLSMLNRFSSPPSNVSRSNSYPSTNLNFSNNTNTLMNWGGGGNPSDQYRAAYGNINTHQQSPYHKIIQTRTAGSSFDPFGRSSSSHSPQINLDHIGIKNIISHQVPSLPAETIKAITTDPSFQSALATALSSIMGGDLKIDHNVTRNEAEKSP

>AtWRKY75

MEGYDNGSLYAPFLSLKSHSKPELHQGEEESSKVRSEGCSKSVESSKKKGKKQRYAFQTRSQVDILDDGYRWRKYGQKAVKNNKFPRSYYRCTYGGCNVKKQVQRLTVDQEVVVTTYEGVHSHPIEKSTENFEHILTQMQIYSSF

>AtWRKY31

MFRFPVSLGGSRDEDRHDQITPLDDHRVVVDEVDFFSEKRDRVSRENINDDDDEGNKVLIKMEGSRVEENDRSRDVNIGLNLLTANTGSDESTVDDGLSMDMEDKRAKIENAQLQEELKKMKIENQRLRDMLSQATTNFNALQMQLVAVMRQQEQRNSSQDHLLAQESKAEGRKRQELQIMVPRQFMDLGPSSGAAEHGAEVSSEERTTVRSGSPPSLLESSNPRENGKRLLGREESSEESESNAWGNPNKVPKHNPSSSNSNGNRNGNVIDQSAAEATMRKARVSVRARSEAAMISDGCQWRKYGQKMAKGNPCPRAYYRCTMAGGCPVRKQVQRCAEDRSILITTYEGNHNHPLPPAATAMASTTTAAASMLLSGSMSSQDGLMNPTNLLARAILPCSSSMATISASAPFPTITLDLTNSPNGNNPNMTTNNPLMQFAQRPGFNPAVLPQVVGQAMYNNQQQSKFSGLQLPAQPLQIAATSSVAESVSAASAAIASDPNFAAALAAAITSIMNGSSHQNNNTNNNNVATSNNDSRQ

>AtWRKY14

MCSVSELLDMENFQGDLTDVVRGIGGHVLSPETPPSNIWPLPLSHPTPSPSDLNINPFGDPFVSMDDPLLQELNSITNSGYFSTVGDNNNNIHNNNGFLVPKVFEEDHIKSQCSIFPRIRISHSNIIHDSSPCNSPAMSAHVVAAAAAASPRGIINVDTNSPRNCLLVDGTTFSSQIQISSPRNLGLKRRKSQAKKVVCIPAPAAMNSRSSGEVVPSDLWAWRKYGQKPIKGSPFPRGYYRCSSSKGCSARKQVERSRTDPNMLVITYTSEHNHPWPIQRNALAGSTRSSTSSSSNPNPSKPSTANVNSSSIGSQNTIYLPSSTTPPPTLSSSAIKDERGDDMELENVDDDDDNQIAPYRPELHDHQHQPDDFFADLEELEGDSLSMLLSHGCGGDGKDKTTASDGISNFFGWSGDNNYNNYDDQDSRSL

>AtWRKY64

MFSNIDQTAVAALLRGQGCANSLKRLLENHKLSSDSTEPLIYTILNSFSLALSFVDPPSLLPHNESSLQNMTSHVLQRSSKKKYYGAEDLEYYRDESPTPRPDDGFTWRKYGQKTIKTSPYQRCYYRCTYAKDQNCNARKRVQMIQDNPPVYRTTYLGKHVCKAVAVHDDTYGSEMIKFDQVVSESVMPQLATIDEQAITMEDEAIDHIMNQECDINDFSVDDDPFWASQFPPFSSEDIMFFDNIANLD

>AtWRKY66

MSLEIDAKAVSALLLGQGCANNLKTLLKNHETGSVSTEPLINSILDSFSFALSSQNIPRHVSQRSSKKKMCGIQGMEDSPTPAHIDGFIWRKYGQKTIKTSPHQRWYYRCAYAKDQNCDATKRVQKIQDNPPVYRNTYVGQHACEAPAYAVNNGGTYGSKMIKFDYVIPESVMPQPLSIDSQEITMEDKDTDDHILNYINEHLMEDEAYDVFPDVLGERCCFGLEPFPGLNINKS

>AtWRKY67

MVSNIDHKAMEALLRGQGCANNLKILLENGEISSVSTEPLIHTILDSFSLALSFMDSPNHPPYHESSSHNMASHMSRRSSKQVQHRRKLCVAEGLVNYNHDSRTMCPNDGFTWRKYGQKTIKASAHKRCYYRCTYAKDQNCNATKRVQKIKDNPPVYRTTYLGKHVCKAFAVHDDTYSSTMIRFDQVVPEPIMPQLTTIDHQVITVEENSAEHIMNQECDINDYLVDDDPFWASQFPPFPSSDTMFLENISAFD

>AtWRKY50

MNDADTNLGSSFSDDTHSVFEFPELDLSDEWMDDDLVSAVSGMNQSYGYQTSDVAGALFSGSSSCFSHPESPSTKTYVAATATASADNQNKKEKKKIKGRVAFKTRSEVEVLDDGFKWRKYGKKMVKNSPHPRNYYKCSVDGCPVKKRVERDRDDPSFVITTYEGSHNHSSMN

>AtWRKY20

MNPQANDRKEFQGDCSATGDLTAKHDSAGGNGGGGARYKLMSPAKLPISRSTDITIPPGLSPTSFLESPVFISNIKPEPSPTTGSLFKPRPVHISASSSSYTGRGFHQNTFTEQKSSEFEFRPPASNMVYAELGKIRSEPPVHFQGQGHGSSHSPSSISDAAGSSSELSRPTPPCQMTPTSSDIPAGSDQEESIQTSQNDSRGSTPSILADDGYNWRKYGQKHVKGSEFPRSYYKCTHPNCEVKKLFERSHDGQITDIIYKGTHDHPKPQPGRRNSGGMAAQEERLDKYPSSTGRDEKGSGVYNLSNPNEQTGNPEVPPISASDDGGEAAASNRNKDEPDDDDPFSKRRRMEGAMEITPLVKPIREPRVVVQTLSEVDILDDGYRWRKYGQKVVRGNPNPRSYYKCTAHGCPVRKHVERASHDPKAVITTYEGKHDHDVPTSKSSSNHEIQPRFRPDETDTISLNLGVGISSDGPNHASNEHQHQNQQLVNQTHPNGVNFRFVHASPMSSYYASLNSGMNQYGQRETKNETQNGDISSLNNSSYPYPPNMGRVQSGP

>AtWRKY58

MAVEDDVSLIRTTTLVAPTRPTITVPHRPPAIETAAYFFGGGDGLSLSPGPLSFVSSLFVDNFPDVLTPDNQRTTSFTHLLTSPMFFPPQSSAHTGFIQPRQQSQPQPQRPDTFPHHMPPSTSVAVHGRQSLDVSQVDQRARNHYNNPGNNNNNRSYNVVNVDKPADDGYNWRKYGQKPIKGCEYPRSYYKCTHVNCPVKKKVERSSDGQITQIIYKGQHDHERPQNRRGGGGRDSTEVGGAGQMMESSDDSGYRKDHDDDDDDDEDDEDLPASKIRRIDGVSTTHRTVTEPKIIVQTKSEVDLLDDGYRWRKYGQKVVKGNPHPRSYYKCTTPNCTVRKHVERASTDAKAVITTYEGKH

NHDVPAARNGTAAATAAAVGPSDHHRMRSMSGNNMQQHMSFGNNNNTGQSPVLLRLKEEKITI

>AtWRKY29

MDEGDLEAIVRGYSGSGDAFSGESSGTFSPSFCLPMETSSFYEPEMETSGLDELGELYKPFYPFSTQTILTSSVSLPEDSKPFRDDKKQRSHGCLLSNGSRADHIRISESKSKKSKKNQQKRVVEQVKEENLLSDAWAWRKYGQKPIKGSPYPRSYYRCSSSKGCLARKQVERNPQNPEKFTITYTNEHNHELPTRRNSLAGSTRAKTSQPKPTLTKKSEKEVVSSPTSNPMIPSADESSVAVQEMSVAETSTHQAAGAIEGRRLSNGLPSDLMSGSGTFPSFTGDFDELLNSQEFFSGY

LWNY

>AtWRKY44

MEVNDGERVVIAKPVASRPSSSSGFRTFTELLTDSVTVSPQTTCHEIVDAAIRPKTLRFNQPVAASVSCPRAEVKGIGNGMSCDDDSDSRNYVVYKPKAKLVSKATVSALANMLQGNRQQTWRQSEAVSYGKSVSQGTHRAGPNLVQKVPSFTESETSTGDRSSVDGYNWRKYGQKQVKGSECPRSYYKCTHPKCPVKKKVERSVEGQVSEIVYQGEHNHSKPSCPLPRRASSSISSGFQKPPKSIASEGSMGQDPNNNLYSPLWNNQSNDSTQNRTEKMSEGCVITPFEFAVPRSTNSNPGTSDSGCKSSQCDEGELDDPSRSKRRKNEKQSSEAGVSQGSVESDSLEDGFRWRKYGQKVVGGNAYPRSYYRCTSANCRARKHVERASDDPRAFITTYEGKHNHHLLLSPPSSSTLPFNSPQLSKQTI

>AtWRKY68

MENVGVGMPFYDLGQTRVYPLLSDFHDLSAERYPVGFMDLLGVHRHTPTHTPLMHFPTTPNSSSSEAVNGDDEEEEDGEEQQHKTKKRFKFTKMSRKQTKKKVPKVSFITRSEVLHLDDGYKWRKYGQKPVKDSPFPRNYYRCTTTWCDVKKRVERSFSDPSSVITTYEGQHTHPRPLLIMPKEGSSPSNGSASRAHIGLPTLPPQLLDYNNQQQQAPSSFGTEYINRQEKGINHDDDDDHVVKKSRTRDLLDGAGLVKDHGLLQDVVPSHIIKEEY

>AtWRKY74

MEEVEAANKAAVESCHGVLNLLSQQTNDSKSIMVETREAVCKFKRVSSLLSRGLGQRKIKKLNNNNYKFSSSLLPQHMFLESPVCSNNAISGCIPILAPKPLQIVPAGPPPLMLFNQNMCLDKSFLELKPPSSRAVDPKPYQFIHTHQQGVYSRSKSGLNLKFDGSIGASCYSPSISNGSRSFVSSLSMDGSVTDYDRNSFHLIGLPQGSDHISQHSRRTSCSGSLKCGSKSKCHCSKKRKLRVKRSIKVPAISNKIADIPPDEYSWRKYGQKPIKGSPHPRGYYKCSSVRGCPARKHVE

RCVEETSMLIVTYEGEHNHSRILSSQSAHT

>AtWRKY48

MEKKKEEDHHHQQQQQQQKEIKNTETKIEQEQEQEQKQEISQASSSSNMANLVTSSDHHPLELAGNLSSIFDTSSLPFPYSYFEDHSSNNPNSFLDLLRQDHQFASSSNSSSFSFDAFPLPNNNNNTSFFTDLPLPQAESSEVVNTTPTSPNSTSVSSSSNEAANDNNSGKEVTVKDQEEGDQQQEQKGTKPQLKAKKKNQKKAREARFAFLTKSDIDNLDDGYRWRKYGQKAVKNSPYPRSYYRCTTVGCGVKKRVERSSDDPSIVMTTYEGQHTHPFPMTPRGHIGMLTSPILDHGATTASSSSFSIPQPRYLLTQHHQPYNMYNNNSLSMINRRSSDGTFVNPGPSSSFPGFGYDMSQASTSTSSSIRDHGLLQDILPSQIRSDTINTQTNEENKK

>AtWRKY12

MEGGGRRVFSNYDLQQVTSSSTTIQENMNFLVPFEETNVLTFFSSSSSSSLSSPSFPIHNSSSTTTTHAPLGFSNNLQGGGPLGSKVVNDDQENFGGGTNNDAHSNSWWRSNSGSGDMKNKVKIRRKLREPRFCFQTKSDVDVLDDGYKWRKYGQKVVKNSLHPRSYYRCTHNNCRVKKRVERLSEDCRMVITTYEGRHNHIPSDDSTSPDHDCLSSF

>AtWRKY17

MTVDIMRLPKMEDQTAIQEAASQGLKSMEHLIRVLSNRPEERNVDCSEITDFTVSKFKKVISLLNRSGHARFRRGPVHSPPSSSVPPPVKVTTPAPTQISAPAPVSFVQANQQSVTLDFTRPSVFGAKTKSSEVVEFAKESFSVSSNSSFMSSAITGDGSVSKGSSIFLAPAPAVPVTSSGKPPLSGLPYRKRCFEHDHSEGFSGKISGSGNGKCHCKKSRKNRMKRTVRVPAVSAKIADIPPDEYSWRKYGQKPIKGSPHPRGYYKCSTFRGCPARKHVERALDDSTMLIVTYEGEHRH

HQSTMQEHVTPSVSGLVFGSA

>AtWRKY59

MNYPSNPNPSSTDFTEFFKFDDFDDTFEKIMEEIGREDHSSSPTLSWSSSEKLVAAEITSPLQTSLATSPMSFEIGDKDEIKKRKRHKEDPIIHVFKTKSSIDEKVALDDGYKWRKYGKKPITGSPFPRHYHKCSSPDCNVKKKIERDTNNPDYILTTYEGRHNHPSPSVVYCDSDDFDLNSLNNWSFQTANTYSFSHSAPY

>AtWRKY69

MHRRAAIQESDDEEDETYNDVVPESPSSCEDSKISKPTPKKRRNVEKRVVSVPIADVEGSKSRGEVYPPSDSWAWRKYGQKPIKGSPYPRGYYRCSSSKGCPARKQVERSRVDPSKLMITYACDHNHPFPSSSANTKSHHRSSVVLKTAKKEEEYEEEEEELTVTAAEEPPAGLDLSHVDSPLLLGGCYSEIGEFGWFYDASISSSSGSSNFLDVTLERGFSVGQEEDESLFGDLGDLPDCASVFRRGTVATEEQHRRCDFGAIPFCDSSR

>AtWRKY19

MSEKEELPLTLTSIGAATATSDYHQRVGSSGEGISSSSSDVDPRFMQNSPTGLMISQSSSMCTVPPGMAATPPISSGSGLSQQLNNSSSSKLCQVEGCQKGARDASGRCISHGGGRRCQKPDCQKGAEGKTVYCKAHGGGRRCEYLGCTKGAEGSTDFCIAHGGGRRCNHEDCTRSAWGRTEFCVKHGGGARCKTYGCGKSASGPLPFCRAHGGGKKCSHEDCTGFARGRSGLCLMHGGGKRCQRENCTKSAEGLSGLCISHGGGRRCQSIGCTKGAKGSKMFCKACITKRPLTIDGGGNMGGVTTGDALNYLKAVKDKFEDSEKYDTFLEVLNDCKHQGVDTSGVIARLKDLFKGHDDLLLGFNTYLSKEYQITILPEDDFPIDFLDKVEGPYEMTYQQAQTVQANANMQPQTEYPSSSAVQSFSSGQPQIPTSAPDSSLLAKSNTSGITIIEHMSQQPLNVDKQVNDGYNWQKYGQKKVKGSKFPLSYYKCTYLGCPSKRKVERSLDGQVAEIVYKDRHNHEPPNQGKDGSTTYLSGSSTHINCMSSELTASQFSSNKTKIEQQEAASLATTIEYMSEASDNEEDSNGETSEGEKDEDEPEPKRRITEVQVSELADASDRTVREPRVIFQTTSEVDNLDDGYRWRKYGQKVVKGNPYP

RFSSSKDYDVVIRYGRADISNEDFISHLRASLCRRGISVYEKFNEVDALPKCRVLIIVLTSTYVPSNLLNILEHQHTEDRVVYPIFYRLSPYDFVCNSKNYERFYLQDEPKKWQAALKEITQMPGYTLTDKSESELIDEIVRDALKVLCSADKVNMIGMDMQVEEILSLLCIESLDVRSIGIWGTVGIGKTTIAEEIFRKISVQYETCVVLKDLHKEVEVKGHDAVRENFLSEVLEVEPHVIRISDIKTSFLRSRLQRKRILVILDDVNDYRDVDTFLGTLNYFGPGSRIIMTSRNRRVFVLCKIDHVYEVKPLDIPKSLLLLDRGTCQIVLSPEVYKTLSLELVKFSNGNPQVLQFLSSIDREWNKLSQEVKTTSPIYIPGIFEKSCCGLDDNERGIFLDIACFFNRIDKDNVAMLLDGCGFSAHVGFRGLVDKSLLTISQHNLVDMLSFIQATGREIVRQESADRPGDRSRLWNADYIRHVFINDTGTSAIEGIFLDMLNLKFDANPNVFEKMCNLRLLKLYCSKAEEKHGVSFPQGLEYLPSKLRLLHWEYYPLSSLPKSFNPENLVELNLPSSCAKKLWKGKKARFCTTNSSLEKLKKMRLSYSDQLTKIPRLSSATNLEHIDLEGCNSLLSLSQSISYLKKLVFLNLKGCSKLENIPSMVDLESLEVLNLSGCSKLGNFPEISPNVKELYMGGTMIQEIPSSIKNLVLLEKLDLENSRHLKNLPTSIYKLKHLETLNLSGCISLERFPDSSRRMKCLRFLDLSRTDIKELPSSISYLTALDELLFVDSRRNSPVVTNPNANSTELMPSESSKLEILGTPADNEVVVGGTVEKTRGIERTPTILVKSREYLIPDDVVAVGGDIKGLRPPVLQLQPAMKLSHIPRGSTWDFVTHFAPPETVAPPSSSSEAREEEVETEETGAMFIPLGDKETCSFTVNKGDSSRTISNTSPIYASEGSFITCWQKGQLLGRGSLGSVYEGISADGDFFAFKEVSLLDQGSQAHEWIQQVEGGIALLSQLQHQNIVRYRGTTKDESNLYIFLELVTQGSLRKLYQRNQLGDSVVSLYTRQILDGLKYLHDKGFIHRNIKCANVLVDANGTVKLADFGLAKVMSLWRTPYWNWMAPEVILNPKDYDGYGTPADIWSLGCTVLEMLTGQIPYSDLEIGTALYNIGTGKLPKIPDILSLDARDFILTCLKVNPEERPTAAELLNHPFVNMPLPSSGSGSVSSLLRG

>AtWRKY21

MEEIEGTNRAAVESCHRVLNLLHRSQQQDHVGFEKNLVSETREAVIRFKRVGSLLSSSVGHARFRRAKKLQSHVSQSLLLDPCQQRTTEVPSSSSQKTPVLRSGFQELSLRQPSDSLTLGTRSFSLNSNAKAPLLQLNQQTMPPSNYPTLFPVQQQQQQQQQQQQQEQQQQQQQQQQQFHERLQAHHLHQQQQLQKHQAELMLRKCNGGISLSFDNSSCTPTMSSTRSFVSSLSIDGSVANIEGKNSFHFGVPSSTDQNSLHSKRKCPLKGDEHGSLKCGSSSRCHCAKKRKHRVRRSIRVPAISNKVADIPPDDYSWRKYGQKPIKGSPYPRGYYKCSSMRGCPARKHVERCLEDPAML

IVTYEAEHNHPKLPSQAITT

>AtWRKY22

MADDWDLHAVVRGCSAVSSSATTTVYSPGVSSHTNPIFTVGRQSNAVSFGEIRDLYTPFTQESVVSSFSCINYPEEPRKPQNQKRPLSLSASSGSVTSKPSGSNTSRSKRRKIQHKKVCHVAAEALNSDVWAWRKYGQKPIKGSPYPRGYYRCSTSKGCLARKQVERNRSDPKMFIVTYTAEHNHPAPTHRNSLAGSTRQKPSDQQTSKSPTTTIATYSSSPVTSADEFVLPVEDHLAVGDLDGEEDLLSLSDTVVSDDFFDGLEEFAAGDSFSGNSAPASFDLSWVVNSAATTTGGI

>AtWRKY23

MEFTDFSKTSFYYPSSQSVWDFGDLAAAERHSLGFMELLSSQQHQDFATVSPHSFLLQTSQPQTQTQPSAKLSSSIIQAPPSEQLVTSKVESLCSDHLLINPPATPNSSSISSASSEALNEEKPKTEDNEEEGGEDQQEKSHTKKQLKAKKNNQKRQREARVAFMTKSEVDHLEDGYRWRKYGQKAVKNSPFPRSYYRCTTASCNVKKRVERSFRDPSTVVTTYEGQHTHISPLTSRPISTGGFFGSSGAASSLGNGCFGFPIDGSTLISPQFQQLVQYHHQQQQQELMSCFGGVNEYLNSHANEYGDDNRVKKSRVLVKDNGLLQDVVPSHMLKEE

>AtWRKY72

MEVLLKLPSSESPLKDKFGSVQIHEANKGDGDHQELESAKAEMSEVKEENEKLKGMLERIESDYKSLKLRFFDIIQQEPSNTATKNQNMVDHPKPTTTDLSSFDQERELVSLSLGRRSSSPSDSVPKKEEKTDAISAEVNADEELTKAGLTLGINNGNGGEPKEGLSMENRANSGSEEAWAPGKVTGKRSSPAPASGGDADGEAGQQNHVKRARVCVRARCDTPTMNDGCQWRKYGQKIAKGNPCPRAYYRCTVAPGCPVRKQVQRCADDMSILITTYEGTHSHSLPLSATTMASTTSAAASMLLSGSSSSPAAEMIGNNLYDNSRFNNNNKSFYSPTLHSPLHPTVTLDLTAPQHSSSSSSSLLSLNFNKFSNSFQRFPSTSLNFSSTSSTSSNPSTLNLPAIWGNGYSSYTPYPYNNVQFGTSNLGKTVQNSQSLTETLTKALTSDPSFHSVIAAAISTMVGSNGEQQIVGPRHSISNNIQQTNTTNNNKGCGGYFSSLLMSNIMASNQTGASLDQPSSQLPPFSMFKNSSSSSSTTNFVNKEEKS

>AtWRKY56

MEGVDNTNPMLTLEEGENNNPFSSLDDKTLMMMAPSLIFSGDVGPSSSSCTPAGYHLSAQLENFRGGGGEMGGLVSNNSNNSDHNKNCNKGKGKRTLAMQRIAFHTRSDDDVLDDGYRWRKYGQKSVKNNAHPRSYYRCTYHTCNVKKQVQRLAKDPNVVVTTYEGVHNHPCEKLMETLSPLLRQLQFLSRVSDL

>AtWRKY71

MDDHVEHNYNTSLEEVHFKSLSDCLQSSLVMDYNSLEKVFKFSPYSSPFQSVSPSVNNPYLNLTSNSPVVSSSSNEGEPKENTNDKSDQMEDNEGDLHGVGESSKQLTKQGKKKGEKKEREVRVAFMTKSEIDHLEDGYRWRKYGQKAVKNSPYPRSYYRCTTQKCNVKKRVERSFQDPSIVITTYEGKHNHPIPSTLRGTVAAEHLLVHRGGGGSLLHSFPRHHQDFLMMKHSPANYQSVGSLSYEHGHGTSSYNFNNNQPVVDYGLLQDIVPSMFSKNES

>AtWRKY34

MAGIDNKAAVMGEWFDCSTTNHRKRSKAELGREFSLNYIKNEDSLQTTFQESSRGALRERIAARSGFNAPWLNTEDILQSKSLTISSPGLSPATLLESPVFLSNPLLSPTTGKLSSVPSDKAKAELFDDITTSLAFQTISGSGLDPTNIALEPDDSQDYEERQLGGLGDSMACCAPADDGYNWRKYGQKLVKGSEYPRSYYKCTHPNCEAKKKVERSREGHIIEIIYTGDHIHSKPPPNRRSGIGSSGTGQDMQIDATEYEGFAGTNENIEWTSPVSAELEYGSHSGSMQVQNGTHQFGYGDAAADALYRDENEDDRTSHMSVSLTYDGEVEESESKRRKLEAYATETSGSTRASREPRVVVQTTSDIDILDDGYRWRKYGQKVVKGNPNPRSYYKCTANGCTVTKHVERASDDFKSVLTTYIGKHTHVVPAARNSSHVGAGSSGTLQGSLATQTHNHNVHYPMPHSRSEGLATANSSLFDFQSHLRHPTGFSVYIGQSELSDLSMPGLTIGQEKLTSLQAPDIGDPTGLMLQLAAQPKVEPVSPQQGLDLSASSLICREMLSRLRQI

>AtWRKY24

MDREDINPMLSRLDVENNNTFSSFVDKTLMMMPPSTFSGEVEPSSSSSWYPESFHVHAPPLPPENDQIGEKGKELKEKRSRKVPRIAFHTRSDDDVLDDGYRWRKYGQKSVKHNAHPRSYYRCTYHTCNVKKQVQRLAKDPNVVVTTYEGVHNHPCEKLMETLNPLLRQLQFLSSFSNL

>AtWRKY28

MSNETRDLYNYQYPSSFSLHEMMNLPTSNPSSYGNLPSQNGFNPSTYSFTDCLQSSPAAYESLLQKTFGLSPSSSEVFNSSIDQEPNRDVTNDVINGGACNETETRVSPSNSSSSEADHPGEDSGKSRRKRELVGEEDQISKKVGKTKKTEVKKQREPRVSFMTKSEVDHLEDGYRWRKYGQKAVKNSPYPRSYYRCTTQKCNVKKRVERSFQDPTVVITTYEGQHNHPIPTNLRGSSAAAAMFSADLMTPRSFAHDMFRTAAYTNGGSVAAALDYGYGQSGYGSVNSNPSSHQVYHQGGEYELLREIFPSIFFKQEP

>AtWRKY39

MEEVEAANRSAIESCHGVLNLLSQRTSDPKSLTVETGEVVSKFKRVASLLTRGLGHGKFRSTNKFRSSFPQHIFLESPICCGNDLSGDYTQVLAPEPLQMVPASAVYNEMEPKHQLGHPSLMLSHKMCVDKSFLELKPPPFRAPYQLIHNHQQIAYSRSNSGVNLKFDGSGSSCYTPSVSNGSRSFVSSLSMDASVTDYDRNSFHLTGLSRGSDQQHTRKMCSGSLKCGSRSKCHCSKKRKLRVKRSIKVPAISNKIADIPPDEYSWRKYGQKPIKGSPHPRGYYKCSSVRGCPARKHVERCIDETSMLIVTYEGEHNHSRILSSQSAHT

>AtWRKY43

MNGLVDSSRDKKMKNPRFSFRTKSDADILDDGYRWRKYGQKSVKNSLYPRSYYRCTQHMCNVKKQVQRLSKETSIVETTYEGIHNHPCEELMQTLTPLLHQLQFLSKFT

>AtWRKY49

MEEEGYQWARRCGNNAVEDPFVYEPPLFFLPQDQHHMHGLMPNEDFIANKFVTSTLYSGPRIQDIANALALVEPLTHPVREISKSTVPLLERSTLSKVDRYTLKVKNNSNGMCDDGYKWRKYGQKSIKNSPNPRSYYKCTNPICNAKKQVERSIDESNTYIITYEGFHFHYTYPFFLPDKTRQWPNKKTKIHKHNAQDMNKKSQTQEESKEAQLGELTNQNHPVNKAQENTPANLEEGLFFPVDQCRPQQGLLEDVVAPAMKNIPTRDSVLTAS

>AtWRKY35

MDNFQGDLTDVVRGIGSGHVSPSPGPPEGPSPSSMSPPPTSDLHVEFPSAATSASCLANPFGDPFVSMKDPLIHLPASYISGAGDNKSNKSFAIFPKIFEDDHIKSQCSVFPRIKISQSNNIHDASTCNSPAITVSSAAVAASPWGMINVNTTNSPRNCLLVDNNNNTSSCSQVQISSSPRNLGIKRRKSQAKKVVCIPAPAAMNSRSSGEVVPSDLWAWRKYGQKPIKGSPYPRGYYRCSSSKGCSARKQVERSRTDPNMLVITYTSEHNHPWPTQRNALAGSTRSSSSSSLNPSSKSSTAAATTSPSSRVFQNNSSKDEPNNSNLPSSSTHPPFDAAAIKEENVEERQEKMEFDYNDVENTYRPELLQEFQHQPEDFFADLDELEGDSLTMLLSHSSGGGNMENKTTIPDVFSDFFDDDESSRSL

>AtWRKY63

MFSNIDHKAVAALLHGQGCANILKTVLDNCKVSSVSTEPLINTILDSFSLALSSVNSPNRQPHHESSSRDMAGLVPQRSSKKKICGVKGLEIYRDDSPNPRLDDGFTWRKYGQKTIKTSLYQRCYYRCAYAKDQNCYATKRVQMIQDSPPVYRTTYLGQHTCKAFGVHDNTYGSEMINFDQVVSESVMRQLATIGEQAVLMEDEANHIMNQEYDINDYLVDDEVFWGNEFPLFSSEDLMLF

>ppa015480m

MDCLQNPNPSSAGPYHFGESIDPSMDFDEFSDYFMLDYDVDDHQDSSSLSTVSPEKFMADRSTGSSGGATSRNSNNNMKCRNEGRRNKIEMGHRVAFRTKSELEVMDDGFKWRKYGKKSVKNSPNPRNYYKCSSGGCNVKKRVERDREDSSYVITTYDGVHNHESPCVVYYNQMPPPVDPNNIWTLRASSQSSASS

>ppa007708m

MEKRKNMEWEQKTLTSELTQGKELAKQLMNYLHPSASQEKRDFLISKILFSYEKALSLLKTDVGSDGESNHIPNTMLESPTSFGNGSPMSEISDQDCKNKNVFKKRKTMPRWTEEVKVFSGTGLDGSLDDGYSWRKYGQKDILGATYPRGYYRCTHRGTQGCLATKQVQKADADPSTMVVTYRGEHTCSQVLQLARSSALSLAKQASTGNQNATREAEKPEASQEMSFGFGAGLRVKTEDLDTREDDIFPSFSFPSTPIEPENVGDHIFCATLMDGYSPTFASPAATFEPDYLQAVSPCQMSSFGLGLDYVQTSESGLSEIISAPTSVTNSPIGDFGFSLDDLDFHHFENSESFAYES

>ppa008846m

MAVELMNFSKMEDQKAIQEAASQGLKSMEHLIRFLSHQQQTNQSSRLDCTDITDHTVSKFKKFPSSSSHPSYLQTLSLAPALNPRPSPAPAPVTTPAIVPPAPIESSYVQSQPHSMTLDFTRPNVFASNPKSTEIEFAKDSFSVSSSSSFMSSAITGDGSVSNGKQGSSIFLATAPAVSGAKPPLSTAPIKKRCHEHDHHSDDASCKYSGSGSASGSGKCHCSKRRKNRVKKTIRVPAISSKIADIPPDEYSWRKYGQKPIKGSPYPRGYYKCSTVRGCPARKHVERAPDDPAMLIVTYEGEHRHAPENVGLVFEST

>GhWRKY41

MENMWKWEQGTLVSELIQGMQLAKQLRLHLGAPSSSVESRDLLLQKILSSYEKALLILKLSRPTEQPQQNVGGKTCVPESPLTINGGPRSDDLEKDNQDIRDVSKKRKMMPRWTDQVRVSSENLLEGPHDDGYSWRKYGQKDILGAKYPRSYYRCTYRNTQNCWATKQVQRSDEDPILFEVTYRGTHTCANGNPAVPSPEKQHKSNSNGLNNTNYQPLQSQDTLSEFRAGLRVNTEGLDNKEMAPPFSFASTSFGCIKSENHSFSPSGVLDYYNIFGSFSSPFMSPDTPELNYFSVSQMNNFQGVLNTQHSKSDLTELVSANTSATNSPIMDLDFSLDQVELDPNFPFDTPGFFS

>VvWRKY11

MAVELLGFSKMDEQIAIQDAASAGLKSMEHLIRMLSHQTNQNHNMNQLDCREITDYTVSKFKKVISILNRTGHARFRRGPVSSSDSPSSSTSSVAPQTHALTPAPVTSLPVPPAAPPPASFVQRQSLTLDFTKPNLVSSNPVSSDVVSTSQFSKESFGLSQPMSSATNSSFMSSITGDGSVSNGKQGSSLFLAPAPAVSAGKPPLSSSCRKRCHEHDHSDDISGKYSSSGRCHCSKRRRSRVKKTIRVPAISSKIADIPADEYSWRKYGQKPIKGSPYPRGYYKCSSMRGCPARKHVERASDDPAMLIVTYEGEHSHSQVAMQEMIPHGGVGLVFEST

>GmWRKY13

MTVDLVGAAKMGMEENIAIQEAASAGLKSMEHLIRVLSSQIPSSASSSSNAHHHRLNLNHLDCTEITDFTVSKFKQVINLLNRTGHARFRSAPSHPSPSTSLPSQPQPQPQPQPYALTLDFAKPVMLKSNPNPNPSSTDLSVSQYSKTKDTTTFSISPPVSTTTSSFMSSITADGSVSDGKIGPAIIAAGKPPLSSSHRKRCHDATLSAGKASSSAHCHCSKRRKSRVKRMIRVPAISSKIADIPVDEYSWRKYGQKPIKGSPYPRGYYKCSSVRGCPARKHVERAQDDPNMLIVTYEGEHRHPQPRLPETAAGAGGTFAAHPV
