# Supplementary figures and images for: Comparative transcriptome analysis reveals gene expression differences between two peach cultivars under saline-alkaline stress
Source: Hereditas. 2020 Mar 31;157:9. doi: 10.1186/s41065-020-00122-4 (PMC7110815; doi:10.1186/s41065-020-00122-4)

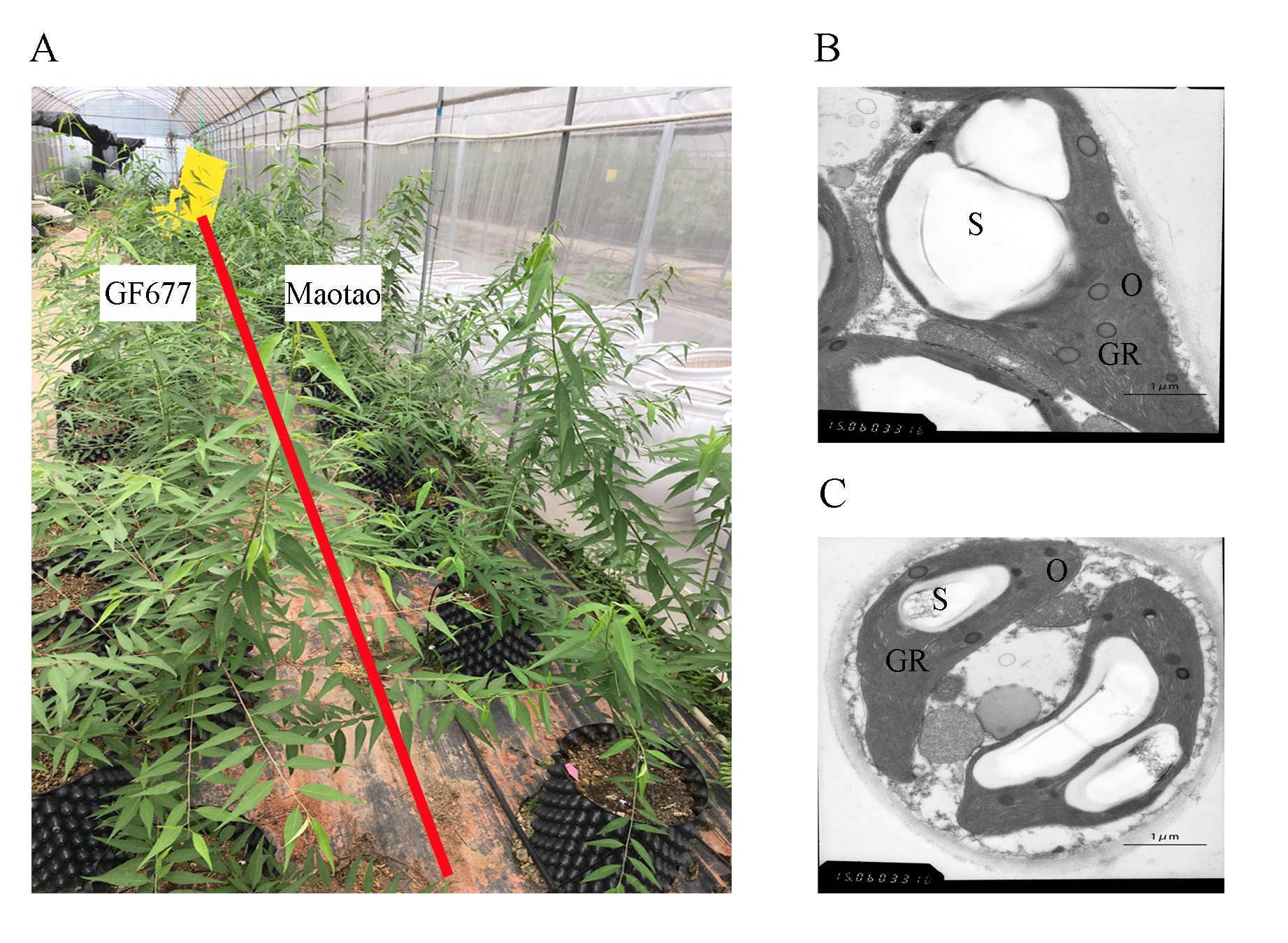

Supplement: Supplementary file 4 — Additional file 4: Figure S1. Phenotypes (A) and chloroplast ultrastructures (B-C) of GF677 and Maotao in normal soil. (B) Chloroplast ultrastructures of GF677. (C) Chloroplast ultrastructures of Maotao. The scale bar was shown 1 μm. GR: granum; S: starch grain; O: osmiophile globule [file 41065_2020_122_MOESM4_ESM.tif]
